# A Modern Framework for Identifying Novel Environmental *Legionella* Species

**DOI:** 10.3390/life16071187

**Published:** 2026-07-17

**Authors:** Karla Vasari, Maja Kovačević, Niko Kasalo, Snježana Kazazić, Ivan Mijakovic, Göran Klobučar, Damjan Franjevic, Josip Skejo, Tomislav Domazet-Lošo, Brian G. Shelton, Marina Santic, Roberta Sauerborn Klobucar

**Affiliations:** 1 PathCon Laboratories EU, 10000 Zagreb, Croatia; kvasari@pathcon.com (K.V.); mkovacevic@pathcon.com (M.K.); bshelton@pathcon.com (B.G.S.); 2 Department of Microbiology and Parasitology, Faculty of Medicine, University of Rijeka, 51000 Rijeka, Croatia; marina.santic@uniri.hr; 3 Department of Biology, Faculty of Science, University of Zagreb, 10000 Zagreb, Croatia; goran.klobucar@biol.pmf.hr (G.K.); damjan.franjevic@biol.pmf.hr (D.F.); josip.skejo@biol.pmf.hr (J.S.); 4 Laboratory of Evolutionary Genetics, Division of Molecular Biology, Ruđer Bošković Institute, 10000 Zagreb, Croatia; nkasalo@irb.hr (N.K.); tdomazet@irb.hr (T.D.-L.); 5 Laboratory for Mass Spectrometry and Functional Proteomics, Division of Physical Chemistry, Ruđer Bošković Institute, 10000 Zagreb, Croatia; snjezana.kazazic@irb.hr; 6 The Novo Nordisk Foundation Biotechnology Research Institute for the Green Transition, Technical University of Denmark, DK-2800 Kongens Lyngby, Denmark; ivan.mijakovic@chalmers.se; 7 Division of Systems and Synthetic Biology, Department of Life Sciences, Chalmers University of Technology, 41296 Göteborg, Sweden; 8 School of Medicine, Catholic University of Croatia, 10000 Zagreb, Croatia; 9 Teaching Institute of Public Health Primorje-Gorski Kotar County, 51000 Rijeka, Croatia

**Keywords:** whole genome sequencing, phylogenomics, average amino acid identity (AAI), percentage of conserved proteins (POCP), novel *Legionella* species

## Abstract

Accurate species-level identification of *Legionella* during routine environmental surveillance remains challenging, as standard proteomic screening methods like MALDI-TOF MS often lack the resolution to distinguish closely related taxa. This study introduces a novel, cost-effective, and stepwise taxonomic framework that bridges routine environmental monitoring with multi-layered, genome-resolved analysis. We expanded upon our previous screening of four novel candidate lineages by focusing on the three remaining, uncharacterized environmental isolates, while using the recently validly described *Legionella sheltonii* (strain PATHC038) as a reference framework control. By applying nucleotide- and protein-based overall genomic relatedness indices (ANI, dDDH, AAI, and POCP) alongside core-genome phylogenomics, our framework independently confirmed the genomic distinctiveness of the three isolates, which exhibited AAI values of 94.3–96.6% and POCP values of 86.5–91.0%, with POCP values lying close to the proposed species delineation threshold. By defining clear criteria for escalating isolates from culture to targeted sequencing, this workflow prevents diagnostic misidentification during epidemiological outbreak investigations and provides public health agencies with a scalable tool to monitor hidden *Legionella* diversity, ultimately enhancing proactive water safety and risk assessment.

## 1. Introduction

The genus *Legionella*, the sole genus within the family *Legionellaceae*, comprises 67 validly described species, with new taxa continuing to be identified as environmental surveillance and genome-based taxonomy advance [1]. Since its first recognition during the investigation of a pneumonia outbreak in 1976, *Legionella* has been established as an environmentally ubiquitous bacterium of major public health relevance [2,3]. Members of the genus are Gram-negative, strictly aerobic rods that require L-cysteine and iron salts for growth and are primarily associated with aquatic environments [4,5].

In natural and engineered water systems, *Legionella* species persist and replicate predominantly within free-living amoebae, an intracellular niche that enhances their ecological fitness and tolerance to environmental stressors such as disinfectants, temperature fluctuations, and nutrient limitation [6,7]. Approximately one third of described *Legionella* species are considered opportunistic human pathogens capable of causing legionellosis, a spectrum of respiratory diseases ranging from Pontiac fever to severe Legionnaires’ disease [8]. Infection typically occurs through inhalation of contaminated aerosols, linking disease risk directly to the presence and proliferation of *Legionella* in water systems [9,10]. Consequently, proactive monitoring and effective control of *Legionella* in engineered environments remain essential components of public health prevention strategies.

Engineered water systems, including cooling circuits, potable water installations, and other complex aquatic infrastructures, provide favourable conditions for *Legionella* growth through biofilm formation, stable temperatures, and stagnant water [9,11,12]. Routine environmental surveillance programs rely largely on standardized culture-based methods to detect and enumerate *Legionella*; however, these approaches offer limited resolution beyond genus-level identification. Phenotypic, serological, and proteomic methods, such as latex agglutination tests and MALDI-TOF mass spectrometry, enable rapid screening but frequently fail to reliably distinguish closely related species, particularly among non-*Legionella pneumophila* environmental isolates [13].

Recent genome-based studies, including our previous work, have demonstrated that environmental *Legionella* isolates may represent previously unrecognized species when assessed using whole genome sequencing and genome-wide relatedness metrics [14,15,16]. Despite the increasing accessibility of genomic tools, their application in routine environmental surveillance remains largely unstructured, and clear decision-making frameworks guiding their stepwise implementation are lacking.

The aim of this study was to develop a structured framework for the identification and taxonomic assessment of candidate novel environmental *Legionella* species. While previous studies, including the NOVA framework, demonstrated the utility of whole-genome sequencing for recognizing potentially novel bacterial taxa, no practical workflow has been specifically adapted for environmental *Legionella* surveillance [17]. The framework presented here integrates routine culture-based monitoring, MALDI-TOF MS, 16S rRNA gene analysis, whole-genome sequencing, nucleotide-based overall genome relatedness indices (ANI and dDDH), protein-based indices (AAI and POCP), and phylogenomic analyses into a stepwise decision-making workflow. The novelty of this study lies not in the individual analytical methods themselves, but in their structured integration into a practical framework designed for environmental surveillance and taxonomic assessment of *Legionella* isolates.

## 2. Materials and Methods

**Figure 1:**
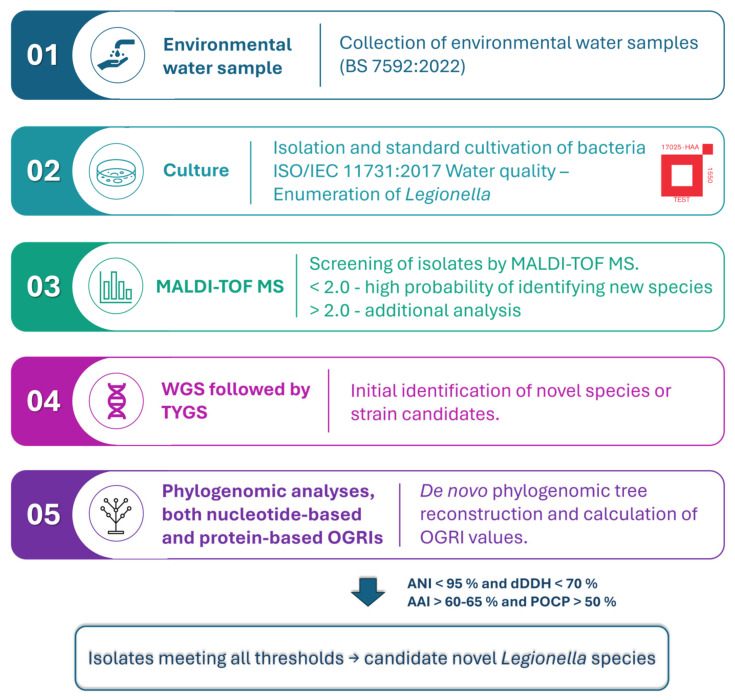
Stepwise framework for taxonomic assessment of candidate novel environmental *Legionella* species.

A framework was developed and used for the identification and characterization of potentially novel environmental *Legionella* species (Figure 1).

### 2.1. Collection of Environmental Water Samples

Environmental water samples were collected from engineered water systems as part of routine *Legionella* surveillance programme.

The study focused exclusively on environmental *Legionella* isolates recovered from water systems and did not include clinical samples or bacterial genera other than *Legionella*.

### 2.2. Isolation and Standard Cultivation of Bacteria

Laboratory culture of samples followed the methods according to ISO/IEC 11731:2017 [18] (Water quality—Enumeration of *Legionella*), as well as the procedures described in the ‘Procedures for the Recovery of *Legionella* from the Environment’ (January 2005, USDHHS, Public Health Service, CDC, Atlanta, GA, USA) [18,19]. The samples were cultivated on buffered charcoal yeast extract (BCYE) media and glycinevancomycin-polymyxin-cycloheximide (GVPC) agar and incubated at 36 °C with 3% (*v*/*v*) CO_2_ for 10 days. Presumptive *Legionella* colonies were further confirmed on culture media; BCYE agar Cys+ and BCYE Cys− agar in accordance with the ISO 11731 method in an accredited microbiological laboratory (Croatian Accreditation Agency, HAA accreditation certificate No. 1550).

### 2.3. Screenings of Isolates by MALDI-TOF MS

Species identification of bacterial isolates was performed using matrix-assisted laser desorption/ionization time-of-flight mass spectrometry (MALDI-TOF MS) (Bruker Daltonics, Bremen, Germany) with full extraction protocol as previously described [13,20]. Briefly, a loopful of a bacterial colony was suspended in 300 µL of deionized water and vortexed, after which 900 µL of absolute ethanol (Kemika, Zagreb, Croatia) was added. The suspension was centrifuged at 13,000 rpm for 2 min, the supernatant was decanted, and the resulting pellet was resuspended in an equal volume of 70% formic acid (Sigma Aldrich, Taufkirchen, Germany) and 100% acetonitrile (Fisher Chemical, Madrid, Spain), followed by a second centrifugation at 13,000 rpm for 2 min. The supernatant was spotted onto a 96-spot polished steel target plate (Bruker Daltonik, Bremen, Germany), air-dried at room temperature, and overlaid with 1 µL of a saturated solution of α-cyano-4-hydroxycinnamic acid (10 mg/mL; Bruker Daltonik, Germany) prepared in 50% acetonitrile and 2.5% trifluoroacetic acid. Mass spectra were acquired in positive linear ion mode within a mass range of 2–20 kDa. Spectral analysis and identification were performed using MBT Compass HT software version 5.0 (Bruker Daltonik) by comparison with the Bruker Biotyper reference database version 11. Species identification was interpreted based on log score values, where scores of 2.00–3.00 indicated high-confidence identification, scores of 1.70–1.99 indicated low-confidence identification, and scores below 1.70 were considered unreliable. Isolates yielding low-confidence identification scores were selected for further molecular and genomic analysis within the framework which is partial 16S rRNA gene amplification and sequence analysis.

### 2.4. Genomic Features

Bacterial 16S rRNA gene sequences were amplified via PCR using the universal bacterial primers 27F (5′-AGAGTTTGATCMTGGCTCAG-3′) and 1492R (5′-GGTTACCTTGTTACGACTT-3′) [21]. Each 25 μL reaction mixture contained 12.5 μL of 2X PCR TaqNova-RED Master Mix (Blirt, Gdańsk, Poland), 0.5 μL of each primer, 10.5 μL of sterile dH_2_O, and 1 μL of diluted bacterial cell suspension as the template. Amplification was performed in a Mastercycler nexus GX2 thermal cycler (Eppendorf, Hamburg, Germany). The thermal profile consisted of an initial denaturation at 95 °C for 5 min; followed by 40 cycles of denaturation at 95 °C for 15 s, annealing at 53 °C for 30 s, and extension at 72 °C for 30 s; with a final extension step at 72 °C for 3 min. The resulting PCR products (expected size: ~1465 bp) were resolved by gel electrophoresis on a 1% agarose gel stained with Xpert Green DNA Stain (Grisp Lda., Porto, Portugal). Following visualization, the amplicons were purified and submitted to Macrogen Europe (Amsterdam, The Netherlands) for Sanger sequencing using the same primer set. The consensus sequences obtained ranged from 1500 to 1550 bp in length. The resulting sequence data were analyzed using the Basic Local Alignment Search Tool (BLAST; NCBI web server) against the NCBI 16S ribosomal RNA database [22]. Genus-level confirmation of the bacterial isolates was established based on a sequence identity threshold of ≥97.0% with the top database match. Sequence similarity was assessed by comparison with the closest validly published bacterial species. Bacterial species were considered validly described only if they were listed as validly published in the List of Prokaryotic Names with Standing in Nomenclature (LPSN), maintained by the German Collection of Microorganisms and Cell Cultures [1].

DNA extraction, sequencing, de novo hybrid assembly and genome annotation of *Legionella* strains were performed following the methods outlined in our previous studies (Table S2) [14,23]. Genomic DNA was extracted from a single bacterial colony grown on BYCE agar. The colony was resuspended in phosphate-buffered saline (PBS), washed three times, and DNA was isolated using the DNeasy Blood and Tissue Kit (Qiagen, Hilden, Germany) with RNase treatment, following the manufacturer’s instructions. Genomic libraries for short-read sequencing were prepared using the Illumina Nextera DNA library preparation kit and sequenced on the Illumina MiSeq platform (Illumina, San Diego, CA, USA) to generate 150 bp paired-end reads. Long-read sequencing was performed using the MinION Nanopore system (Oxford Nanopore Technologies, Oxford, UK), with libraries prepared using the rapid 96 barcoding kit and sequenced on an R9.4.1 flow cell until sufficient data yield was obtained. Basecalling was carried out in real time using Guppy v5.0.11 with the high-accuracy model, and reads were exported in FASTQ format. Quality control and trimming of Illumina reads were performed using fastp (v0.20.1), while adapter trimming and quality assessment of Nanopore reads were conducted using Porechop (v0.2.4) and NanoPlot (v1.28.2), respectively. Hybrid de novo genome assembly was performed using Unicycler (v0.4.1) with default settings. Assembly quality was evaluated using QUAST (v5.2.0), and quality control reports were summarized with MultiQC. Functional genome annotation was performed using Prokka (Galaxy v1.14.6). Genome completeness and contamination were assessed using CheckM (v1.0.18, KBase), yielding values within acceptable thresholds for high-quality genome assemblies.

### 2.5. Phylogenomic and Comparative Genomic Analysis

In our previous study, several genome-based typing analyses were performed to determine average nucleotide identity (ANI) [14]. Furthermore, the calculation of genome-to-genome distance by digital DNA–DNA hybridization was conducted against *Legionella* type strains. These analyses revealed four novel species candidates: PATHC032, PATHC035, PATHC038, and PATHC039. One of these, PATHC038, has already been validly described, *Legionella sheltonii* [15]. This study expands on those findings by applying phylogenomic analyses, including protein-based overall genomic relatedness indices (OGRIs)—average amino acid identity (AAI) and percentage of conserved proteins (POCP) to further resolve species boundaries for the remaining three isolates including PATHC032, PATHC039, and PATHC035 [24]. These metrics were applied in combination to evaluate taxonomic distinctiveness, particularly in cases where nucleotide-based thresholds alone may be inconclusive.

Whole-genome sequence data were uploaded to the Type (Strain) Genome Server (TYGS) for a whole-genome-based taxonomic analysis [25]. The analysis was performed using the current TYGS pipeline, including recently implemented methodological updates and features [26,27]. Taxonomic nomenclature, synonymy, and the associated literature were obtained through the TYGS’s sister database, List of Prokaryotic names with Standing in Nomenclature [26,27].

The identification of closely related type strains was carried out using two complementary approaches. First, all submitted genomes were compared against type strain genomes available in the TYGS database using the MASH (v2.3) algorithm, which provides a rapid estimate of intergenomic relatedness [28]. For each query genome, the ten type strains with the smallest MASH distances were selected. Second, an additional set of closely related type strains was identified based on 16S rRNA gene sequences. These sequences were extracted from the query genomes using RNAmmer (v1.2) [29] and compared against 16S rRNA gene sequences of all type strains in the TYGS database using BLAST (v2.17) [30].

The 50 best-matching type strains, ranked according to bitscore, were retained for each genome, and precise intergenomic distances were subsequently calculated using the Genome BLAST Distance Phylogeny (GBDP) approach with the coverage algorithm and distance formula d5 [31]. From these results, the ten closest type strain genomes for each of the submitted genomes were selected.

For phylogenomic inference, pairwise genome comparisons among all selected genomes were conducted using GBDP with the trimming algorithm and distance formula d5 [31]. One hundred distance replicates were calculated for each comparison. Digital DNA–DNA hybridization (dDDH) values and confidence intervals were estimated using the recommended settings of the GGDC 4.0 [26,31].

Phylogenetic relationships were inferred from the resulting intergenomic distances using a balanced minimum evolution tree generated with FASTME (v2.1.6.1), including SPR post-processing [32]. Branch support was estimated from 100 pseudo-bootstrap replicates. Tree was rooted at the midpoint and visualized using PhyD3 (v1.3) [33,34].

Species-level clustering was performed using a type-based approach with a 70% dDDH threshold around each type strain [25], while subspecies clustering was determined using a 79% dDDH threshold [35].

The genomes and proteomes of isolates were evaluated alongside 36 *Legionella* type strains selected based on the availability and quality of publicly accessible genomic and proteomic data, representing the major phylogenetic diversity within the genus. For phylogenomic analyses, we ensured that the closest matches, as identified by MALDI-TOF MS, were included, enabling us to compare the proposed novel species with their closest relatives. The genome sequence data of reference type strains were obtained from the NCBI whole-genome sequencing (Table S1). AAI was calculated using the EzAAI pipeline [36]. POCP was calculated using the POCP-nf pipeline [37,38].

To establish a statistically validated Percentage of Conserved Proteins (POCP) cutoff for species delineation, we performed a Receiver Operating Characteristic (ROC) analysis. First, we obtained the pairwise POCP values for two groups: an intra-species group comprising 12 different *L. pneumophila* serogroups, and an inter-species group comprising 36 different *Legionella* species. Our newly identified strains were not included in the analysis. We then performed the ROC analysis, using these two groups as classes and the pairwise POCP values as the predictor variable. We evaluated the diagnostic ability of the POCP metric by calculating the Area Under the Curve (AUC). To identify the optimal classification threshold, we calculated the maximum Youden’s J statistic, representing the POCP value that provides the best trade-off between true positive and false positive rates. To assess the robustness of this threshold, we calculated the 95% confidence interval using bootstrap resampling with 10,000 iterations. All statistical analyses and visualizations were conducted in R using the pROC and ggplot2 packages.

The de novo phylogenomic analyses were conducted with proteomic data using PhyloPhlAn 3.0 [39]. To reconstruct the phylogenomic tree, we used the supermatrix approach (concatenation of individual gene alignments, see [39]) on 400 universal marker genes, as identified by [40] and 100 bootstrap replicates, with the diversity parameter set to “low” and other parameters set to their default values. The resulting tree was graphically adapted using iToL [41].

Isolates showing ANI values below 95% and dDDH values below 70%, while maintaining AAI values above 60–65% and POCP values above 50%, and forming distinct clusters, were considered candidates for novel taxa within the genus *Legionella*. Additionally, as a supplementary novel approach, we considered POCP values of about 87.5% or lower as indicative of significant species-level divergence.

## 3. Results

All isolates were successfully cultured under conventional aerobic conditions in accordance with ISO/IEC 11731:2017 and confirmed as members of the genus *Legionella* during the initial identification steps of the workflow. Preliminary identification using MALDI-TOF mass spectrometry enabled rapid genus-level assignment for all isolates.

Partial 16S rRNA gene sequencing confirmed affiliation with the genus *Legionella* for all isolates but did not provide sufficient resolution to reliably distinguish closely related species (Table 1). Consequently, these isolates progressed to genome-based analysis within the proposed guidelines.

Whole genome sequencing followed by comparative genomic analyses revealed clear differentiation among the four isolates. All isolates, except PATHC039, which approached the ANI threshold, exhibited levels of genomic divergence that fell outside accepted species delineation thresholds when compared with their closest validly described relatives (Table 2).

**Figure 2:**
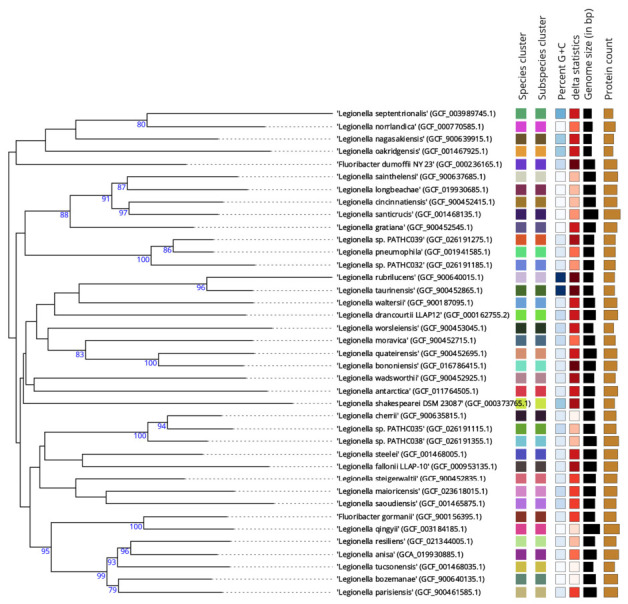
TYGS phylogenomic tree showing the placement of candidate novel *Legionella* isolates relative to reference type strains genomes. Phylogenomic tree was generated by TYGS based on Genome BLAST Distance Phylogeny (GBDP) [25]. Tree inferred with FastME 2.1.6.1 [32] from GBDP distances calculated from genome sequences. Branch lengths correspond to genomic distances between the analyzed strains. The numbers in blue above branches are GBDP pseudo-bootstrap support values >60% from 100 replications, with an average branch support of 57.1%. The tree was rooted at the midpoint [33]. The metadata columns on the right indicate species cluster assignment, subspecies cluster assignment, percent G+C content, delta statistics, genome size (bp), and protein count.

To put the observed genomic divergence into the phylogenetic context, we conducted a whole-genome-based TYGS analysis. The results, particularly TYGS species clustering, suggest that isolates PATHC032, PATHC035, and PATHC039 likely represent novel species (Figure 2). Although phylogenomic analyses consistently recovered PATHC039 as a distinct lineage, its ANI values slightly exceeded the conventional 95% threshold while the dDDH value remained below the accepted 70% cut-off. Therefore, PATHC039 is more appropriately regarded as a candidate novel species requiring further taxonomic validation. The TYGS analysis showed that none of the three isolates clustered with any currently validly described *Legionella* species, and all formed distinct phylogenomic lineages within the genus (Figure 2, Supplementary File S1).

**Figure 3:**
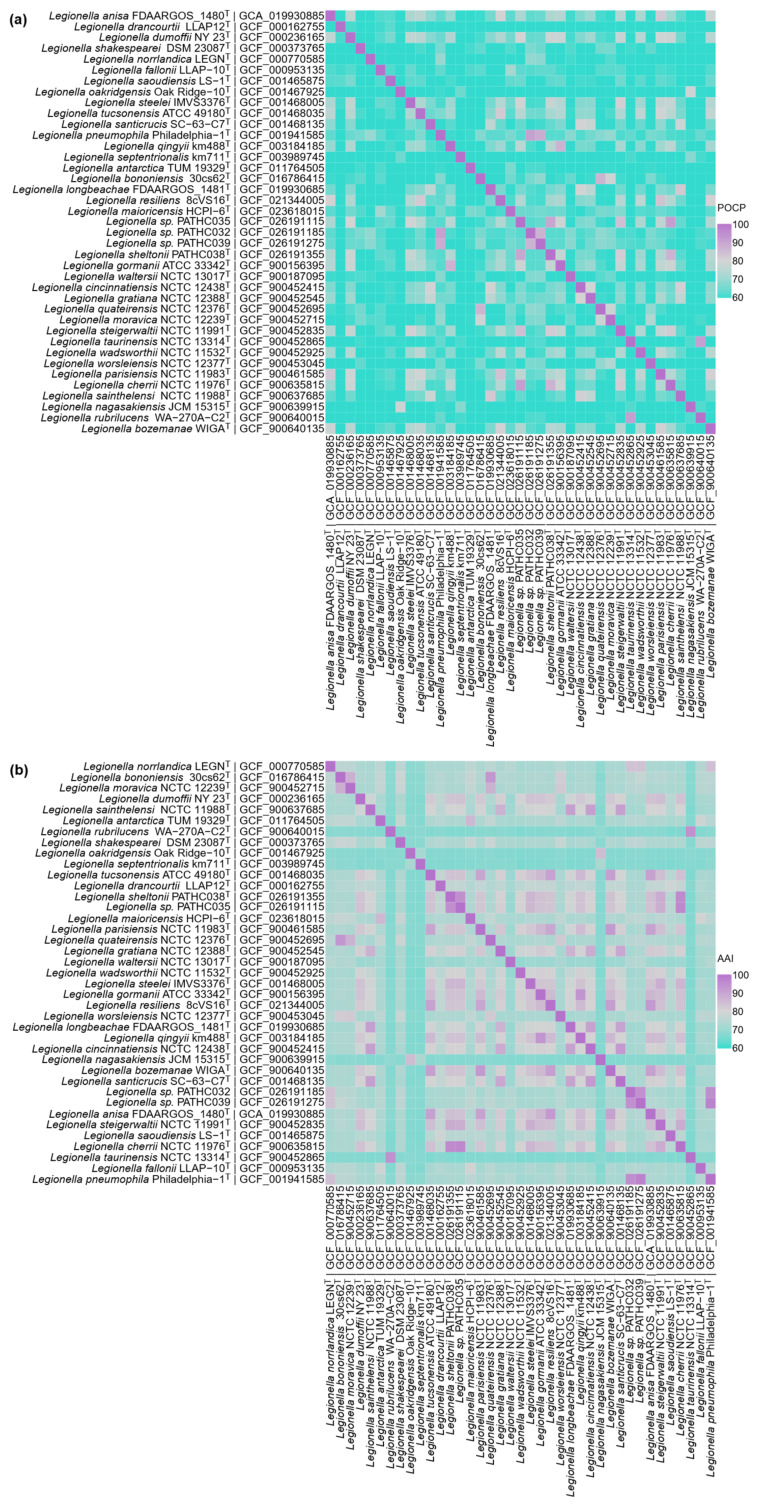
Heatmap of pairwise Percentage Of Conserved Proteins (POCP) (**a**) and pairwise Average Amino Acid Identity (AAI) (**b**) values of isolates PATHC032, PATHC039, PATHC035, and 36 *Legionella* type strains. The pairwise values are displayed as percentages between 60% (blue, low similarity) and 100% (purple, high similarity).

Protein-based OGRIs further supported these findings. AAI values ranged from 94.3% to 96.6%, closely matching ANI values, while POCP values ranged from 86.5% to 91.0%, indicating a substantial fraction of proteins lacking detectable homologs between genomes, consistent with distinct species-level divergence. Protein-based OGRIs analyses indicated that the three unresolved isolates were distinct from all currently validly described *Legionella* species, while maintaining relatedness consistent with placement within the genus (Table 2, Figure 3).

**Figure 4:**
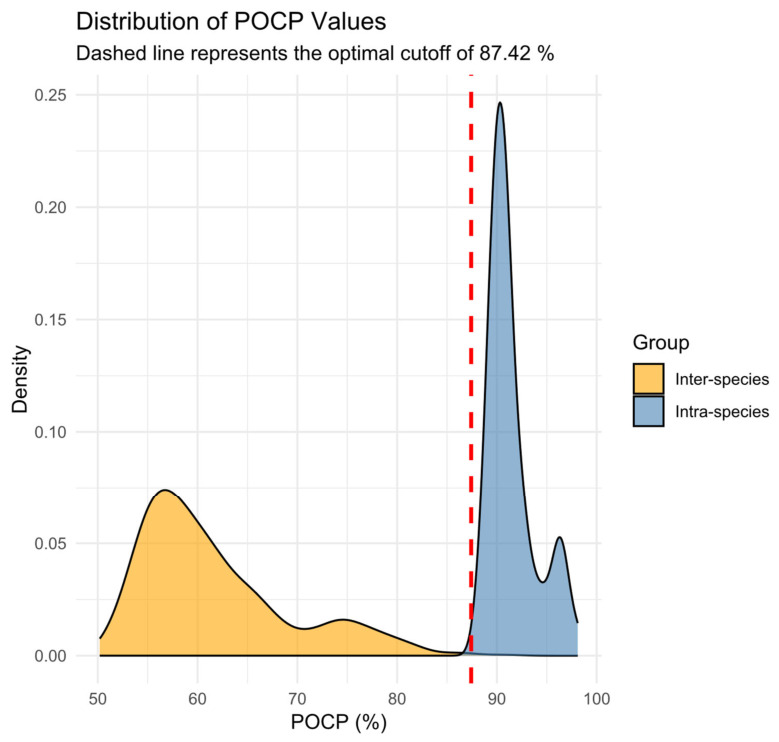
Density distribution of pairwise Percentage of Conserved Proteins (POCP) values between intra- and inter-species groups. The plot displays the overlapping density curves for pairwise POCP values of inter-species pairs (orange) and intra-species pairs (blue). The *x*-axis represents the calculated POCP values, and the *y*-axis represents the relative density of the observations. The vertical dashed red line denotes the optimal diagnostic cutoff of 87.42%, determined by maximizing Youden’s J statistic in the ROC analysis. Values above this threshold strongly indicate that the compared genomes belong to the same species, while values below indicate distinct species.

ROC analysis of the curated genomic pairs demonstrated excellent discriminatory power for the POCP metric between intra-species and inter-species groups, yielding an Area Under the Curve (AUC) of 0.999181 (Figure 4). Youden’s J statistic indicated that an optimal POCP cutoff for species delineation was 87.42% (95% CI: 85.81%, 87.78%). Applying this threshold yielded a sensitivity of 1 and a specificity of 0.9984. Therefore, newfound strains exhibiting a pairwise POCP value below ~87.5% against known type strains can be classified as likely novel species, provided that this is accompanied by other indices.

**Figure 5:**
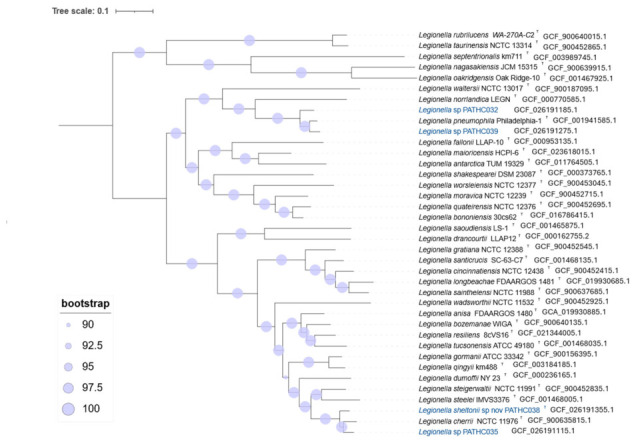
Phylogenomic tree of 36 *Legionella* type strains and three uncharacterized isolates (highlighted in blue). The tree was constructed in PhyloPhlAn 3.0 [39] using the supermatrix approach on 400 universal marker genes. Diversity parameter set to “low” and other parameters set to their default values. The tree was rooted at the midpoint.

To contextualize the observed proteome-level divergence, we reconstructed a whole-proteome phylogenomic tree of the available *Legionella* type strains (Figure 5). Even at this more conservative level, the total amount of inferred evolutionary change separating the proposed novel species from their closest relatives is relatively large, comparable to some other pairs of sister species, e.g., *L. rubrilucens* and *L. taurinensis*, and *L. gormanii* and *L. qingyii*.

The overall topology of the PhyloPhlAn (proteomic) and TYGS (genomic) trees was largely congruent at the level of shallow and intermediate nodes, with both trees showing highly similar groups of species. However, some differences in deeper branching patterns were observed between the two approaches, suggesting that phylogenetic relationships among more distantly related *Legionella* lineages may still require further resolution through expanded taxon sampling and additional genome-based analyses. Regardless, similar compositions of shallow nodes in both analyses support the robustness of the phylogenomics approach for separating species, even if they do not recover identical phylogenies at deeper timescales.

These results, together with genome-wide comparisons, support the taxonomic distinctiveness of PATHC032 and PATHC035 and further support the recognition of PATHC039 as a candidate novel *Legionella* species requiring additional validation.

Overall, the application of our proposed framework enabled clear separation of a known *Legionella* species from three environmentally derived isolates representing putative novel species, demonstrating the effectiveness of the stepwise approach for resolving taxonomic uncertainty in environmental *Legionella* surveillance.

## 4. Discussion

Routine environmental surveillance is essential for monitoring the presence of *Legionella* in engineered water systems; however, species-level identification remains challenging when relying exclusively on conventional laboratory methods. In this study, we present a structured framework that integrates standardized culture-based detection with genome-resolved analyses to improve taxonomic resolution of environmental *Legionella* isolates. Application of this workflow highlights both the limitations of routinely used identification tools and the added value of a stepwise genomic approach within environmental microbiology.

An important distinction between the present study and our previous genomic investigation [14] is the shift from isolate characterization to framework development. While the previous study established initial genomic evidence that several environmental isolates may represent previously undescribed taxa, it did not provide a structured workflow for taxonomic decision-making nor include the protein-based and phylogenomic analyses presented here. The present study builds upon those findings by integrating complementary genomic approaches into a practical framework for environmental *Legionella* surveillance and taxonomic assessment.

Culture-based isolation in accordance with ISO/IEC 11731 remains indispensable for the detection of *Legionella* in water samples and provides the foundation for downstream analyses. Consistent with previous reports, phenotypic confirmation and MALDI-TOF MS enabled rapid species-level identification of all isolates recovered in this study.

Despite yielding high-confidence MALDI-TOF MS identification scores (≥2.0), isolates were shown by genome-based analyses to be genomically distinct from their assigned reference species. This apparent discrepancy reflects intrinsic limitations of proteomic identification when applied to environmental *Legionella* isolates and does not represent a contradiction between methods.

MALDI-TOF MS identification relies on spectral similarity of a limited set of the dominant ribosomal protein profiles and provides the best possible match within the constraints of the reference database [42]. Consequently, a high-confidence score indicates strong similarity to the closest available reference spectrum rather than definitive species-level relatedness [42,43]. The limitations of MALDI-TOF MS are further exacerbated by the incomplete representation of environmental *Legionella* diversity in current reference databases, which are predominantly populated by clinically relevant species and type strains. Environmental isolates representing previously uncharacterized lineages are therefore forced to cluster with the most similar available reference, resulting in apparently high-confidence identifications that mask underlying genomic divergence. In contrast, whole genome-based metrics, including ANI, dDDH, AAI and POCP, evaluate relatedness across the entire genome or proteome and are internationally accepted standards for prokaryotic species delineation [24]. The consistently subthreshold values obtained for these indices provide robust evidence that the investigated isolates represent distinct genomic lineages within the genus *Legionella*, despite their proteomic similarity to known species. These findings highlight the necessity of genome-resolved analyses for accurate species-level assessment and support the use of MALDI-TOF MS as a rapid screening tool rather than a definitive taxonomic method in environmental *Legionella* surveillance.

Partial 16S rRNA gene sequencing provided additional confirmation of genus affiliation, but as expected, offered limited discriminatory power among closely related *Legionella* species. The high degree of sequence conservation within the genus restricts the utility of this marker for species delineation, particularly in environmental isolates that may represent previously uncharacterized taxa. Within our proposed framework, 16S rRNA gene analysis therefore functions primarily as a screening and decision-making step rather than a definitive identification tool.

Whole genome sequencing constituted the critical resolution step of the NOVA algorithm, enabling comprehensive assessment of genomic relatedness across multiple levels [17]. Nucleotide-based comparisons using ANI and dDDH demonstrated that all investigated isolates fell below established species delineation thresholds relative to their closest validly described relatives. Notably, the ANI value for isolate PATHC039 approached the ANI threshold, a scenario that complicates species assignment as genomic databases expand and phylogenetically proximate taxa are described. These findings illustrate the limitations of relying on single genomic metrics and support the use of complementary approaches for robust taxonomic interpretation. PATHC039 deserves particular attention because it illustrates one of the challenges increasingly encountered in genome-based bacterial taxonomy. While ANI values slightly exceeded the commonly applied 95% threshold, the corresponding dDDH value remained below the accepted 70% boundary, and independent phylogenomic analyses consistently recovered the isolate as a distinct lineage. Similar borderline cases have been reported in other bacterial genera and emphasize that species delineation should not rely exclusively on a single metric. Therefore, although PATHC039 cannot currently be regarded as an unequivocally novel species, the available evidence supports its classification as a candidate novel species requiring additional taxonomic and phenotypic investigation.

To address this complexity, protein-based overall genome relatedness indices (OGRIs), including AAI and POCP, were applied in combination with phylogenomic analysis based on conserved marker genes. While ANI and dDDH remain the primary criteria for species delineation, our Receiver Operating Characteristic (ROC) analysis demonstrated that POCP also possesses excellent discriminatory power for distinguishing intra-species from inter-species relationships within the genus *Legionella* (AUC = 0.999). Based on this analysis, an optimal POCP cutoff of 87.42% (95% CI: 85.81–87.78%) was identified, providing a statistically supported reference value for interpreting protein-level genomic relatedness. Nevertheless, this threshold should be regarded as supportive rather than definitive, as its broader applicability requires validation across a wider range of taxa.

Application of this statistically derived cutoff further highlighted the complexity of species delineation among the investigated isolates. For the isolate related to *L. sheltonii*, all genomic indices, including ANI, dDDH, AAI, and POCP, were concordant and consistently supported its taxonomic classification. In contrast, the remaining isolates exhibited discordant genomic signals. Although the POCP values of PATHC032 and PATHC035 exceeded the proposed threshold, their nucleotide-based indices (ANI and dDDH), together with phylogenomic analyses, supported their recognition as distinct taxa. Conversely, PATHC039 fell below the statistically derived POCP threshold, supporting its interpretation as a candidate novel species; however, its ANI value exceeded the conventional 95% species threshold while its dDDH value remained below 70%, resulting in conflicting evidence for species delineation.

AAI values between PATHC032 and PATHC039 are close to the species threshold, suggesting they may represent closely related taxa or variants of *L. pneumophila*. The discrepancy between identity-based metrics (ANI, AAI) and gene-content similarity suggests that *Legionella* species share a conserved core gene set while maintaining species-specific adaptations, reflected in relatively low pairwise POCP values.

These observations demonstrate that no single genomic metric is sufficient for the reliable identification of novel *Legionella* species, particularly in borderline cases where different indices yield partially conflicting taxonomic signals. Rather, robust species assignment should rely on an integrated evaluation of complementary nucleotide-based, protein-based, and phylogenomic approaches, which represents the central concept of the proposed framework. The identification of several isolates with inconclusive genomic classifications further suggests that borderline strains should undergo additional phenotypic, biochemical, and ecological characterization before definitive taxonomic conclusions are reached.

Overall, the concordance of nucleotide-based and protein-based OGRIs, together with phylogenomic evidence, strengthens confidence in species-level inference while providing a structured approach for interpreting ambiguous taxonomic cases. These findings demonstrate the utility of integrated genomic analyses for environmental *Legionella* identification and support the use of multi-parameter decision-making in future taxonomic investigations.

Importantly, our proposed framework is designed as an extension of the NOVA algorithm. By defining transparent criteria for progression to genome-based analyses—such as low-confidence MALDI-TOF MS identification—the workflow enables targeted use of sequencing resources while maintaining compatibility with existing monitoring programs.

The recovery of genomically distinct *Legionella* isolates from diverse engineered water systems and geographical settings highlights the substantial and likely underestimated diversity of environmental *Legionella*. Although the pathogenic potential of these putative novel species remains unknown, their identification contributes to a more comprehensive understanding of *Legionella* ecology and evolution in engineered environments. Systematic recognition of such taxa is an important prerequisite for future studies addressing virulence-associated traits, host interactions and public health relevance.

In summary, our proposed guidelines, similar to the conclusions of the NOVA study, provide a reproducible and scalable framework for improving the taxonomic resolution of environmental *Legionella* isolates. By integrating standardized culture-based methods with genome-resolved analyses, this approach supports accurate species-level placement and facilitates the systematic detection of novel *Legionella* taxa. As genomic technologies become increasingly accessible, structured workflows such as the one described here may play an important role in advancing environmental microbiology and water safety research.

### Limitations and Future Perspectives

Several limitations of this study should be acknowledged. First, although representative *Legionella* type strains covering major phylogenetic diversity were included, not all validly described species could be incorporated into the comparative analyses because high-quality genome assemblies were not available for every taxon. Consequently, future studies should reassess the proposed framework as additional reference genomes become available.

Second, the framework was demonstrated using a limited number of environmental isolates. While sufficient for proof-of-concept validation, broader application to larger collections of environmental and clinical isolates will be necessary to fully evaluate its robustness and reproducibility.

Third, the study focused on taxonomic identification and did not investigate virulence-associated traits, intracellular replication capacity, pathogenic potential, or ecological interactions with protozoan hosts. Future studies should combine genomic classification with phenotypic characterization to better understand the public health relevance of newly identified *Legionella* lineages.

Finally, the borderline taxonomic position of isolate PATHC039 highlights the need for continued refinement of species delineation criteria and demonstrates the value of integrating multiple complementary genomic and phylogenomic approaches rather than relying on a single threshold-based metric.

## 5. Conclusions

The proposed framework bridges routine environmental monitoring and high-resolution microbial taxonomy. By combining standardized isolation methods with genome-based analyses, it enables reliable detection, taxonomic assessment, and recognition of candidate novel *Legionella* species. Application of the framework successfully differentiated one previously described species, two strongly supported candidate novel species, and one additional candidate lineage requiring further taxonomic validation. As genomic technologies become increasingly accessible, this workflow may provide a practical foundation for future environmental surveillance, taxonomic research, and investigations into the ecological and pathogenic significance of environmental *Legionella* diversity.

## Figures and Tables

**Table 1:** List of 4 environmental *Legionella* isolates representing novel taxa. These results have been adapted from our previous study, except for the GenBank accession numbers corresponding to the 16S rRNA gene sequences of isolates PATHC032, PATHC035, and PATHC039, which were generated in the present study [14].

Sample ID Number	MALDI Biotyper Identification	MALDI Score	GenBank Accession Number		Isolation Source	Origin
			Whole-Genome Sequences	16S rRNA Genes		
PATHC032	*L. pneumophila*	2.19	GCF_026191185.1	PZ416333	Hot water tap	United Arab Emirates
PATHC039	*L. pneumophila*	2.22	GCF_026191275.1	PZ416335	Shower	Cruise ship
PATHC035	*L. cherrii*	2.30	GCF_026191115.1	PZ416334	Sink	Nigeria
PATHC038 (*L. sheltonii*)	*L. cherrii*	1.84	GCF_026191355.1	PQ120583	Hose bib	Cruise ship

**Table 2:** Summary of genome-based evidence supporting candidate novel *Legionella* species status. The data for nucleotide-based OGRIs (fastANI, ANIb, dDDH) were taken from our previous study [14].

Isolate Name	Closes Hit	fastANI	ANIb	dDDH	AAI	POCP
PATHC032	*L. pneumophila*	93.51	93.09	52.3	94.3	90.4
PATHC039	*L. pneumophila*	95.97	95.80	68.1	96.6	87.9
PATHC035	*L. cherrii*	94.3	93.97	56.2	95.5	91.0
PATHC038 (*L. sheltonii*)	*L. cherrii*	93.77	93.94	54.7	95.1	86.5

Closest hit columns indicate the species most closely related as determined by fastANI, ANIb and Digital-DNA–DNA Hybridization (dDDH). For ANI-based search, a novel species threshold is similarity below 95%, while dDDH has a threshold of 70% [2]; thresholds for genus delineation → AAI (>60–65%); POCP (>50%). AAI and POCP thresholds are primarily used for genus-level assessment. POCP values reported in this study are presented as supportive comparative evidence and not as formal species delineation criteria.

## Data Availability

Data is contained within the article or Supplementary Materials. The original contributions presented in this study are included in the article/Supplementary Materials. Further inquiries can be directed to the corresponding author.

## Supplementary Materials

The following supporting information can be downloaded at: https://www.mdpi.com/article/10.3390/life16071187/s1, Table S1: NCBI reference sequences and genome assemblies accession numbers of *Legionella* type strains used for phylogenomic analyses in this study, Table S2: General characteristics of the genome assemblies of the isolates; Supplementary File S1: TYGS job results. References cited in Supplementary File S1 are included in the main References list.

## Abbreviations

The following abbreviations are used in this manuscript:

MALDI-TOF	Matrix-assisted laser desorption/ionization time-of-flight
BCYE	Buffered charcoal yeast extract
GVPC	Glycine-vancomycin-polymyxin-cycloheximide
HAA	Croatian Accreditation Agency
ANI	Average nucleotide identity
dDDH	Digital DNA–DNA hybridization
AAI	Average amino acid identity
POCP	Percentage of conserved proteins
ROC	Receiver Operating Characteristic
AUC	Area Under the Curve
NCBI	National Center for Biotechnology Information
TYGS	Type (Strain) Genome Server
