# Supplementary material for: A Modern Framework for Identifying Novel Environmental Legionella Species
Source: Life (Basel). 2026 Jul 17;16(7):1187. doi: 10.3390/life16071187 (PMC13412451; doi:10.3390/life16071187)
Supplement: Supplementary file 1 [file life-16-01187-s001.zip › Supplementary File S1_TYGS_job_results.pdf]

TYPE (STRAIN) GENOME SERVER

JOB SUMMARY

PRINT DATE: 2026-05-13 14:00:49 +0200

JOB ID: 8acb9d4e-9248-44cf-a1bf-1d881f6970ea

RESULT PAGE: https://tygs.dsmz.de/user_results/show?guid=8acb9d4e-9248-44cf-a1bf-1d881f6970ea

Table 1: Phylogenies

Publication-ready versions of both the genome-scale GBDP tree and the 16S rRNA gene sequence tree can be customized and exported either in SVG (vector graphic) or PNG format from within the phylogeny viewers in your TYGS result page. For publications the **SVG format is recommended** because it is lossless, always keeps its high resolution and can also be easily converted to other popular formats such as PDF or EPS. Please follow the link provided above!

Table 2: Identification

Note: the identification of your genomes is not possible in the currently chosen mode because the analysis was restricted to user genomes only.

Table 3: Pairwise comparisons of user genomes vs. type-strain genomes

The following table contains the pairwise dDDH values between your user genomes and the selected type-strain genomes. The dDDH values are provided along with their confidence intervals (C.I.) for the three different GBDP formulas:

- formula d_0 (a.k.a. GGDC formula 1): length of all HSPs divided by total genome length
- formula d_4 (a.k.a. GGDC formula 2): sum of all identities found in HSPs divided by overall HSP length
- formula d_6 (a.k.a. GGDC formula 3): sum of all identities found in HSPs divided by total genome length

Note: Formula d_4 is independent of genome length and is thus robust against the use of incomplete draft genomes. For other reasons for preferring formula d_4, see the FAQ.

Query	Subject	d_0	C.I. d_0	d_4	C.I. d_4	d_6	C.I. d_6	Diff. G+C Percent
**'Legionella pneumophila' (GCF_001941585.1)**	**'Legionella sp. PATHC039' (GCF_026191275.1)**	78.2	[74.2 - 81.7]	67.8	[64.8 - 70.7]	79.0	[75.6 - 82.1]	0.19
**'Legionella sp. PATHC035' (GCF_026191115.1)**	**'Legionella cherrii' (GCF_900635815.1)**	89.6	[86.3 - 92.2]	56.2	[53.5 - 59.0]	85.6	[82.5 - 88.3]	0.54
**'Legionella sp. PATHC038' (GCF_026191355.1)**	**'Legionella cherrii' (GCF_900635815.1)**	76.0	[72.0 - 79.6]	54.7	[51.9 - 57.4]	73.8	[70.3 - 77.0]	0.07
**'Legionella sp. PATHC035' (GCF_026191115.1)**	**'Legionella sp. PATHC038' (GCF_026191355.1)**	78.0	[74.0 - 81.5]	48.8	[46.2 - 51.4]	73.5	[70.0 - 76.7]	0.61
**'Legionella pneumophila' (GCF_001941585.1)**	**'Legionella sp. PATHC032' (GCF_026191185.1)**	83.7	[79.9 - 86.9]	47.7	[45.1 - 50.3]	77.8	[74.3 - 80.9]	0.3
**'Legionella taurinensis' (GCF_900452865.1)**	**'Legionella rubrilucens' (GCF_900640015.1)**	84.8	[81.0 - 87.9]	45.9	[43.3 - 48.4]	77.9	[74.4 - 81.0]	0.52
**'Legionella sp. PATHC032' (GCF_026191185.1)**	**'Legionella sp. PATHC039' (GCF_026191275.1)**	73.9	[69.9 - 77.6]	45.6	[43.1 - 48.2]	69.0	[65.6 - 72.3]	0.11
**'Legionella qingyii' (GCF_003184185.1)**	**'Fluoribacter gormanii' (GCF_900156395.1)**	59.7	[56.1 - 63.3]	45.2	[42.7 - 47.8]	57.3	[54.1 - 60.4]	0.28

Query	Subject	d_0	C.I. d_0	d_4	C.I. d_4	d_6	C.I. d_6	Diff. G+C Percent
**'Legionella bononiensis' (GCF_016786415.1)**	**'Legionella quateirensis' (GCF_900452695.1)**	67.2	[63.3 - 70.8]	44.1	[41.6 - 46.7]	63.0	[59.7 - 66.2]	0.02
**'Legionella santicrucis' (GCF_001468135.1)**	**'Legionella cincinnatiensis' (GCF_900452415.1)**	54.0	[50.5 - 57.4]	43.6	[41.1 - 46.2]	52.0	[48.9 - 55.1]	0.21
**'Legionella anisa' (GCA_019930885.1)**	**'Legionella resiliens' (GCF_021344005.1)**	56.2	[52.6 - 59.7]	43.0	[40.4 - 45.5]	53.7	[50.5 - 56.7]	0.13
**'Legionella parisiensis' (GCF_900461585.1)**	**'Legionella bozemanae' (GCF_900640135.1)**	58.7	[55.1 - 62.2]	41.5	[39.0 - 44.1]	55.3	[52.1 - 58.4]	0.06
**'Legionella tucsonensis' (GCF_001468035.1)**	**'Legionella bozemanae' (GCF_900640135.1)**	50.8	[47.4 - 54.3]	39.3	[36.8 - 41.8]	48.2	[45.1 - 51.2]	0.5
**'Legionella anisa' (GCA_019930885.1)**	**'Legionella tucsonensis' (GCF_001468035.1)**	46.9	[43.5 - 50.3]	38.8	[36.4 - 41.4]	44.8	[41.8 - 47.8]	0.87
**'Legionella anisa' (GCA_019930885.1)**	**'Legionella bozemanae' (GCF_900640135.1)**	48.1	[44.7 - 51.6]	38.6	[36.1 - 41.1]	45.7	[42.7 - 48.8]	0.37
**'Legionella tucsonensis' (GCF_001468035.1)**	**'Legionella resiliens' (GCF_021344005.1)**	56.6	[53.0 - 60.1]	38.1	[35.6 - 40.6]	52.4	[49.3 - 55.4]	0.75
**'Legionella resiliens' (GCF_021344005.1)**	**'Legionella bozemanae' (GCF_900640135.1)**	55.1	[51.6 - 58.6]	37.8	[35.3 - 40.3]	51.1	[48.0 - 54.2]	0.24
**'Legionella longbeachae' (GCF_019930685.1)**	**'Legionella sainthelensi' (GCF_900637685.1)**	62.0	[58.2 - 65.5]	37.2	[34.8 - 39.8]	56.2	[53.0 - 59.3]	0.01
**'Legionella santicrucis' (GCF_001468135.1)**	**'Legionella sainthelensi' (GCF_900637685.1)**	53.1	[49.7 - 56.6]	36.6	[34.2 - 39.1]	49.1	[46.1 - 52.2]	0.41
**'Legionella anisa' (GCA_019930885.1)**	**'Legionella parisiensis' (GCF_900461585.1)**	54.9	[51.4 - 58.4]	36.5	[34.1 - 39.0]	50.5	[47.4 - 53.5]	0.31
**'Legionella cincinnatiensis' (GCF_900452415.1)**	**'Legionella sainthelensi' (GCF_900637685.1)**	56.9	[53.3 - 60.4]	36.5	[34.0 - 39.0]	52.0	[48.9 - 55.1]	0.19
**'Legionella longbeachae' (GCF_019930685.1)**	**'Legionella cincinnatiensis' (GCF_900452415.1)**	52.4	[48.9 - 55.8]	36.1	[33.7 - 38.6]	48.4	[45.3 - 51.4]	0.2
**'Legionella santicrucis' (GCF_001468135.1)**	**'Legionella longbeachae' (GCF_019930685.1)**	44.9	[41.5 - 48.3]	36.0	[33.6 - 38.5]	42.4	[39.4 - 45.4]	0.42
**'Legionella tucsonensis' (GCF_001468035.1)**	**'Legionella parisiensis' (GCF_900461585.1)**	52.0	[48.5 - 55.4]	35.8	[33.4 - 38.3]	47.9	[44.9 - 51.0]	0.56
**'Legionella resiliens' (GCF_021344005.1)**	**'Legionella parisiensis' (GCF_900461585.1)**	55.4	[51.9 - 58.9]	34.7	[32.3 - 37.2]	50.1	[47.1 - 53.2]	0.18
**'Legionella maioricensis' (GCF_023618015.1)**	**'Legionella steigerwaltii' (GCF_900452835.1)**	14.5	[11.7 - 17.9]	34.5	[32.1 - 37.0]	15.0	[12.5 - 17.8]	0.97
**'Legionella santicrucis' (GCF_001468135.1)**	**'Legionella gratiana' (GCF_900452545.1)**	40.8	[37.5 - 44.3]	34.3	[31.9 - 36.8]	38.6	[35.6 - 41.6]	0.32
**'Legionella cincinnatiensis' (GCF_900452415.1)**	**'Legionella gratiana' (GCF_900452545.1)**	44.7	[41.3 - 48.1]	33.1	[30.7 - 35.7]	41.4	[38.4 - 44.4]	0.11
**'Legionella sp. PATHC038' (GCF_026191355.1)**	**'Legionella steigerwaltii' (GCF_900452835.1)**	42.8	[39.4 - 46.2]	31.9	[29.5 - 34.4]	39.5	[36.5 - 42.5]	0.51
**'Legionella steigerwaltii' (GCF_900452835.1)**	**'Legionella cherrii' (GCF_900635815.1)**	46.5	[43.2 - 50.0]	31.2	[28.8 - 33.7]	42.1	[39.2 - 45.2]	0.58
**'Legionella saoudiensis' (GCF_001465875.1)**	**'Legionella maioricensis' (GCF_023618015.1)**	14.2	[11.4 - 17.6]	31.0	[28.6 - 33.5]	14.6	[12.2 - 17.5]	0.14
**'Legionella sp. PATHC039' (GCF_026191275.1)**	**'Legionella sp. PATHC038' (GCF_026191355.1)**	13.9	[11.1 - 17.2]	30.8	[28.4 - 33.3]	14.3	[11.9 - 17.1]	0.69
**'Legionella steelei' (GCF_001468005.1)**	**'Legionella steigerwaltii' (GCF_900452835.1)**	43.4	[40.1 - 46.9]	30.5	[28.1 - 33.0]	39.5	[36.6 - 42.6]	0.45

Query	Subject	d_0	C.I. d_0	d_4	C.I. d_4	d_6	C.I. d_6	Diff. G+C Percent
**'Legionella sp. PATHC035' (GCF_026191115.1)**	**'Legionella steigerwaltii' (GCF_900452835.1)**	45.4	[42.0 - 48.8]	30.4	[28.0 - 32.9]	40.9	[38.0 - 44.0]	1.12
**'Legionella anisa' (GCA_019930885.1)**	**'Legionella sp. PATHC039' (GCF_026191275.1)**	14.6	[11.8 - 18.0]	30.2	[27.8 - 32.7]	15.0	[12.5 - 17.9]	0.15
**'Legionella anisa' (GCA_019930885.1)**	**'Legionella sp. PATHC032' (GCF_026191185.1)**	14.2	[11.4 - 17.6]	30.2	[27.8 - 32.7]	14.6	[12.1 - 17.4]	0.25
**'Legionella fallonii LLAP-10' (GCF_000953135.1)**	**'Legionella steelei' (GCF_001468005.1)**	14.3	[11.4 - 17.6]	30.2	[27.8 - 32.7]	14.6	[12.2 - 17.5]	0.44
**'Legionella norrlandica' (GCF_000770585.1)**	**'Legionella septentrionalis' (GCF_003989745.1)**	13.3	[10.6 - 16.7]	30.2	[27.8 - 32.7]	13.7	[11.4 - 16.5]	4.69
**'Legionella gratiana' (GCF_900452545.1)**	**'Legionella sainthelensi' (GCF_900637685.1)**	36.7	[33.4 - 40.2]	30.0	[27.6 - 32.5]	34.2	[31.3 - 37.3]	0.08
**'Legionella longbeachae' (GCF_019930685.1)**	**'Legionella gratiana' (GCF_900452545.1)**	34.4	[31.0 - 37.9]	29.9	[27.5 - 32.4]	32.3	[29.4 - 35.4]	0.09
**'Legionella sp. PATHC039' (GCF_026191275.1)**	**'Legionella steigerwaltii' (GCF_900452835.1)**	14.1	[11.3 - 17.5]	29.7	[27.3 - 32.2]	14.5	[12.0 - 17.3]	0.18
**'Fluoribacter dumoffii NY 23' (GCF_000236165.1)**	**'Legionella rubrilucens' (GCF_900640015.1)**	13.3	[10.6 - 16.6]	29.6	[27.2 - 32.1]	13.7	[11.3 - 16.5]	7.9
**'Legionella saoudiensis' (GCF_001465875.1)**	**'Legionella steigerwaltii' (GCF_900452835.1)**	14.8	[11.9 - 18.2]	29.5	[27.1 - 32.0]	15.2	[12.7 - 18.0]	1.11
**'Legionella bononiensis' (GCF_016786415.1)**	**'Legionella steigerwaltii' (GCF_900452835.1)**	14.4	[11.6 - 17.8]	29.2	[26.8 - 31.7]	14.8	[12.3 - 17.6]	0.72
**'Legionella sp. PATHC039' (GCF_026191275.1)**	**'Legionella gratiana' (GCF_900452545.1)**	14.4	[11.6 - 17.8]	28.9	[26.6 - 31.4]	14.7	[12.3 - 17.6]	1.16
**'Legionella steelei' (GCF_001468005.1)**	**'Legionella sp. PATHC038' (GCF_026191355.1)**	31.7	[28.3 - 35.3]	28.9	[26.6 - 31.4]	30.0	[27.0 - 33.1]	0.06
**'Legionella gratiana' (GCF_900452545.1)**	**'Legionella parisiensis' (GCF_900461585.1)**	19.3	[16.2 - 22.9]	28.9	[26.6 - 31.4]	19.4	[16.7 - 22.4]	1.0
**'Legionella santicrucis' (GCF_001468135.1)**	**'Legionella rubrilucens' (GCF_900640015.1)**	13.2	[10.5 - 16.5]	28.8	[26.4 - 31.3]	13.6	[11.2 - 16.4]	10.88
**'Legionella sp. PATHC035' (GCF_026191115.1)**	**'Legionella sp. PATHC039' (GCF_026191275.1)**	13.9	[11.1 - 17.2]	28.8	[26.4 - 31.2]	14.2	[11.8 - 17.1]	1.3
**'Legionella pneumophila' (GCF_001941585.1)**	**'Legionella gratiana' (GCF_900452545.1)**	14.0	[11.2 - 17.3]	28.7	[26.3 - 31.2]	14.3	[11.9 - 17.2]	1.35
**'Legionella bononiensis' (GCF_016786415.1)**	**'Legionella moravica' (GCF_900452715.1)**	36.3	[32.9 - 39.8]	28.7	[26.3 - 31.2]	33.5	[30.6 - 36.6]	1.08
**'Legionella waltersii' (GCF_900187095.1)**	**'Legionella taurinensis' (GCF_900452865.1)**	13.3	[10.5 - 16.6]	28.6	[26.2 - 31.1]	13.6	[11.3 - 16.4]	8.88
**'Legionella steelei' (GCF_001468005.1)**	**'Legionella sp. PATHC035' (GCF_026191115.1)**	31.3	[27.9 - 34.9]	28.6	[26.2 - 31.1]	29.5	[26.6 - 32.6]	0.67
**'Fluoribacter dumoffii NY 23' (GCF_000236165.1)**	**'Legionella nagasakiensis' (GCF_900639915.1)**	13.1	[10.4 - 16.4]	28.4	[26.0 - 30.9]	13.5	[11.1 - 16.3]	1.46
**'Legionella bononiensis' (GCF_016786415.1)**	**'Legionella sp. PATHC039' (GCF_026191275.1)**	15.7	[12.8 - 19.2]	28.3	[26.0 - 30.8]	16.0	[13.5 - 18.9]	0.91
**'Legionella waltersii' (GCF_900187095.1)**	**'Legionella sainthelensi' (GCF_900637685.1)**	13.7	[11.0 - 17.1]	28.3	[25.9 - 30.8]	14.1	[11.7 - 16.9]	2.12
**'Legionella steelei' (GCF_001468005.1)**	**'Legionella cherrii' (GCF_900635815.1)**	34.5	[31.1 - 38.0]	28.3	[26.0 - 30.8]	32.0	[29.1 - 35.1]	0.13

Query	Subject	d_0	C.I. d_0	d_4	C.I. d_4	d_6	C.I. d_6	Diff. G+C Percent
**'Legionella antarctica' (GCF_011764505.1)**	**'Legionella maioricensis' (GCF_023618015.1)**	25.3	[21.9 - 28.9]	28.2	[25.8 - 30.7]	24.5	[21.7 - 27.6]	0.15
**'Legionella sp. PATHC039' (GCF_026191275.1)**	**'Legionella parisiensis' (GCF_900461585.1)**	14.3	[11.5 - 17.7]	28.2	[25.8 - 30.7]	14.7	[12.2 - 17.5]	0.16
**'Legionella quateirensis' (GCF_900452695.1)**	**'Legionella moravica' (GCF_900452715.1)**	35.8	[32.4 - 39.3]	28.1	[25.7 - 30.6]	33.0	[30.0 - 36.0]	1.06
**'Legionella anisa' (GCA_019930885.1)**	**'Fluoribacter gormanii' (GCF_900156395.1)**	30.1	[26.8 - 33.7]	28.0	[25.6 - 30.5]	28.5	[25.6 - 31.6]	0.2
**'Legionella pneumophila' (GCF_001941585.1)**	**'Legionella sp. PATHC038' (GCF_026191355.1)**	13.7	[10.9 - 17.0]	27.9	[25.6 - 30.4]	14.1	[11.7 - 16.9]	0.5
**'Legionella pneumophila' (GCF_001941585.1)**	**'Legionella parisiensis' (GCF_900461585.1)**	13.9	[11.1 - 17.3]	27.7	[25.3 - 30.2]	14.3	[11.9 - 17.1]	0.36
**'Legionella pneumophila' (GCF_001941585.1)**	**'Legionella septentrionalis' (GCF_003989745.1)**	13.3	[10.5 - 16.6]	27.6	[25.2 - 30.0]	13.7	[11.3 - 16.5]	3.83
**'Legionella qingyii' (GCF_003184185.1)**	**'Legionella bozemanae' (GCF_900640135.1)**	26.4	[23.1 - 30.1]	27.5	[25.2 - 30.0]	25.4	[22.6 - 28.5]	0.11
**'Legionella santicrucis' (GCF_001468135.1)**	**'Legionella parisiensis' (GCF_900461585.1)**	17.6	[14.5 - 21.1]	27.4	[25.0 - 29.9]	17.7	[15.1 - 20.7]	1.32
**'Legionella steigerwaltii' (GCF_900452835.1)**	**'Legionella rubrilucens' (GCF_900640015.1)**	13.1	[10.4 - 16.4]	27.4	[25.0 - 29.8]	13.5	[11.1 - 16.3]	9.22
**'Legionella anisa' (GCA_019930885.1)**	**'Legionella steigerwaltii' (GCF_900452835.1)**	27.7	[24.4 - 31.4]	27.3	[25.0 - 29.8]	26.5	[23.6 - 29.6]	0.04
**'Legionella norrlandica' (GCF_000770585.1)**	**'Legionella nagasakiensis' (GCF_900639915.1)**	13.3	[10.5 - 16.6]	27.3	[25.0 - 29.8]	13.7	[11.3 - 16.5]	3.62
**'Legionella anisa' (GCA_019930885.1)**	**'Legionella pneumophila' (GCF_001941585.1)**	14.2	[11.4 - 17.6]	27.2	[24.8 - 29.7]	14.5	[12.1 - 17.4]	0.04
**'Legionella drancourtii LLAP12' (GCF_000162755.2)**	**'Legionella santicrucis' (GCF_001468135.1)**	14.6	[11.8 - 18.0]	27.2	[24.8 - 29.7]	15.0	[12.5 - 17.8]	2.59
**'Legionella steigerwaltii' (GCF_900452835.1)**	**'Legionella bozemanae' (GCF_900640135.1)**	26.8	[23.4 - 30.4]	27.1	[24.7 - 29.6]	25.6	[22.7 - 28.7]	0.4
**'Legionella anisa' (GCA_019930885.1)**	**'Legionella gratiana' (GCF_900452545.1)**	18.8	[15.7 - 22.4]	27.1	[24.8 - 29.6]	18.8	[16.2 - 21.9]	1.31
**'Fluoribacter gormanii' (GCF_900156395.1)**	**'Legionella parisiensis' (GCF_900461585.1)**	29.0	[25.6 - 32.6]	27.1	[24.8 - 29.6]	27.4	[24.5 - 30.6]	0.11
**'Legionella anisa' (GCA_019930885.1)**	**'Legionella maioricensis' (GCF_023618015.1)**	14.1	[11.3 - 17.5]	27.1	[24.7 - 29.5]	14.5	[12.0 - 17.3]	1.0
**'Fluoribacter dumoffii NY 23' (GCF_000236165.1)**	**'Legionella santicrucis' (GCF_001468135.1)**	16.4	[13.4 - 19.9]	27.1	[24.7 - 29.6]	16.6	[14.1 - 19.6]	2.99
**'Legionella anisa' (GCA_019930885.1)**	**'Legionella santicrucis' (GCF_001468135.1)**	17.9	[14.8 - 21.4]	27.1	[24.7 - 29.6]	17.9	[15.3 - 20.9]	1.63
**'Legionella anisa' (GCA_019930885.1)**	**'Legionella qingyii' (GCF_003184185.1)**	25.3	[21.9 - 28.9]	27.1	[24.7 - 29.6]	24.4	[21.5 - 27.5]	0.48
**'Fluoribacter dumoffii NY 23' (GCF_000236165.1)**	**'Legionella quateirensis' (GCF_900452695.1)**	14.1	[11.3 - 17.5]	27.0	[24.6 - 29.4]	14.4	[12.0 - 17.3]	0.58
**'Legionella santicrucis' (GCF_001468135.1)**	**'Legionella quateirensis' (GCF_900452695.1)**	14.3	[11.4 - 17.6]	26.9	[24.6 - 29.4]	14.6	[12.1 - 17.4]	2.41
**'Legionella anisa' (GCA_019930885.1)**	**'Legionella sp. PATHC038' (GCF_026191355.1)**	24.7	[21.4 - 28.3]	26.9	[24.5 - 29.4]	23.9	[21.0 - 27.0]	0.54
**'Legionella cincinnatiensis' (GCF_900452415.1)**	**'Legionella quateirensis' (GCF_900452695.1)**	13.7	[10.9 - 17.1]	26.9	[24.5 - 29.4]	14.1	[11.7 - 16.9]	2.19

Query	Subject	d_0	C.I. d_0	d_4	C.I. d_4	d_6	C.I. d_6	Diff. G+C Percent
**'Legionella sp. PATHC032' (GCF_026191185.1)**	**'Legionella sp. PATHC038' (GCF_026191355.1)**	13.9	[11.1 - 17.2]	26.8	[24.4 - 29.3]	14.2	[11.8 - 17.0]	0.8
**'Legionella saoudiensis' (GCF_001465875.1)**	**'Legionella bononiensis' (GCF_016786415.1)**	13.9	[11.1 - 17.2]	26.8	[24.5 - 29.3]	14.2	[11.8 - 17.0]	0.38
**'Legionella qingyii' (GCF_003184185.1)**	**'Legionella resiliens' (GCF_021344005.1)**	24.9	[21.6 - 28.5]	26.8	[24.4 - 29.3]	24.0	[21.2 - 27.1]	0.35
**'Legionella tucsonensis' (GCF_001468035.1)**	**'Legionella qingyii' (GCF_003184185.1)**	27.5	[24.2 - 31.1]	26.8	[24.5 - 29.3]	26.2	[23.3 - 29.3]	0.39
**'Legionella bononiensis' (GCF_016786415.1)**	**'Legionella gratiana' (GCF_900452545.1)**	14.4	[11.5 - 17.8]	26.8	[24.4 - 29.2]	14.7	[12.2 - 17.6]	2.07
**'Fluoribacter gormanii' (GCF_900156395.1)**	**'Legionella gratiana' (GCF_900452545.1)**	18.2	[15.1 - 21.7]	26.7	[24.4 - 29.2]	18.2	[15.5 - 21.2]	1.11
**'Fluoribacter dumoffii NY 23' (GCF_000236165.1)**	**'Legionella gratiana' (GCF_900452545.1)**	17.2	[14.2 - 20.7]	26.7	[24.3 - 29.2]	17.3	[14.7 - 20.3]	2.66
**'Fluoribacter gormanii' (GCF_900156395.1)**	**'Legionella bozemanae' (GCF_900640135.1)**	28.9	[25.6 - 32.6]	26.7	[24.3 - 29.2]	27.3	[24.4 - 30.4]	0.17
**'Legionella resiliens' (GCF_021344005.1)**	**'Legionella rubrilucens' (GCF_900640015.1)**	13.1	[10.4 - 16.4]	26.7	[24.3 - 29.2]	13.5	[11.1 - 16.2]	9.38
**'Legionella bononiensis' (GCF_016786415.1)**	**'Legionella parisiensis' (GCF_900461585.1)**	14.4	[11.6 - 17.8]	26.5	[24.1 - 28.9]	14.8	[12.3 - 17.6]	1.07
**'Legionella saoudiensis' (GCF_001465875.1)**	**'Legionella quateirensis' (GCF_900452695.1)**	13.7	[10.9 - 17.1]	26.5	[24.2 - 29.0]	14.1	[11.7 - 16.9]	0.37
**'Legionella anisa' (GCA_019930885.1)**	**'Legionella quateirensis' (GCF_900452695.1)**	14.4	[11.6 - 17.8]	26.5	[24.2 - 29.0]	14.7	[12.3 - 17.6]	0.78
**'Legionella steigerwaltii' (GCF_900452835.1)**	**'Legionella parisiensis' (GCF_900461585.1)**	27.0	[23.6 - 30.6]	26.4	[24.1 - 28.9]	25.7	[22.8 - 28.8]	0.35
**'Legionella tucsonensis' (GCF_001468035.1)**	**'Fluoribacter gormanii' (GCF_900156395.1)**	30.0	[26.6 - 33.6]	26.4	[24.1 - 28.9]	28.1	[25.2 - 31.2]	0.67
**'Fluoribacter gormanii' (GCF_900156395.1)**	**'Legionella steigerwaltii' (GCF_900452835.1)**	27.9	[24.5 - 31.5]	26.4	[24.0 - 28.9]	26.4	[23.5 - 29.5]	0.24
**'Legionella resiliens' (GCF_021344005.1)**	**'Fluoribacter gormanii' (GCF_900156395.1)**	27.8	[24.4 - 31.4]	26.3	[23.9 - 28.7]	26.3	[23.4 - 29.4]	0.07
**'Legionella fallonii LLAP-10' (GCF_000953135.1)**	**'Legionella taurinensis' (GCF_900452865.1)**	13.2	[10.5 - 16.6]	26.3	[24.0 - 28.8]	13.6	[11.2 - 16.4]	9.73
**'Legionella qingyii' (GCF_003184185.1)**	**'Legionella parisiensis' (GCF_900461585.1)**	24.9	[21.6 - 28.5]	26.3	[23.9 - 28.7]	23.9	[21.1 - 27.0]	0.17
**'Legionella quateirensis' (GCF_900452695.1)**	**'Legionella steigerwaltii' (GCF_900452835.1)**	14.2	[11.4 - 17.6]	26.3	[23.9 - 28.8]	14.5	[12.1 - 17.3]	0.74
**'Fluoribacter dumoffii NY 23' (GCF_000236165.1)**	**'Legionella sp. PATHC039' (GCF_026191275.1)**	13.9	[11.1 - 17.3]	26.2	[23.9 - 28.7]	14.3	[11.8 - 17.1]	1.5
**'Legionella resiliens' (GCF_021344005.1)**	**'Legionella steigerwaltii' (GCF_900452835.1)**	25.7	[22.4 - 29.4]	26.2	[23.8 - 28.7]	24.6	[21.8 - 27.7]	0.16
**'Legionella steigerwaltii' (GCF_900452835.1)**	**'Legionella taurinensis' (GCF_900452865.1)**	13.1	[10.3 - 16.3]	26.2	[23.8 - 28.6]	13.4	[11.1 - 16.2]	9.74
**'Legionella anisa' (GCA_019930885.1)**	**'Legionella sp. PATHC035' (GCF_026191115.1)**	24.2	[20.9 - 27.9]	26.2	[23.9 - 28.7]	23.4	[20.6 - 26.5]	1.15
**'Legionella steelei' (GCF_001468005.1)**	**'Legionella maioricensis' (GCF_023618015.1)**	14.3	[11.5 - 17.7]	26.1	[23.8 - 28.6]	14.6	[12.2 - 17.5]	0.52
**'Legionella steelei' (GCF_001468005.1)**	**'Legionella bozemanae' (GCF_900640135.1)**	24.3	[21.0 - 28.0]	26.1	[23.7 - 28.6]	23.5	[20.6 - 26.6]	0.85
**'Legionella anisa' (GCA_019930885.1)**	**'Legionella cherrii' (GCF_900635815.1)**	24.2	[20.9 - 27.9]	26.1	[23.8 - 28.6]	23.4	[20.6 - 26.5]	0.62

Query	Subject	d_0	C.I. d_0	d_4	C.I. d_4	d_6	C.I. d_6	Diff. G+C Percent
**'Legionella anisa' (GCA_019930885.1)**	**'Legionella fallonii LLAP-10' (GCF_000953135.1)**	13.7	[10.9 - 17.0]	26.1	[23.8 - 28.6]	14.0	[11.6 - 16.9]	0.04
**'Legionella pneumophila' (GCF_001941585.1)**	**'Legionella sainthelensi' (GCF_900637685.1)**	13.4	[10.7 - 16.7]	26.1	[23.7 - 28.6]	13.8	[11.4 - 16.6]	1.27
**'Legionella tucsonensis' (GCF_001468035.1)**	**'Legionella steigerwaltii' (GCF_900452835.1)**	28.3	[24.9 - 31.9]	26.1	[23.8 - 28.6]	26.7	[23.8 - 29.8]	0.91
**'Legionella bononiensis' (GCF_016786415.1)**	**'Legionella sp. PATHC035' (GCF_026191115.1)**	14.1	[11.3 - 17.5]	26.1	[23.7 - 28.6]	14.5	[12.0 - 17.3]	0.39
**'Legionella norrlandica' (GCF_000770585.1)**	**'Legionella gratiana' (GCF_900452545.1)**	13.7	[10.9 - 17.1]	26.1	[23.8 - 28.6]	14.1	[11.7 - 16.9]	0.49
**'Legionella fallonii LLAP-10' (GCF_000953135.1)**	**'Legionella sp. PATHC038' (GCF_026191355.1)**	13.8	[11.0 - 17.1]	26.0	[23.6 - 28.4]	14.1	[11.7 - 17.0]	0.5
**'Legionella oakridgensis' (GCF_001467925.1)**	**'Legionella parisiensis' (GCF_900461585.1)**	12.9	[10.2 - 16.2]	26.0	[23.6 - 28.4]	13.3	[10.9 - 16.1]	2.86
**'Legionella qingyii' (GCF_003184185.1)**	**'Legionella steigerwaltii' (GCF_900452835.1)**	24.0	[20.7 - 27.7]	26.0	[23.7 - 28.5]	23.2	[20.4 - 26.3]	0.52
**'Legionella sp. PATHC039' (GCF_026191275.1)**	**'Fluoribacter gormanii' (GCF_900156395.1)**	13.8	[11.0 - 17.2]	25.9	[23.6 - 28.4]	14.1	[11.7 - 17.0]	0.05
**'Legionella septentrionalis' (GCF_003989745.1)**	**'Legionella parisiensis' (GCF_900461585.1)**	13.2	[10.5 - 16.5]	25.8	[23.5 - 28.3]	13.6	[11.2 - 16.4]	4.19
**'Legionella sp. PATHC038' (GCF_026191355.1)**	**'Legionella bozemanae' (GCF_900640135.1)**	23.5	[20.3 - 27.2]	25.8	[23.5 - 28.3]	22.8	[20.0 - 25.9]	0.91
**'Legionella santicrucis' (GCF_001468135.1)**	**'Legionella sp. PATHC039' (GCF_026191275.1)**	14.0	[11.2 - 17.4]	25.8	[23.4 - 28.3]	14.3	[11.9 - 17.2]	1.48
**'Legionella anisa' (GCA_019930885.1)**	**'Legionella steelei' (GCF_001468005.1)**	23.4	[20.1 - 27.1]	25.8	[23.4 - 28.3]	22.7	[19.9 - 25.8]	0.48
**'Fluoribacter dumoffii NY 23' (GCF_000236165.1)**	**'Legionella maioricensis' (GCF_023618015.1)**	13.8	[11.0 - 17.1]	25.6	[23.3 - 28.1]	14.1	[11.7 - 16.9]	0.35
**'Legionella saoudiensis' (GCF_001465875.1)**	**'Legionella santicrucis' (GCF_001468135.1)**	14.0	[11.2 - 17.3]	25.6	[23.3 - 28.1]	14.3	[11.9 - 17.1]	2.77
**'Legionella santicrucis' (GCF_001468135.1)**	**'Fluoribacter gormanii' (GCF_900156395.1)**	17.6	[14.5 - 21.1]	25.6	[23.2 - 28.0]	17.6	[15.0 - 20.6]	1.43
**'Legionella cherrii' (GCF_900635815.1)**	**'Legionella bozemanae' (GCF_900640135.1)**	24.1	[20.8 - 27.7]	25.6	[23.3 - 28.1]	23.2	[20.4 - 26.3]	0.98
**'Legionella sp. PATHC035' (GCF_026191115.1)**	**'Legionella quateirensis' (GCF_900452695.1)**	14.0	[11.2 - 17.4]	25.6	[23.2 - 28.1]	14.3	[11.9 - 17.2]	0.37
**'Legionella sp. PATHC035' (GCF_026191115.1)**	**'Legionella parisiensis' (GCF_900461585.1)**	22.7	[19.4 - 26.3]	25.6	[23.2 - 28.0]	22.0	[19.2 - 25.1]	1.46
**'Fluoribacter dumoffii NY 23' (GCF_000236165.1)**	**'Legionella steigerwaltii' (GCF_900452835.1)**	26.4	[23.0 - 30.0]	25.6	[23.3 - 28.1]	25.1	[22.2 - 28.2]	1.32
**'Legionella anisa' (GCA_019930885.1)**	**'Fluoribacter dumoffii NY 23' (GCF_000236165.1)**	23.6	[20.3 - 27.2]	25.6	[23.2 - 28.0]	22.8	[19.9 - 25.9]	1.36
**'Legionella gratiana' (GCF_900452545.1)**	**'Legionella steigerwaltii' (GCF_900452835.1)**	17.7	[14.6 - 21.2]	25.6	[23.2 - 28.0]	17.7	[15.1 - 20.7]	1.34
**'Legionella resiliens' (GCF_021344005.1)**	**'Legionella sp. PATHC038' (GCF_026191355.1)**	21.9	[18.7 - 25.6]	25.5	[23.2 - 28.0]	21.4	[18.6 - 24.5]	0.67
**'Legionella sp. PATHC038' (GCF_026191355.1)**	**'Legionella parisiensis' (GCF_900461585.1)**	21.6	[18.4 - 25.2]	25.5	[23.2 - 28.0]	21.1	[18.3 - 24.2]	0.85

Query	Subject	d_0	C.I. d_0	d_4	C.I. d_4	d_6	C.I. d_6	Diff. G+C Percent
**'Fluoribacter dumoffii NY 23' (GCF_000236165.1)**	**'Legionella steelei' (GCF_001468005.1)**	24.1	[20.8 - 27.7]	25.5	[23.2 - 28.0]	23.2	[20.4 - 26.3]	0.87
**'Legionella saoudiensis' (GCF_001465875.1)**	**'Legionella parisiensis' (GCF_900461585.1)**	14.1	[11.2 - 17.4]	25.5	[23.1 - 27.9]	14.4	[11.9 - 17.2]	1.45
**'Legionella sp. PATHC038' (GCF_026191355.1)**	**'Fluoribacter gormanii' (GCF_900156395.1)**	24.0	[20.7 - 27.7]	25.4	[23.0 - 27.8]	23.1	[20.3 - 26.2]	0.74
**'Legionella saoudiensis' (GCF_001465875.1)**	**'Legionella gratiana' (GCF_900452545.1)**	14.3	[11.5 - 17.7]	25.4	[23.1 - 27.9]	14.6	[12.1 - 17.4]	2.45
**'Legionella sp. PATHC038' (GCF_026191355.1)**	**'Legionella quateirensis' (GCF_900452695.1)**	13.8	[11.0 - 17.2]	25.4	[23.1 - 27.9]	14.2	[11.7 - 17.0]	0.24
**'Legionella sp. PATHC032' (GCF_026191185.1)**	**'Legionella sainthelensi' (GCF_900637685.1)**	13.4	[10.6 - 16.7]	25.4	[23.0 - 27.9]	13.7	[11.3 - 16.5]	0.97
**'Legionella tucsonensis' (GCF_001468035.1)**	**'Legionella cherrii' (GCF_900635815.1)**	24.9	[21.6 - 28.6]	25.4	[23.1 - 27.9]	23.9	[21.0 - 27.0]	1.49
**'Legionella resiliens' (GCF_021344005.1)**	**'Legionella cherrii' (GCF_900635815.1)**	23.2	[19.9 - 26.8]	25.4	[23.1 - 27.9]	22.4	[19.6 - 25.5]	0.74
**'Legionella steelei' (GCF_001468005.1)**	**'Legionella nagasakiensis' (GCF_900639915.1)**	13.1	[10.4 - 16.4]	25.4	[23.1 - 27.9]	13.5	[11.1 - 16.3]	2.33
**'Legionella qingyii' (GCF_003184185.1)**	**'Legionella cherrii' (GCF_900635815.1)**	22.6	[19.3 - 26.2]	25.4	[23.1 - 27.9]	22.0	[19.2 - 25.0]	1.1
**'Legionella qingyii' (GCF_003184185.1)**	**'Legionella sp. PATHC038' (GCF_026191355.1)**	21.8	[18.6 - 25.5]	25.3	[23.0 - 27.8]	21.3	[18.5 - 24.4]	1.02
**'Legionella nagasakiensis' (GCF_900639915.1)**	**'Legionella rubrilucens' (GCF_900640015.1)**	13.1	[10.4 - 16.4]	25.3	[23.0 - 27.8]	13.5	[11.1 - 16.3]	6.44
**'Legionella santicrucis' (GCF_001468135.1)**	**'Legionella sp. PATHC035' (GCF_026191115.1)**	15.9	[12.9 - 19.3]	25.3	[23.0 - 27.8]	16.0	[13.5 - 19.0]	2.78
**'Legionella santicrucis' (GCF_001468135.1)**	**'Legionella maioricensis' (GCF_023618015.1)**	13.8	[11.0 - 17.1]	25.3	[23.0 - 27.8]	14.1	[11.7 - 16.9]	2.63
**'Legionella anisa' (GCA_019930885.1)**	**'Legionella bononiensis' (GCF_016786415.1)**	14.5	[11.6 - 17.9]	25.3	[23.0 - 27.8]	14.8	[12.3 - 17.6]	0.76
**'Legionella maioricensis' (GCF_023618015.1)**	**'Legionella gratiana' (GCF_900452545.1)**	14.0	[11.2 - 17.3]	25.3	[22.9 - 27.7]	14.3	[11.9 - 17.1]	2.31
**'Legionella tucsonensis' (GCF_001468035.1)**	**'Legionella sp. PATHC038' (GCF_026191355.1)**	23.7	[20.4 - 27.3]	25.3	[23.0 - 27.8]	22.8	[20.0 - 25.9]	1.41
**'Legionella sp. PATHC035' (GCF_026191115.1)**	**'Legionella bozemanae' (GCF_900640135.1)**	22.9	[19.6 - 26.6]	25.3	[23.0 - 27.8]	22.2	[19.4 - 25.3]	1.52
**'Legionella sp. PATHC038' (GCF_026191355.1)**	**'Legionella worsleiensis' (GCF_900453045.1)**	13.8	[11.0 - 17.1]	25.2	[22.8 - 27.6]	14.1	[11.7 - 16.9]	1.59
**'Legionella saoudiensis' (GCF_001465875.1)**	**'Legionella sp. PATHC039' (GCF_026191275.1)**	13.5	[10.7 - 16.8]	25.2	[22.9 - 27.7]	13.8	[11.4 - 16.6]	1.29
**'Legionella quateirensis' (GCF_900452695.1)**	**'Legionella parisiensis' (GCF_900461585.1)**	14.3	[11.5 - 17.7]	25.2	[22.9 - 27.7]	14.6	[12.2 - 17.5]	1.09
**'Legionella drancourtii LLAP12' (GCF_000162755.2)**	**'Legionella waltersii' (GCF_900187095.1)**	13.7	[10.9 - 17.1]	25.2	[22.9 - 27.7]	14.1	[11.7 - 16.9]	0.07
**'Legionella fallonii LLAP-10' (GCF_000953135.1)**	**'Legionella worsleiensis' (GCF_900453045.1)**	14.1	[11.3 - 17.5]	25.2	[22.8 - 27.6]	14.4	[12.0 - 17.3]	2.09

Query	Subject	d_0	C.I. d_0	d_4	C.I. d_4	d_6	C.I. d_6	Diff. G+C Percent
**'Fluoribacter dumoffii NY 23' (GCF_000236165.1)**	**'Legionella parisiensis' (GCF_900461585.1)**	21.9	[18.6 - 25.5]	25.2	[22.8 - 27.7]	21.3	[18.5 - 24.4]	1.67
**'Legionella steelei' (GCF_001468005.1)**	**'Legionella resiliens' (GCF_021344005.1)**	23.1	[19.8 - 26.7]	25.1	[22.8 - 27.6]	22.3	[19.5 - 25.4]	0.61
**'Legionella sp. PATHC039' (GCF_026191275.1)**	**'Legionella wadsworthii' (GCF_900452925.1)**	13.8	[11.0 - 17.1]	25.1	[22.8 - 27.6]	14.1	[11.7 - 16.9]	0.05
**'Legionella sp. PATHC039' (GCF_026191275.1)**	**'Legionella bozemanae' (GCF_900640135.1)**	13.8	[11.0 - 17.1]	25.1	[22.8 - 27.6]	14.1	[11.7 - 17.0]	0.22
**'Legionella steelei' (GCF_001468005.1)**	**'Legionella parisiensis' (GCF_900461585.1)**	23.4	[20.1 - 27.0]	25.1	[22.7 - 27.5]	22.6	[19.7 - 25.6]	0.79
**'Legionella gratiana' (GCF_900452545.1)**	**'Legionella quateirensis' (GCF_900452695.1)**	14.3	[11.5 - 17.7]	25.1	[22.8 - 27.6]	14.6	[12.2 - 17.5]	2.08
**'Legionella parisiensis' (GCF_900461585.1)**	**'Legionella cherrii' (GCF_900635815.1)**	23.1	[19.8 - 26.7]	25.1	[22.8 - 27.6]	22.3	[19.5 - 25.4]	0.93
**'Legionella bononiensis' (GCF_016786415.1)**	**'Legionella bozemanae' (GCF_900640135.1)**	13.9	[11.1 - 17.3]	25.0	[22.7 - 27.5]	14.2	[11.8 - 17.1]	1.13
**'Legionella sp. PATHC035' (GCF_026191115.1)**	**'Fluoribacter gormanii' (GCF_900156395.1)**	24.2	[20.9 - 27.8]	25.0	[22.7 - 27.5]	23.2	[20.4 - 26.3]	1.35
**'Legionella santicrucis' (GCF_001468135.1)**	**'Legionella steigerwaltii' (GCF_900452835.1)**	16.5	[13.6 - 20.1]	25.0	[22.7 - 27.5]	16.7	[14.1 - 19.6]	1.67
**'Legionella saoudiensis' (GCF_001465875.1)**	**'Fluoribacter gormanii' (GCF_900156395.1)**	14.3	[11.5 - 17.7]	25.0	[22.7 - 27.5]	14.6	[12.2 - 17.5]	1.34
**'Legionella steelei' (GCF_001468005.1)**	**'Legionella tucsonensis' (GCF_001468035.1)**	24.0	[20.7 - 27.7]	25.0	[22.7 - 27.5]	23.1	[20.3 - 26.2]	1.36
**'Legionella bononiensis' (GCF_016786415.1)**	**'Fluoribacter gormanii' (GCF_900156395.1)**	14.0	[11.2 - 17.3]	25.0	[22.7 - 27.5]	14.3	[11.9 - 17.1]	0.96
**'Legionella fallonii LLAP-10' (GCF_000953135.1)**	**'Legionella bozemanae' (GCF_900640135.1)**	13.9	[11.2 - 17.3]	25.0	[22.7 - 27.5]	14.3	[11.8 - 17.1]	0.41
**'Legionella maioricensis' (GCF_023618015.1)**	**'Legionella bozemanae' (GCF_900640135.1)**	14.3	[11.4 - 17.6]	25.0	[22.7 - 27.5]	14.6	[12.1 - 17.4]	1.37
**'Legionella waltersii' (GCF_900187095.1)**	**'Legionella moravica' (GCF_900452715.1)**	14.6	[11.7 - 18.0]	25.0	[22.7 - 27.5]	14.9	[12.4 - 17.7]	0.95
**'Fluoribacter gormanii' (GCF_900156395.1)**	**'Legionella cherrii' (GCF_900635815.1)**	25.7	[22.3 - 29.3]	24.9	[22.6 - 27.4]	24.4	[21.5 - 27.5]	0.82
**'Legionella steelei' (GCF_001468005.1)**	**'Legionella qingyii' (GCF_003184185.1)**	22.5	[19.3 - 26.2]	24.9	[22.6 - 27.4]	21.8	[19.0 - 24.9]	0.96
**'Legionella anisa' (GCA_019930885.1)**	**'Legionella nagasakiensis' (GCF_900639915.1)**	13.1	[10.4 - 16.4]	24.9	[22.6 - 27.4]	13.5	[11.1 - 16.3]	2.81
**'Legionella resiliens' (GCF_021344005.1)**	**'Legionella sp. PATHC035' (GCF_026191115.1)**	22.0	[18.8 - 25.7]	24.9	[22.6 - 27.4]	21.4	[18.6 - 24.5]	1.28
**'Legionella anisa' (GCA_019930885.1)**	**'Legionella saoudiensis' (GCF_001465875.1)**	14.5	[11.6 - 17.8]	24.9	[22.5 - 27.3]	14.7	[12.3 - 17.6]	1.14
**'Legionella sp. PATHC039' (GCF_026191275.1)**	**'Legionella sainthelensi' (GCF_900637685.1)**	13.4	[10.7 - 16.8]	24.9	[22.6 - 27.4]	13.8	[11.4 - 16.6]	1.08
**'Legionella qingyii' (GCF_003184185.1)**	**'Legionella sp. PATHC035' (GCF_026191115.1)**	21.5	[18.3 - 25.2]	24.8	[22.5 - 27.3]	21.0	[18.2 - 24.0]	1.63
**'Legionella tucsonensis' (GCF_001468035.1)**	**'Legionella sp. PATHC035' (GCF_026191115.1)**	23.6	[20.3 - 27.3]	24.8	[22.5 - 27.3]	22.7	[19.9 - 25.8]	2.02

Query	Subject	d_0	C.I. d_0	d_4	C.I. d_4	d_6	C.I. d_6	Diff. G+C Percent
**'Legionella sp. PATHC039' (GCF_026191275.1)**	**'Legionella rubrilucens' (GCF_900640015.1)**	13.2	[10.5 - 16.5]	24.8	[22.5 - 27.3]	13.6	[11.2 - 16.4]	9.4
**'Fluoribacter dumoffii NY 23' (GCF_000236165.1)**	**'Legionella sp. PATHC035' (GCF_026191115.1)**	23.8	[20.5 - 27.5]	24.8	[22.4 - 27.2]	22.9	[20.1 - 26.0]	0.2
**'Legionella maioricensis' (GCF_023618015.1)**	**'Legionella parisiensis' (GCF_900461585.1)**	14.1	[11.3 - 17.4]	24.7	[22.4 - 27.2]	14.4	[11.9 - 17.2]	1.31
**'Legionella gratiana' (GCF_900452545.1)**	**'Legionella bozemanae' (GCF_900640135.1)**	17.5	[14.5 - 21.1]	24.7	[22.4 - 27.2]	17.5	[14.9 - 20.5]	0.94
**'Legionella septentrionalis' (GCF_003989745.1)**	**'Legionella gratiana' (GCF_900452545.1)**	13.1	[10.4 - 16.4]	24.7	[22.4 - 27.2]	13.5	[11.1 - 16.3]	5.18
**'Fluoribacter dumoffii NY 23' (GCF_000236165.1)**	**'Legionella bononiensis' (GCF_016786415.1)**	14.2	[11.4 - 17.6]	24.7	[22.4 - 27.2]	14.5	[12.0 - 17.3]	0.6
**'Legionella drancourtii LLAP12' (GCF_000162755.2)**	**'Legionella parisiensis' (GCF_900461585.1)**	14.5	[11.7 - 17.9]	24.7	[22.3 - 27.1]	14.8	[12.3 - 17.7]	1.27
**'Legionella anisa' (GCA_019930885.1)**	**'Legionella cincinnatiensis' (GCF_900452415.1)**	17.3	[14.3 - 20.8]	24.7	[22.4 - 27.2]	17.3	[14.7 - 20.3]	1.42
**'Legionella maioricensis' (GCF_023618015.1)**	**'Fluoribacter gormanii' (GCF_900156395.1)**	13.9	[11.1 - 17.3]	24.7	[22.4 - 27.1]	14.2	[11.8 - 17.1]	1.2
**'Legionella anisa' (GCA_019930885.1)**	**'Legionella wadsworthii' (GCF_900452925.1)**	20.4	[17.2 - 24.0]	24.6	[22.3 - 27.0]	20.0	[17.3 - 23.1]	0.2
**'Legionella steelei' (GCF_001468005.1)**	**'Fluoribacter gormanii' (GCF_900156395.1)**	24.2	[20.9 - 27.9]	24.6	[22.3 - 27.1]	23.2	[20.4 - 26.3]	0.68
**'Fluoribacter gormanii' (GCF_900156395.1)**	**'Legionella quateirensis' (GCF_900452695.1)**	14.2	[11.4 - 17.6]	24.6	[22.3 - 27.1]	14.5	[12.1 - 17.4]	0.98
**'Legionella santicrucis' (GCF_001468135.1)**	**'Legionella bononiensis' (GCF_016786415.1)**	14.0	[11.2 - 17.4]	24.6	[22.3 - 27.1]	14.3	[11.9 - 17.2]	2.39
**'Fluoribacter dumoffii NY 23' (GCF_000236165.1)**	**'Legionella sp. PATHC038' (GCF_026191355.1)**	23.5	[20.2 - 27.2]	24.6	[22.3 - 27.1]	22.6	[19.8 - 25.7]	0.81
**'Legionella pneumophila' (GCF_001941585.1)**	**'Legionella steigerwaltii' (GCF_900452835.1)**	13.6	[10.9 - 17.0]	24.6	[22.2 - 27.0]	14.0	[11.6 - 16.8]	0.01
**'Legionella sp. PATHC035' (GCF_026191115.1)**	**'Legionella gratiana' (GCF_900452545.1)**	16.5	[13.5 - 20.0]	24.6	[22.3 - 27.1]	16.6	[14.0 - 19.5]	2.46
**'Legionella fallonii LLAP-10' (GCF_000953135.1)**	**'Legionella steigerwaltii' (GCF_900452835.1)**	13.7	[10.9 - 17.1]	24.6	[22.3 - 27.1]	14.0	[11.6 - 16.9]	0.01
**'Legionella sp. PATHC039' (GCF_026191275.1)**	**'Legionella taurinensis' (GCF_900452865.1)**	13.2	[10.5 - 16.5]	24.5	[22.2 - 27.0]	13.6	[11.2 - 16.4]	9.92
**'Legionella oakridgensis' (GCF_001467925.1)**	**'Legionella nagasakiensis' (GCF_900639915.1)**	30.0	[26.6 - 33.6]	24.5	[22.2 - 26.9]	27.6	[24.7 - 30.7]	0.26
**'Legionella cincinnatiensis' (GCF_900452415.1)**	**'Legionella steigerwaltii' (GCF_900452835.1)**	16.9	[13.8 - 20.4]	24.5	[22.2 - 27.0]	16.9	[14.3 - 19.9]	1.45
**'Legionella sp. PATHC035' (GCF_026191115.1)**	**'Legionella nagasakiensis' (GCF_900639915.1)**	12.7	[10.1 - 16.0]	24.5	[22.2 - 27.0]	13.1	[10.8 - 15.9]	1.66
**'Legionella anisa' (GCA_019930885.1)**	**'Legionella worsleiensis' (GCF_900453045.1)**	13.7	[11.0 - 17.1]	24.5	[22.2 - 27.0]	14.1	[11.7 - 16.9]	2.13
**'Legionella steelei' (GCF_001468005.1)**	**'Legionella rubrilucens' (GCF_900640015.1)**	13.0	[10.3 - 16.3]	24.5	[22.2 - 27.0]	13.4	[11.0 - 16.1]	8.77
**'Fluoribacter dumoffii NY 23' (GCF_000236165.1)**	**'Legionella cherrii' (GCF_900635815.1)**	25.1	[21.8 - 28.8]	24.5	[22.1 - 26.9]	23.9	[21.0 - 27.0]	0.74

Query	Subject	d_0	C.I. d_0	d_4	C.I. d_4	d_6	C.I. d_6	Diff. G+C Percent
**'Legionella taurinensis' (GCF_900452865.1)**	**'Legionella parisiensis' (GCF_900461585.1)**	13.1	[10.4 - 16.4]	24.4	[22.1 - 26.8]	13.5	[11.1 - 16.3]	10.08
**'Legionella sp. PATHC038' (GCF_026191355.1)**	**'Legionella cincinnatiensis' (GCF_900452415.1)**	16.1	[13.2 - 19.6]	24.4	[22.1 - 26.9]	16.3	[13.7 - 19.2]	1.96
**'Fluoribacter dumoffii NY 23' (GCF_000236165.1)**	**'Fluoribacter gormanii' (GCF_900156395.1)**	23.5	[20.2 - 27.1]	24.4	[22.1 - 26.8]	22.5	[19.7 - 25.6]	1.56
**'Legionella santicrucis' (GCF_001468135.1)**	**'Legionella resiliens' (GCF_021344005.1)**	16.2	[13.3 - 19.7]	24.4	[22.1 - 26.8]	16.4	[13.8 - 19.3]	1.5
**'Legionella sainthelensi' (GCF_900637685.1)**	**'Legionella rubrilucens' (GCF_900640015.1)**	13.0	[10.3 - 16.3]	24.4	[22.1 - 26.9]	13.4	[11.0 - 16.1]	10.48
**'Legionella fallonii LLAP-10' (GCF_000953135.1)**	**'Legionella septentrionalis' (GCF_003989745.1)**	12.7	[10.0 - 15.9]	24.3	[22.0 - 26.8]	13.1	[10.7 - 15.8]	3.83
**'Legionella maioricensis' (GCF_023618015.1)**	**'Legionella sp. PATHC035' (GCF_026191115.1)**	13.9	[11.2 - 17.3]	24.3	[22.0 - 26.8]	14.3	[11.8 - 17.1]	0.15
**'Fluoribacter dumoffii NY 23' (GCF_000236165.1)**	**'Legionella bozemanae' (GCF_900640135.1)**	21.9	[18.7 - 25.6]	24.3	[22.0 - 26.8]	21.3	[18.5 - 24.3]	1.72
**'Legionella parisiensis' (GCF_900461585.1)**	**'Legionella rubrilucens' (GCF_900640015.1)**	13.2	[10.5 - 16.5]	24.3	[22.0 - 26.8]	13.6	[11.2 - 16.4]	9.56
**'Legionella drancourtii LLAP12' (GCF_000162755.2)**	**'Legionella fallonii LLAP-10' (GCF_000953135.1)**	14.1	[11.3 - 17.5]	24.3	[22.0 - 26.8]	14.4	[12.0 - 17.3]	0.92
**'Legionella norrlandica' (GCF_000770585.1)**	**'Legionella parisiensis' (GCF_900461585.1)**	13.7	[10.9 - 17.0]	24.3	[22.0 - 26.8]	14.0	[11.6 - 16.8]	0.5
**'Legionella gratiana' (GCF_900452545.1)**	**'Legionella rubrilucens' (GCF_900640015.1)**	13.3	[10.5 - 16.6]	24.3	[22.0 - 26.8]	13.6	[11.3 - 16.4]	10.56
**'Legionella santicrucis' (GCF_001468135.1)**	**'Legionella sp. PATHC038' (GCF_026191355.1)**	16.0	[13.1 - 19.5]	24.2	[21.9 - 26.7]	16.2	[13.6 - 19.1]	2.17
**'Legionella quateirensis' (GCF_900452695.1)**	**'Legionella wadsworthii' (GCF_900452925.1)**	13.9	[11.1 - 17.3]	24.2	[21.9 - 26.7]	14.2	[11.8 - 17.1]	0.98
**'Legionella drancourtii LLAP12' (GCF_000162755.2)**	**'Legionella taurinensis' (GCF_900452865.1)**	13.1	[10.4 - 16.4]	24.2	[21.9 - 26.7]	13.5	[11.1 - 16.2]	8.81
**'Legionella bononiensis' (GCF_016786415.1)**	**'Legionella cincinnatiensis' (GCF_900452415.1)**	13.8	[11.0 - 17.1]	24.1	[21.8 - 26.6]	14.1	[11.7 - 16.9]	2.18
**'Fluoribacter gormanii' (GCF_900156395.1)**	**'Legionella cincinnatiensis' (GCF_900452415.1)**	16.7	[13.7 - 20.2]	24.1	[21.8 - 26.6]	16.7	[14.1 - 19.7]	1.22
**'Fluoribacter dumoffii NY 23' (GCF_000236165.1)**	**'Legionella pneumophila' (GCF_001941585.1)**	13.6	[10.9 - 17.0]	24.1	[21.7 - 26.5]	14.0	[11.6 - 16.8]	1.31
**'Legionella sp. PATHC039' (GCF_026191275.1)**	**'Legionella cincinnatiensis' (GCF_900452415.1)**	13.8	[11.0 - 17.2]	24.1	[21.8 - 26.6]	14.1	[11.7 - 17.0]	1.27
**'Legionella sp. PATHC035' (GCF_026191115.1)**	**'Legionella cincinnatiensis' (GCF_900452415.1)**	15.9	[12.9 - 19.4]	24.1	[21.8 - 26.6]	16.0	[13.5 - 19.0]	2.57
**'Legionella longbeachae' (GCF_019930685.1)**	**'Legionella sp. PATHC039' (GCF_026191275.1)**	13.3	[10.6 - 16.7]	24.1	[21.8 - 26.6]	13.7	[11.3 - 16.5]	1.07
**'Legionella bononiensis' (GCF_016786415.1)**	**'Legionella sainthelensi' (GCF_900637685.1)**	13.6	[10.8 - 16.9]	24.1	[21.8 - 26.6]	13.9	[11.5 - 16.7]	1.98
**'Legionella steelei' (GCF_001468005.1)**	**'Legionella waltersii' (GCF_900187095.1)**	13.6	[10.8 - 16.9]	24.0	[21.7 - 26.5]	13.9	[11.5 - 16.7]	0.41
**'Fluoribacter dumoffii NY 23' (GCF_000236165.1)**	**'Legionella oakridgensis' (GCF_001467925.1)**	13.0	[10.3 - 16.3]	24.0	[21.7 - 26.5]	13.4	[11.0 - 16.1]	1.19

Query	Subject	d_0	C.I. d_0	d_4	C.I. d_4	d_6	C.I. d_6	Diff. G+C Percent
**'Legionella shakespearei DSM 23087' (GCF_000373765.1)**	**'Legionella sainthelensi' (GCF_900637685.1)**	13.4	[10.7 - 16.7]	24.0	[21.7 - 26.4]	13.8	[11.4 - 16.6]	4.64
**'Legionella fallonii LLAP-10' (GCF_000953135.1)**	**'Legionella maioricensis' (GCF_023618015.1)**	17.4	[14.3 - 20.9]	24.0	[21.7 - 26.5]	17.4	[14.7 - 20.3]	0.96
**'Legionella waltersii' (GCF_900187095.1)**	**'Legionella cincinnatiensis' (GCF_900452415.1)**	13.5	[10.7 - 16.8]	24.0	[21.6 - 26.4]	13.8	[11.4 - 16.6]	2.31
**'Legionella septentrionalis' (GCF_003989745.1)**	**'Legionella nagasakiensis' (GCF_900639915.1)**	13.8	[11.0 - 17.1]	24.0	[21.7 - 26.5]	14.1	[11.7 - 16.9]	1.06
**'Legionella norrlandica' (GCF_000770585.1)**	**'Legionella pneumophila' (GCF_001941585.1)**	20.8	[17.6 - 24.4]	24.0	[21.7 - 26.5]	20.3	[17.5 - 23.3]	0.86
**'Legionella gratiana' (GCF_900452545.1)**	**'Legionella wadsworthii' (GCF_900452925.1)**	16.1	[13.1 - 19.5]	23.9	[21.6 - 26.4]	16.2	[13.6 - 19.1]	1.11
**'Legionella anisa' (GCA_019930885.1)**	**'Legionella drancourtii LLAP12' (GCF_000162755.2)**	14.7	[11.8 - 18.1]	23.9	[21.6 - 26.4]	14.9	[12.5 - 17.8]	0.96
**'Fluoribacter dumoffii NY 23' (GCF_000236165.1)**	**'Legionella sp. PATHC032' (GCF_026191185.1)**	13.6	[10.8 - 16.9]	23.8	[21.5 - 26.2]	13.9	[11.5 - 16.7]	1.61
**'Legionella anisa' (GCA_019930885.1)**	**'Legionella longbeachae' (GCF_019930685.1)**	16.1	[13.2 - 19.6]	23.8	[21.5 - 26.3]	16.3	[13.7 - 19.2]	1.21
**'Legionella steigerwaltii' (GCF_900452835.1)**	**'Legionella nagasakiensis' (GCF_900639915.1)**	12.9	[10.2 - 16.2]	23.8	[21.5 - 26.3]	13.3	[10.9 - 16.1]	2.78
**'Legionella pneumophila' (GCF_001941585.1)**	**'Legionella rubrilucens' (GCF_900640015.1)**	13.3	[10.6 - 16.7]	23.8	[21.5 - 26.2]	13.7	[11.3 - 16.5]	9.21
**'Legionella wadsworthii' (GCF_900452925.1)**	**'Legionella parisiensis' (GCF_900461585.1)**	19.6	[16.4 - 23.2]	23.8	[21.5 - 26.2]	19.2	[16.5 - 22.3]	0.11
**'Legionella maioricensis' (GCF_023618015.1)**	**'Legionella wadsworthii' (GCF_900452925.1)**	13.7	[11.0 - 17.1]	23.8	[21.5 - 26.2]	14.1	[11.7 - 16.9]	1.2
**'Legionella santicrucis' (GCF_001468135.1)**	**'Legionella bozemanae' (GCF_900640135.1)**	16.5	[13.5 - 20.0]	23.8	[21.5 - 26.2]	16.6	[14.0 - 19.5]	1.26
**'Legionella saoudiensis' (GCF_001465875.1)**	**'Legionella sp. PATHC035' (GCF_026191115.1)**	14.5	[11.6 - 17.9]	23.8	[21.5 - 26.2]	14.8	[12.3 - 17.6]	0.01
**'Legionella fallonii LLAP-10' (GCF_000953135.1)**	**'Legionella waltersii' (GCF_900187095.1)**	14.0	[11.2 - 17.4]	23.8	[21.5 - 26.3]	14.3	[11.9 - 17.1]	0.85
**'Legionella steelei' (GCF_001468005.1)**	**'Legionella bononiensis' (GCF_016786415.1)**	14.0	[11.2 - 17.4]	23.8	[21.5 - 26.3]	14.3	[11.9 - 17.2]	0.28
**'Legionella steigerwaltii' (GCF_900452835.1)**	**'Legionella wadsworthii' (GCF_900452925.1)**	18.8	[15.6 - 22.3]	23.7	[21.4 - 26.2]	18.5	[15.9 - 21.5]	0.24
**'Legionella sp. PATHC038' (GCF_026191355.1)**	**'Legionella gratiana' (GCF_900452545.1)**	16.4	[13.4 - 19.9]	23.7	[21.4 - 26.1]	16.4	[13.9 - 19.4]	1.85
**'Legionella bononiensis' (GCF_016786415.1)**	**'Legionella sp. PATHC038' (GCF_026191355.1)**	13.9	[11.1 - 17.2]	23.7	[21.4 - 26.2]	14.2	[11.8 - 17.0]	0.22
**'Legionella parisiensis' (GCF_900461585.1)**	**'Legionella nagasakiensis' (GCF_900639915.1)**	13.2	[10.5 - 16.5]	23.7	[21.4 - 26.2]	13.5	[11.2 - 16.3]	3.12
**'Legionella waltersii' (GCF_900187095.1)**	**'Legionella gratiana' (GCF_900452545.1)**	13.7	[10.9 - 17.0]	23.7	[21.4 - 26.1]	14.0	[11.6 - 16.8]	2.2
**'Legionella cincinnatiensis' (GCF_900452415.1)**	**'Legionella parisiensis' (GCF_900461585.1)**	17.3	[14.3 - 20.9]	23.7	[21.4 - 26.1]	17.3	[14.7 - 20.3]	1.11

Query	Subject	d_0	C.I. d_0	d_4	C.I. d_4	d_6	C.I. d_6	Diff. G+C Percent
**'Legionella maioricensis' (GCF_023618015.1)**	**'Legionella sp. PATHC039' (GCF_026191275.1)**	15.0	[12.1 - 18.4]	23.6	[21.3 - 26.1]	15.2	[12.7 - 18.1]	1.15
**'Legionella sp. PATHC035' (GCF_026191115.1)**	**'Legionella wadsworthii' (GCF_900452925.1)**	17.8	[14.7 - 21.4]	23.6	[21.3 - 26.1]	17.7	[15.1 - 20.7]	1.35
**'Legionella tucsonensis' (GCF_001468035.1)**	**'Legionella cincinnatiensis' (GCF_900452415.1)**	17.1	[14.1 - 20.7]	23.6	[21.3 - 26.1]	17.1	[14.5 - 20.1]	0.54
**'Legionella quateirensis' (GCF_900452695.1)**	**'Legionella sainthelensi' (GCF_900637685.1)**	13.5	[10.8 - 16.9]	23.5	[21.2 - 26.0]	13.9	[11.5 - 16.7]	2.0
**'Legionella septentrionalis' (GCF_003989745.1)**	**'Legionella sainthelensi' (GCF_900637685.1)**	12.9	[10.2 - 16.2]	23.5	[21.2 - 26.0]	13.3	[11.0 - 16.1]	5.1
**'Legionella norrlandica' (GCF_000770585.1)**	**'Legionella sp. PATHC039' (GCF_026191275.1)**	20.1	[16.9 - 23.7]	23.5	[21.2 - 26.0]	19.7	[16.9 - 22.7]	0.67
**'Legionella saoudiensis' (GCF_001465875.1)**	**'Legionella bozemanae' (GCF_900640135.1)**	14.2	[11.4 - 17.6]	23.5	[21.2 - 26.0]	14.5	[12.1 - 17.3]	1.51
**'Legionella cincinnatiensis' (GCF_900452415.1)**	**'Legionella bozemanae' (GCF_900640135.1)**	16.9	[13.9 - 20.4]	23.5	[21.2 - 26.0]	16.9	[14.3 - 19.8]	1.05
**'Legionella steelei' (GCF_001468005.1)**	**'Legionella cincinnatiensis' (GCF_900452415.1)**	16.3	[13.3 - 19.8]	23.5	[21.2 - 26.0]	16.4	[13.8 - 19.3]	1.9
**'Legionella cincinnatiensis' (GCF_900452415.1)**	**'Legionella cherrii' (GCF_900635815.1)**	15.8	[12.8 - 19.2]	23.5	[21.2 - 26.0]	15.9	[13.4 - 18.8]	2.03
**'Legionella sp. PATHC032' (GCF_026191185.1)**	**'Legionella steigerwaltii' (GCF_900452835.1)**	13.4	[10.7 - 16.8]	23.5	[21.2 - 26.0]	13.8	[11.4 - 16.6]	0.29
**'Legionella moravica' (GCF_900452715.1)**	**'Legionella parisiensis' (GCF_900461585.1)**	13.7	[11.0 - 17.1]	23.5	[21.2 - 25.9]	14.1	[11.7 - 16.9]	2.15
**'Legionella tucsonensis' (GCF_001468035.1)**	**'Legionella gratiana' (GCF_900452545.1)**	17.7	[14.6 - 21.2]	23.5	[21.2 - 26.0]	17.6	[15.0 - 20.6]	0.43
**'Legionella gratiana' (GCF_900452545.1)**	**'Legionella taurinensis' (GCF_900452865.1)**	13.2	[10.5 - 16.5]	23.5	[21.2 - 25.9]	13.6	[11.2 - 16.3]	11.08
**'Legionella norrlandica' (GCF_000770585.1)**	**'Legionella sainthelensi' (GCF_900637685.1)**	13.5	[10.8 - 16.9]	23.5	[21.2 - 26.0]	13.9	[11.5 - 16.7]	0.41
**'Legionella santicrucis' (GCF_001468135.1)**	**'Legionella cherrii' (GCF_900635815.1)**	15.7	[12.8 - 19.2]	23.4	[21.1 - 25.9]	15.9	[13.3 - 18.8]	2.25
**'Legionella gratiana' (GCF_900452545.1)**	**'Legionella cherrii' (GCF_900635815.1)**	16.7	[13.7 - 20.2]	23.4	[21.1 - 25.9]	16.7	[14.1 - 19.7]	1.92
**'Legionella resiliens' (GCF_021344005.1)**	**'Legionella gratiana' (GCF_900452545.1)**	17.4	[14.4 - 21.0]	23.4	[21.1 - 25.9]	17.4	[14.8 - 20.3]	1.18
**'Fluoribacter dumoffii NY 23' (GCF_000236165.1)**	**'Legionella resiliens' (GCF_021344005.1)**	20.7	[17.5 - 24.3]	23.4	[21.1 - 25.8]	20.1	[17.4 - 23.2]	1.48
**'Legionella drancourtii LLAP12' (GCF_000162755.2)**	**'Legionella bononiensis' (GCF_016786415.1)**	13.9	[11.1 - 17.2]	23.4	[21.1 - 25.9]	14.2	[11.8 - 17.0]	0.2
**'Legionella saoudiensis' (GCF_001465875.1)**	**'Legionella qingyii' (GCF_003184185.1)**	14.1	[11.3 - 17.5]	23.4	[21.1 - 25.9]	14.4	[12.0 - 17.2]	1.62
**'Legionella pneumophila' (GCF_001941585.1)**	**'Legionella bozemanae' (GCF_900640135.1)**	13.6	[10.9 - 17.0]	23.4	[21.1 - 25.8]	14.0	[11.6 - 16.8]	0.41
**'Legionella sp. PATHC032' (GCF_026191185.1)**	**'Legionella cincinnatiensis' (GCF_900452415.1)**	13.5	[10.7 - 16.8]	23.4	[21.1 - 25.8]	13.8	[11.4 - 16.6]	1.16
**'Legionella pneumophila' (GCF_001941585.1)**	**'Legionella longbeachae' (GCF_019930685.1)**	13.4	[10.6 - 16.7]	23.4	[21.1 - 25.9]	13.7	[11.3 - 16.5]	1.26

Query	Subject	d_0	C.I. d_0	d_4	C.I. d_4	d_6	C.I. d_6	Diff. G+C Percent
**'Legionella pneumophila' (GCF_001941585.1)**	**'Legionella taurinensis' (GCF_900452865.1)**	13.4	[10.6 - 16.7]	23.4	[21.1 - 25.9]	13.7	[11.3 - 16.5]	9.73
**'Legionella longbeachae' (GCF_019930685.1)**	**'Legionella moravica' (GCF_900452715.1)**	13.5	[10.7 - 16.8]	23.4	[21.1 - 25.9]	13.8	[11.4 - 16.6]	3.06
**'Legionella longbeachae' (GCF_019930685.1)**	**'Legionella parisiensis' (GCF_900461585.1)**	16.3	[13.3 - 19.8]	23.3	[21.1 - 25.8]	16.4	[13.8 - 19.3]	0.9
**'Legionella qingyii' (GCF_003184185.1)**	**'Legionella gratiana' (GCF_900452545.1)**	16.7	[13.7 - 20.2]	23.3	[21.0 - 25.8]	16.7	[14.1 - 19.7]	0.83
**'Legionella bononiensis' (GCF_016786415.1)**	**'Legionella wadsworthii' (GCF_900452925.1)**	13.8	[11.0 - 17.1]	23.3	[21.0 - 25.7]	14.1	[11.7 - 16.9]	0.96
**'Legionella waltersii' (GCF_900187095.1)**	**'Legionella steigerwaltii' (GCF_900452835.1)**	13.4	[10.7 - 16.7]	23.3	[21.1 - 25.8]	13.8	[11.4 - 16.6]	0.86
**'Legionella santicrucis' (GCF_001468135.1)**	**'Legionella qingyii' (GCF_003184185.1)**	15.9	[12.9 - 19.4]	23.3	[21.0 - 25.7]	16.0	[13.5 - 18.9]	1.15
**'Legionella bononiensis' (GCF_016786415.1)**	**'Legionella waltersii' (GCF_900187095.1)**	14.7	[11.8 - 18.1]	23.3	[21.0 - 25.8]	14.9	[12.5 - 17.8]	0.13
**'Legionella parisiensis' (GCF_900461585.1)**	**'Legionella sainthelensi' (GCF_900637685.1)**	16.5	[13.5 - 20.0]	23.3	[21.0 - 25.7]	16.5	[13.9 - 19.4]	0.91
**'Legionella resiliens' (GCF_021344005.1)**	**'Legionella sainthelensi' (GCF_900637685.1)**	15.9	[12.9 - 19.3]	23.2	[20.9 - 25.6]	16.0	[13.4 - 18.9]	1.1
**'Legionella steigerwaltii' (GCF_900452835.1)**	**'Legionella sainthelensi' (GCF_900637685.1)**	16.4	[13.4 - 19.9]	23.2	[20.9 - 25.6]	16.4	[13.9 - 19.4]	1.26
**'Legionella resiliens' (GCF_021344005.1)**	**'Legionella cincinnatiensis' (GCF_900452415.1)**	16.6	[13.6 - 20.1]	23.2	[20.9 - 25.7]	16.6	[14.1 - 19.6]	1.29
**'Legionella maioricensis' (GCF_023618015.1)**	**'Legionella sp. PATHC038' (GCF_026191355.1)**	13.9	[11.1 - 17.3]	23.2	[20.9 - 25.6]	14.2	[11.8 - 17.1]	0.46
**'Legionella fallonii LLAP-10' (GCF_000953135.1)**	**'Legionella saoudiensis' (GCF_001465875.1)**	13.6	[10.8 - 16.9]	23.2	[20.9 - 25.7]	13.9	[11.5 - 16.7]	1.1
**'Legionella sp. PATHC032' (GCF_026191185.1)**	**'Legionella gratiana' (GCF_900452545.1)**	13.6	[10.8 - 16.9]	23.2	[20.9 - 25.6]	13.9	[11.5 - 16.7]	1.05
**'Legionella tucsonensis' (GCF_001468035.1)**	**'Legionella santicrucis' (GCF_001468135.1)**	16.3	[13.3 - 19.8]	23.2	[20.9 - 25.7]	16.4	[13.8 - 19.3]	0.76
**'Legionella saoudiensis' (GCF_001465875.1)**	**'Legionella cincinnatiensis' (GCF_900452415.1)**	13.8	[11.0 - 17.2]	23.2	[20.9 - 25.6]	14.1	[11.7 - 16.9]	2.56
**'Legionella longbeachae' (GCF_019930685.1)**	**'Legionella resiliens' (GCF_021344005.1)**	15.5	[12.6 - 19.0]	23.2	[20.9 - 25.7]	15.7	[13.2 - 18.6]	1.09
**'Fluoribacter dumoffii NY 23' (GCF_000236165.1)**	**'Legionella saoudiensis' (GCF_001465875.1)**	14.3	[11.5 - 17.7]	23.2	[20.9 - 25.6]	14.6	[12.2 - 17.5]	0.21
**'Legionella oakridgensis' (GCF_001467925.1)**	**'Legionella resiliens' (GCF_021344005.1)**	12.8	[10.1 - 16.0]	23.2	[20.9 - 25.7]	13.2	[10.8 - 15.9]	2.68
**'Legionella sp. PATHC038' (GCF_026191355.1)**	**'Legionella sainthelensi' (GCF_900637685.1)**	15.8	[12.9 - 19.3]	23.2	[20.9 - 25.7]	15.9	[13.4 - 18.8]	1.77
**'Legionella qingyii' (GCF_003184185.1)**	**'Legionella cincinnatiensis' (GCF_900452415.1)**	16.0	[13.1 - 19.5]	23.2	[20.9 - 25.6]	16.1	[13.6 - 19.1]	0.94
**'Fluoribacter gormanii' (GCF_900156395.1)**	**'Legionella wadsworthii' (GCF_900452925.1)**	19.7	[16.5 - 23.3]	23.2	[20.9 - 25.6]	19.3	[16.6 - 22.3]	0.0
**'Legionella waltersii' (GCF_900187095.1)**	**'Legionella parisiensis' (GCF_900461585.1)**	13.9	[11.1 - 17.2]	23.2	[20.9 - 25.6]	14.2	[11.7 - 17.0]	1.2
**'Legionella santicrucis' (GCF_001468135.1)**	**'Legionella wadsworthii' (GCF_900452925.1)**	15.6	[12.7 - 19.1]	23.2	[20.9 - 25.7]	15.7	[13.2 - 18.7]	1.43

Query	Subject	d_0	C.I. d_0	d_4	C.I. d_4	d_6	C.I. d_6	Diff. G+C Percent
**'Legionella qingyii' (GCF_003184185.1)**	**'Legionella bononiensis' (GCF_016786415.1)**	13.5	[10.8 - 16.9]	23.1	[20.9 - 25.6]	13.9	[11.5 - 16.7]	1.24
**'Legionella sp. PATHC038' (GCF_026191355.1)**	**'Legionella wadsworthii' (GCF_900452925.1)**	17.5	[14.5 - 21.1]	23.1	[20.8 - 25.5]	17.4	[14.8 - 20.4]	0.74
**'Legionella saoudiensis' (GCF_001465875.1)**	**'Legionella steelei' (GCF_001468005.1)**	14.4	[11.6 - 17.8]	23.1	[20.8 - 25.5]	14.7	[12.2 - 17.5]	0.66
**'Legionella drancourtii LLAP12' (GCF_000162755.2)**	**'Legionella steigerwaltii' (GCF_900452835.1)**	14.3	[11.4 - 17.6]	23.1	[20.8 - 25.6]	14.5	[12.1 - 17.4]	0.93
**'Legionella longbeachae' (GCF_019930685.1)**	**'Legionella steigerwaltii' (GCF_900452835.1)**	15.9	[13.0 - 19.4]	23.1	[20.9 - 25.6]	16.0	[13.5 - 18.9]	1.25
**'Legionella anisa' (GCA_019930885.1)**	**'Legionella sainthelensi' (GCF_900637685.1)**	16.6	[13.6 - 20.1]	23.1	[20.8 - 25.6]	16.6	[14.1 - 19.6]	1.22
**'Legionella cincinnatiensis' (GCF_900452415.1)**	**'Legionella nagasakiensis' (GCF_900639915.1)**	13.0	[10.3 - 16.3]	23.1	[20.8 - 25.6]	13.3	[11.0 - 16.1]	4.23
**'Legionella steelei' (GCF_001468005.1)**	**'Legionella gratiana' (GCF_900452545.1)**	17.0	[14.0 - 20.5]	23.1	[20.8 - 25.6]	17.0	[14.4 - 20.0]	1.79
**'Legionella norrlandica' (GCF_000770585.1)**	**'Legionella sp. PATHC032' (GCF_026191185.1)**	20.6	[17.4 - 24.2]	23.1	[20.8 - 25.6]	20.1	[17.3 - 23.1]	0.56
**'Legionella steelei' (GCF_001468005.1)**	**'Legionella santicrucis' (GCF_001468135.1)**	16.3	[13.3 - 19.8]	23.1	[20.8 - 25.6]	16.4	[13.8 - 19.3]	2.11
**'Legionella anisa' (GCA_019930885.1)**	**'Legionella rubrilucens' (GCF_900640015.1)**	13.4	[10.7 - 16.8]	23.1	[20.8 - 25.6]	13.8	[11.4 - 16.6]	9.25
**'Legionella nagasakiensis' (GCF_900639915.1)**	**'Legionella bozemanae' (GCF_900640135.1)**	12.9	[10.2 - 16.2]	23.1	[20.8 - 25.6]	13.3	[10.9 - 16.0]	3.18
**'Legionella drancourtii LLAP12' (GCF_000162755.2)**	**'Legionella sp. PATHC035' (GCF_026191115.1)**	14.4	[11.6 - 17.8]	23.0	[20.7 - 25.4]	14.7	[12.2 - 17.5]	0.19
**'Legionella moravica' (GCF_900452715.1)**	**'Legionella sainthelensi' (GCF_900637685.1)**	13.6	[10.8 - 16.9]	23.0	[20.7 - 25.4]	13.9	[11.5 - 16.7]	3.07
**'Legionella longbeachae' (GCF_019930685.1)**	**'Legionella taurinensis' (GCF_900452865.1)**	12.9	[10.2 - 16.2]	23.0	[20.7 - 25.5]	13.3	[10.9 - 16.0]	10.98
**'Legionella septentrionalis' (GCF_003989745.1)**	**'Legionella waltersii' (GCF_900187095.1)**	13.2	[10.5 - 16.5]	23.0	[20.7 - 25.5]	13.6	[11.2 - 16.4]	2.98
**'Legionella sainthelensi' (GCF_900637685.1)**	**'Legionella bozemanae' (GCF_900640135.1)**	16.2	[13.2 - 19.7]	23.0	[20.7 - 25.4]	16.2	[13.7 - 19.2]	0.86
**'Legionella steelei' (GCF_001468005.1)**	**'Legionella sainthelensi' (GCF_900637685.1)**	16.1	[13.1 - 19.6]	23.0	[20.7 - 25.4]	16.2	[13.6 - 19.1]	1.71
**'Legionella drancourtii LLAP12' (GCF_000162755.2)**	**'Legionella steelei' (GCF_001468005.1)**	14.8	[12.0 - 18.3]	23.0	[20.7 - 25.4]	15.0	[12.6 - 17.9]	0.48
**'Legionella longbeachae' (GCF_019930685.1)**	**'Legionella sp. PATHC032' (GCF_026191185.1)**	13.3	[10.6 - 16.6]	23.0	[20.7 - 25.4]	13.6	[11.3 - 16.4]	0.96
**'Legionella drancourtii LLAP12' (GCF_000162755.2)**	**'Legionella bozemanae' (GCF_900640135.1)**	14.3	[11.5 - 17.7]	23.0	[20.7 - 25.4]	14.6	[12.1 - 17.4]	1.33
**'Legionella longbeachae' (GCF_019930685.1)**	**'Legionella bozemanae' (GCF_900640135.1)**	16.3	[13.4 - 19.8]	23.0	[20.8 - 25.5]	16.4	[13.8 - 19.3]	0.85
**'Legionella steelei' (GCF_001468005.1)**	**'Legionella sp. PATHC032' (GCF_026191185.1)**	13.4	[10.7 - 16.8]	23.0	[20.7 - 25.4]	13.8	[11.4 - 16.6]	0.74
**'Legionella longbeachae' (GCF_019930685.1)**	**'Fluoribacter gormanii' (GCF_900156395.1)**	16.0	[13.0 - 19.5]	23.0	[20.7 - 25.4]	16.1	[13.5 - 19.0]	1.01

Query	Subject	d_0	C.I. d_0	d_4	C.I. d_4	d_6	C.I. d_6	Diff. G+C Percent
**'Legionella waltersii' (GCF_900187095.1)**	**'Legionella wadsworthii' (GCF_900452925.1)**	13.1	[10.4 - 16.4]	23.0	[20.7 - 25.5]	13.5	[11.1 - 16.2]	1.09
**'Legionella drancourtii LLAP12' (GCF_000162755.2)**	**'Legionella worsleiensis' (GCF_900453045.1)**	13.8	[11.0 - 17.1]	22.9	[20.6 - 25.4]	14.1	[11.7 - 16.9]	1.17
**'Legionella fallonii LLAP-10' (GCF_000953135.1)**	**'Legionella antarctica' (GCF_011764505.1)**	15.6	[12.6 - 19.0]	22.9	[20.6 - 25.3]	15.7	[13.2 - 18.6]	0.82
**'Legionella fallonii LLAP-10' (GCF_000953135.1)**	**'Legionella sp. PATHC039' (GCF_026191275.1)**	14.2	[11.4 - 17.5]	22.9	[20.6 - 25.3]	14.4	[12.0 - 17.3]	0.19
**'Legionella longbeachae' (GCF_019930685.1)**	**'Legionella maioricensis' (GCF_023618015.1)**	13.5	[10.7 - 16.8]	22.9	[20.6 - 25.4]	13.8	[11.4 - 16.6]	2.22
**'Legionella longbeachae' (GCF_019930685.1)**	**'Legionella sp. PATHC038' (GCF_026191355.1)**	15.5	[12.6 - 18.9]	22.9	[20.6 - 25.3]	15.6	[13.1 - 18.5]	1.76
**'Legionella sp. PATHC035' (GCF_026191115.1)**	**'Legionella sainthelensi' (GCF_900637685.1)**	15.6	[12.7 - 19.0]	22.9	[20.6 - 25.4]	15.7	[13.2 - 18.6]	2.38
**'Legionella resiliens' (GCF_021344005.1)**	**'Legionella waltersii' (GCF_900187095.1)**	13.3	[10.6 - 16.6]	22.9	[20.6 - 25.3]	13.6	[11.3 - 16.4]	1.02
**'Legionella cherrii' (GCF_900635815.1)**	**'Legionella sainthelensi' (GCF_900637685.1)**	15.8	[12.8 - 19.2]	22.9	[20.6 - 25.3]	15.9	[13.4 - 18.8]	1.84
**'Fluoribacter dumoffii NY 23' (GCF_000236165.1)**	**'Legionella norrlandica' (GCF_000770585.1)**	13.4	[10.7 - 16.7]	22.9	[20.6 - 25.3]	13.7	[11.4 - 16.5]	2.17
**'Legionella wadsworthii' (GCF_900452925.1)**	**'Legionella bozemanae' (GCF_900640135.1)**	19.9	[16.8 - 23.5]	22.9	[20.6 - 25.3]	19.5	[16.7 - 22.5]	0.17
**'Legionella qingyii' (GCF_003184185.1)**	**'Legionella sainthelensi' (GCF_900637685.1)**	15.8	[12.8 - 19.2]	22.9	[20.6 - 25.3]	15.9	[13.4 - 18.8]	0.75
**'Legionella moravica' (GCF_900452715.1)**	**'Legionella worsleiensis' (GCF_900453045.1)**	22.3	[19.0 - 25.9]	22.9	[20.6 - 25.4]	21.4	[18.6 - 24.5]	0.29
**'Legionella sp. PATHC039' (GCF_026191275.1)**	**'Legionella cherrii' (GCF_900635815.1)**	13.4	[10.7 - 16.7]	22.9	[20.6 - 25.3]	13.7	[11.4 - 16.5]	0.76
**'Legionella sp. PATHC035' (GCF_026191115.1)**	**'Legionella taurinensis' (GCF_900452865.1)**	12.8	[10.1 - 16.1]	22.9	[20.6 - 25.4]	13.2	[10.9 - 16.0]	8.62
**'Legionella sp. PATHC038' (GCF_026191355.1)**	**'Legionella waltersii' (GCF_900187095.1)**	13.4	[10.7 - 16.7]	22.9	[20.6 - 25.4]	13.8	[11.4 - 16.5]	0.35
**'Fluoribacter dumoffii NY 23' (GCF_000236165.1)**	**'Legionella tucsonensis' (GCF_001468035.1)**	22.1	[18.8 - 25.7]	22.9	[20.6 - 25.3]	21.2	[18.4 - 24.3]	2.23
**'Legionella longbeachae' (GCF_019930685.1)**	**'Legionella quateirensis' (GCF_900452695.1)**	13.4	[10.7 - 16.7]	22.9	[20.6 - 25.4]	13.8	[11.4 - 16.6]	1.99
**'Legionella fallonii LLAP-10' (GCF_000953135.1)**	**'Legionella sainthelensi' (GCF_900637685.1)**	13.5	[10.7 - 16.8]	22.9	[20.7 - 25.4]	13.8	[11.4 - 16.6]	1.27
**'Legionella quateirensis' (GCF_900452695.1)**	**'Legionella worsleiensis' (GCF_900453045.1)**	20.6	[17.4 - 24.2]	22.9	[20.6 - 25.3]	20.0	[17.3 - 23.1]	1.35
**'Legionella tucsonensis' (GCF_001468035.1)**	**'Legionella longbeachae' (GCF_019930685.1)**	16.1	[13.1 - 19.6]	22.9	[20.6 - 25.3]	16.2	[13.6 - 19.1]	0.34
**'Legionella resiliens' (GCF_021344005.1)**	**'Legionella quateirensis' (GCF_900452695.1)**	13.6	[10.8 - 16.9]	22.9	[20.6 - 25.3]	13.9	[11.5 - 16.7]	0.9
**'Legionella septentrionalis' (GCF_003989745.1)**	**'Legionella sp. PATHC039' (GCF_026191275.1)**	13.2	[10.4 - 16.5]	22.8	[20.5 - 25.3]	13.5	[11.2 - 16.3]	4.02
**'Legionella qingyii' (GCF_003184185.1)**	**'Legionella maioricensis' (GCF_023618015.1)**	13.6	[10.8 - 16.9]	22.8	[20.5 - 25.3]	13.9	[11.5 - 16.7]	1.48

Query	Subject	d_0	C.I. d_0	d_4	C.I. d_4	d_6	C.I. d_6	Diff. G+C Percent
**'Legionella fallonii LLAP-10' (GCF_000953135.1)**	**'Legionella sp. PATHC035' (GCF_026191115.1)**	13.7	[10.9 - 17.0]	22.8	[20.5 - 25.3]	14.0	[11.6 - 16.8]	1.11
**'Legionella sp. PATHC035' (GCF_026191115.1)**	**'Legionella sp. PATHC032' (GCF_026191185.1)**	13.4	[10.6 - 16.7]	22.8	[20.5 - 25.3]	13.7	[11.3 - 16.5]	1.41
**'Legionella drancourtii LLAP12' (GCF_000162755.2)**	**'Legionella gratiana' (GCF_900452545.1)**	14.3	[11.5 - 17.7]	22.8	[20.5 - 25.3]	14.6	[12.1 - 17.4]	2.27
**'Legionella pneumophila' (GCF_001941585.1)**	**'Legionella cincinnatiensis' (GCF_900452415.1)**	13.6	[10.8 - 16.9]	22.8	[20.6 - 25.3]	13.9	[11.5 - 16.7]	1.46
**'Legionella taurinensis' (GCF_900452865.1)**	**'Legionella cherrii' (GCF_900635815.1)**	12.8	[10.2 - 16.1]	22.8	[20.5 - 25.2]	13.2	[10.9 - 16.0]	9.16
**'Legionella wadsworthii' (GCF_900452925.1)**	**'Legionella cherrii' (GCF_900635815.1)**	17.7	[14.6 - 21.2]	22.8	[20.5 - 25.2]	17.5	[14.9 - 20.5]	0.82
**'Fluoribacter dumoffii NY 23' (GCF_000236165.1)**	**'Legionella cincinnatiensis' (GCF_900452415.1)**	15.7	[12.7 - 19.1]	22.8	[20.5 - 25.2]	15.8	[13.3 - 18.7]	2.77
**'Legionella oakridgensis' (GCF_001467925.1)**	**'Legionella gratiana' (GCF_900452545.1)**	13.1	[10.4 - 16.4]	22.8	[20.5 - 25.2]	13.4	[11.1 - 16.2]	3.86
**'Legionella fallonii LLAP-10' (GCF_000953135.1)**	**'Legionella santicrucis' (GCF_001468135.1)**	13.6	[10.8 - 16.9]	22.8	[20.5 - 25.3]	13.9	[11.5 - 16.7]	1.67
**'Legionella saoudiensis' (GCF_001465875.1)**	**'Legionella wadsworthii' (GCF_900452925.1)**	13.9	[11.1 - 17.2]	22.8	[20.6 - 25.3]	14.2	[11.8 - 17.0]	1.34
**'Legionella santicrucis' (GCF_001468135.1)**	**'Legionella pneumophila' (GCF_001941585.1)**	13.6	[10.8 - 16.9]	22.8	[20.5 - 25.3]	13.9	[11.5 - 16.7]	1.67
**'Legionella taurinensis' (GCF_900452865.1)**	**'Legionella wadsworthii' (GCF_900452925.1)**	12.9	[10.2 - 16.2]	22.8	[20.5 - 25.3]	13.3	[10.9 - 16.0]	9.97
**'Fluoribacter dumoffii NY 23' (GCF_000236165.1)**	**'Legionella qingyii' (GCF_003184185.1)**	21.4	[18.1 - 25.0]	22.8	[20.5 - 25.2]	20.6	[17.9 - 23.7]	1.84
**'Legionella antarctica' (GCF_011764505.1)**	**'Legionella wadsworthii' (GCF_900452925.1)**	13.3	[10.6 - 16.6]	22.7	[20.4 - 25.2]	13.7	[11.3 - 16.5]	1.06
**'Legionella fallonii LLAP-10' (GCF_000953135.1)**	**'Legionella sp. PATHC032' (GCF_026191185.1)**	14.1	[11.3 - 17.5]	22.7	[20.4 - 25.1]	14.4	[12.0 - 17.2]	0.3
**'Legionella qingyii' (GCF_003184185.1)**	**'Legionella longbeachae' (GCF_019930685.1)**	15.9	[13.0 - 19.4]	22.7	[20.5 - 25.2]	16.0	[13.5 - 18.9]	0.73
**'Legionella fallonii LLAP-10' (GCF_000953135.1)**	**'Legionella cherrii' (GCF_900635815.1)**	13.5	[10.7 - 16.8]	22.7	[20.4 - 25.2]	13.8	[11.4 - 16.6]	0.58
**'Legionella tucsonensis' (GCF_001468035.1)**	**'Legionella sainthelensi' (GCF_900637685.1)**	16.7	[13.7 - 20.2]	22.7	[20.5 - 25.2]	16.7	[14.1 - 19.6]	0.35
**'Legionella drancourtii LLAP12' (GCF_000162755.2)**	**'Fluoribacter gormanii' (GCF_900156395.1)**	14.4	[11.5 - 17.8]	22.7	[20.4 - 25.1]	14.6	[12.2 - 17.5]	1.16
**'Legionella fallonii LLAP-10' (GCF_000953135.1)**	**'Legionella cincinnatiensis' (GCF_900452415.1)**	13.5	[10.8 - 16.8]	22.7	[20.4 - 25.1]	13.8	[11.4 - 16.6]	1.46
**'Legionella longbeachae' (GCF_019930685.1)**	**'Legionella cherrii' (GCF_900635815.1)**	15.7	[12.8 - 19.2]	22.7	[20.4 - 25.2]	15.8	[13.3 - 18.7]	1.83
**'Legionella drancourtii LLAP12' (GCF_000162755.2)**	**'Legionella sp. PATHC038' (GCF_026191355.1)**	14.3	[11.5 - 17.7]	22.7	[20.5 - 25.2]	14.5	[12.1 - 17.4]	0.42
**'Legionella sp. PATHC038' (GCF_026191355.1)**	**'Legionella taurinensis' (GCF_900452865.1)**	12.8	[10.1 - 16.1]	22.7	[20.4 - 25.2]	13.2	[10.9 - 16.0]	9.23
**'Legionella tucsonensis' (GCF_001468035.1)**	**'Legionella taurinensis' (GCF_900452865.1)**	12.8	[10.1 - 16.0]	22.7	[20.4 - 25.1]	13.2	[10.8 - 15.9]	10.64

Query	Subject	d_0	C.I. d_0	d_4	C.I. d_4	d_6	C.I. d_6	Diff. G+C Percent
**'Legionella bononiensis' (GCF_016786415.1)**	**'Legionella worsleiensis' (GCF_900453045.1)**	20.1	[16.9 - 23.7]	22.7	[20.5 - 25.2]	19.6	[16.9 - 22.6]	1.37
**'Legionella drancourtii LLAP12' (GCF_000162755.2)**	**'Legionella sp. PATHC039' (GCF_026191275.1)**	13.4	[10.7 - 16.8]	22.7	[20.5 - 25.2]	13.8	[11.4 - 16.6]	1.11
**'Legionella quateirensis' (GCF_900452695.1)**	**'Legionella bozemanae' (GCF_900640135.1)**	13.6	[10.9 - 17.0]	22.7	[20.4 - 25.2]	14.0	[11.5 - 16.8]	1.15
**'Legionella sp. PATHC038' (GCF_026191355.1)**	**'Legionella nagasakiensis' (GCF_900639915.1)**	12.9	[10.2 - 16.2]	22.7	[20.4 - 25.2]	13.2	[10.9 - 16.0]	2.27
**'Legionella anisa' (GCA_019930885.1)**	**'Legionella waltersii' (GCF_900187095.1)**	13.8	[11.0 - 17.2]	22.6	[20.3 - 25.0]	14.1	[11.7 - 16.9]	0.89
**'Legionella antarctica' (GCF_011764505.1)**	**'Legionella longbeachae' (GCF_019930685.1)**	13.3	[10.6 - 16.6]	22.6	[20.3 - 25.0]	13.7	[11.3 - 16.5]	2.07
**'Legionella bononiensis' (GCF_016786415.1)**	**'Legionella maioricensis' (GCF_023618015.1)**	16.1	[13.2 - 19.6]	22.6	[20.4 - 25.1]	16.2	[13.6 - 19.1]	0.24
**'Legionella drancourtii LLAP12' (GCF_000162755.2)**	**'Legionella moravica' (GCF_900452715.1)**	14.0	[11.2 - 17.4]	22.6	[20.4 - 25.1]	14.3	[11.9 - 17.1]	0.88
**'Legionella anisa' (GCA_019930885.1)**	**'Legionella oakridgensis' (GCF_001467925.1)**	12.8	[10.2 - 16.1]	22.6	[20.4 - 25.1]	13.2	[10.9 - 16.0]	2.55
**'Fluoribacter dumoffii NY 23' (GCF_000236165.1)**	**'Legionella taurinensis' (GCF_900452865.1)**	13.2	[10.5 - 16.5]	22.6	[20.3 - 25.1]	13.5	[11.2 - 16.3]	8.42
**'Legionella pneumophila' (GCF_001941585.1)**	**'Legionella sp. PATHC035' (GCF_026191115.1)**	13.4	[10.6 - 16.7]	22.6	[20.3 - 25.0]	13.7	[11.3 - 16.5]	1.11
**'Legionella oakridgensis' (GCF_001467925.1)**	**'Legionella steigerwaltii' (GCF_900452835.1)**	12.8	[10.2 - 16.1]	22.6	[20.3 - 25.1]	13.2	[10.9 - 16.0]	2.51
**'Legionella fallonii LLAP-10' (GCF_000953135.1)**	**'Legionella qingyii' (GCF_003184185.1)**	13.5	[10.8 - 16.9]	22.6	[20.3 - 25.1]	13.9	[11.5 - 16.7]	0.52
**'Legionella wadsworthii' (GCF_900452925.1)**	**'Legionella worsleiensis' (GCF_900453045.1)**	13.3	[10.6 - 16.7]	22.6	[20.3 - 25.0]	13.7	[11.3 - 16.5]	2.33
**'Legionella norrlandica' (GCF_000770585.1)**	**'Legionella steigerwaltii' (GCF_900452835.1)**	13.4	[10.6 - 16.7]	22.6	[20.3 - 25.0]	13.7	[11.3 - 16.5]	0.85
**'Fluoribacter gormanii' (GCF_900156395.1)**	**'Legionella sainthelensi' (GCF_900637685.1)**	16.4	[13.4 - 19.9]	22.6	[20.3 - 25.1]	16.4	[13.8 - 19.3]	1.02
**'Legionella fallonii LLAP-10' (GCF_000953135.1)**	**'Legionella parisiensis' (GCF_900461585.1)**	13.6	[10.9 - 17.0]	22.6	[20.3 - 25.0]	13.9	[11.5 - 16.8]	0.35
**'Legionella resiliens' (GCF_021344005.1)**	**'Legionella wadsworthii' (GCF_900452925.1)**	20.0	[16.8 - 23.6]	22.5	[20.3 - 25.0]	19.5	[16.7 - 22.5]	0.07
**'Legionella antarctica' (GCF_011764505.1)**	**'Legionella parisiensis' (GCF_900461585.1)**	13.7	[10.9 - 17.0]	22.5	[20.2 - 24.9]	14.0	[11.6 - 16.8]	1.17
**'Legionella steelei' (GCF_001468005.1)**	**'Legionella longbeachae' (GCF_019930685.1)**	15.9	[12.9 - 19.3]	22.5	[20.2 - 24.9]	16.0	[13.4 - 18.9]	1.7
**'Legionella tucsonensis' (GCF_001468035.1)**	**'Legionella wadsworthii' (GCF_900452925.1)**	20.3	[17.1 - 23.9]	22.5	[20.2 - 25.0]	19.7	[17.0 - 22.8]	0.67
**'Legionella sp. PATHC032' (GCF_026191185.1)**	**'Legionella bozemanae' (GCF_900640135.1)**	13.8	[11.0 - 17.1]	22.5	[20.3 - 25.0]	14.1	[11.7 - 16.9]	0.11
**'Legionella saoudiensis' (GCF_001465875.1)**	**'Legionella cherrii' (GCF_900635815.1)**	14.2	[11.3 - 17.5]	22.5	[20.2 - 24.9]	14.4	[12.0 - 17.3]	0.53
**'Legionella antarctica' (GCF_011764505.1)**	**'Legionella sp. PATHC038' (GCF_026191355.1)**	13.6	[10.8 - 16.9]	22.5	[20.2 - 24.9]	13.9	[11.5 - 16.7]	0.32
**'Legionella fallonii LLAP-10' (GCF_000953135.1)**	**'Legionella pneumophila' (GCF_001941585.1)**	14.2	[11.3 - 17.5]	22.5	[20.2 - 24.9]	14.4	[12.0 - 17.3]	0.0

Query	Subject	d_0	C.I. d_0	d_4	C.I. d_4	d_6	C.I. d_6	Diff. G+C Percent
**'Legionella pneumophila' (GCF_001941585.1)**	**'Fluoribacter gormanii' (GCF_900156395.1)**	13.4	[10.7 - 16.7]	22.5	[20.2 - 24.9]	13.7	[11.4 - 16.5]	0.25
**'Legionella saoudiensis' (GCF_001465875.1)**	**'Legionella sp. PATHC038' (GCF_026191355.1)**	14.1	[11.3 - 17.5]	22.5	[20.2 - 25.0]	14.4	[12.0 - 17.3]	0.6
**'Legionella santicrucis' (GCF_001468135.1)**	**'Legionella taurinensis' (GCF_900452865.1)**	12.8	[10.1 - 16.1]	22.5	[20.2 - 24.9]	13.2	[10.9 - 16.0]	11.4
**'Legionella santicrucis' (GCF_001468135.1)**	**'Legionella antarctica' (GCF_011764505.1)**	13.6	[10.8 - 16.9]	22.5	[20.3 - 25.0]	13.9	[11.5 - 16.7]	2.49
**'Legionella pneumophila' (GCF_001941585.1)**	**'Legionella nagasakiensis' (GCF_900639915.1)**	13.2	[10.4 - 16.5]	22.4	[20.2 - 24.9]	13.5	[11.1 - 16.3]	2.77
**'Legionella longbeachae' (GCF_019930685.1)**	**'Legionella sp. PATHC035' (GCF_026191115.1)**	15.7	[12.7 - 19.1]	22.4	[20.1 - 24.8]	15.8	[13.2 - 18.7]	2.36
**'Legionella drancourtii LLAP12' (GCF_000162755.2)**	**'Legionella maioricensis' (GCF_023618015.1)**	13.9	[11.1 - 17.3]	22.4	[20.1 - 24.8]	14.2	[11.8 - 17.0]	0.04
**'Legionella antarctica' (GCF_011764505.1)**	**'Legionella cincinnatiensis' (GCF_900452415.1)**	13.6	[10.8 - 16.9]	22.4	[20.2 - 24.9]	13.9	[11.5 - 16.7]	2.27
**'Legionella shakespearei DSM 23087' (GCF_000373765.1)**	**'Legionella steelei' (GCF_001468005.1)**	13.4	[10.6 - 16.7]	22.4	[20.1 - 24.8]	13.7	[11.3 - 16.5]	2.93
**'Legionella cincinnatiensis' (GCF_900452415.1)**	**'Legionella wadsworthii' (GCF_900452925.1)**	15.3	[12.4 - 18.7]	22.4	[20.1 - 24.8]	15.4	[12.9 - 18.3]	1.22
**'Legionella bononiensis' (GCF_016786415.1)**	**'Legionella rubrilucens' (GCF_900640015.1)**	13.2	[10.5 - 16.5]	22.4	[20.1 - 24.8]	13.6	[11.2 - 16.4]	8.49
**'Legionella worsleiensis' (GCF_900453045.1)**	**'Legionella parisiensis' (GCF_900461585.1)**	13.5	[10.8 - 16.9]	22.4	[20.2 - 24.9]	13.8	[11.5 - 16.6]	2.44
**'Legionella sp. PATHC035' (GCF_026191115.1)**	**'Legionella waltersii' (GCF_900187095.1)**	13.4	[10.6 - 16.7]	22.4	[20.1 - 24.8]	13.7	[11.3 - 16.5]	0.26
**'Legionella longbeachae' (GCF_019930685.1)**	**'Legionella nagasakiensis' (GCF_900639915.1)**	13.0	[10.3 - 16.3]	22.4	[20.1 - 24.8]	13.4	[11.0 - 16.1]	4.02
**'Legionella drancourtii LLAP12' (GCF_000162755.2)**	**'Fluoribacter dumoffii NY 23' (GCF_000236165.1)**	14.4	[11.6 - 17.8]	22.4	[20.1 - 24.8]	14.7	[12.2 - 17.5]	0.4
**'Legionella drancourtii LLAP12' (GCF_000162755.2)**	**'Legionella antarctica' (GCF_011764505.1)**	13.8	[11.0 - 17.1]	22.4	[20.2 - 24.9]	14.1	[11.7 - 16.9]	0.1
**'Legionella drancourtii LLAP12' (GCF_000162755.2)**	**'Legionella saoudiensis' (GCF_001465875.1)**	15.9	[13.0 - 19.4]	22.4	[20.1 - 24.8]	16.0	[13.5 - 18.9]	0.18
**'Legionella fallonii LLAP-10' (GCF_000953135.1)**	**'Legionella longbeachae' (GCF_019930685.1)**	13.4	[10.7 - 16.7]	22.4	[20.2 - 24.9]	13.7	[11.4 - 16.5]	1.25
**'Legionella norrlandica' (GCF_000770585.1)**	**'Legionella santicrucis' (GCF_001468135.1)**	13.7	[10.9 - 17.0]	22.3	[20.0 - 24.7]	14.0	[11.6 - 16.8]	0.82
**'Legionella anisa' (GCA_019930885.1)**	**'Legionella moravica' (GCF_900452715.1)**	13.7	[10.9 - 17.0]	22.3	[20.1 - 24.8]	14.0	[11.6 - 16.8]	1.84
**'Legionella steelei' (GCF_001468005.1)**	**'Legionella sp. PATHC039' (GCF_026191275.1)**	13.7	[11.0 - 17.1]	22.3	[20.1 - 24.8]	14.0	[11.6 - 16.9]	0.63
**'Legionella waltersii' (GCF_900187095.1)**	**'Legionella rubrilucens' (GCF_900640015.1)**	13.2	[10.5 - 16.5]	22.3	[20.0 - 24.8]	13.6	[11.2 - 16.3]	8.36
**'Legionella resiliens' (GCF_021344005.1)**	**'Legionella moravica' (GCF_900452715.1)**	13.5	[10.8 - 16.9]	22.3	[20.0 - 24.7]	13.8	[11.5 - 16.7]	1.97

Query	Subject	d_0	C.I. d_0	d_4	C.I. d_4	d_6	C.I. d_6	Diff. G+C Percent
**'Legionella moravica' (GCF_900452715.1)**	**'Legionella steigerwaltii' (GCF_900452835.1)**	13.7	[10.9 - 17.0]	22.3	[20.1 - 24.8]	14.0	[11.6 - 16.8]	1.81
**'Legionella antarctica' (GCF_011764505.1)**	**'Legionella sainthelensi' (GCF_900637685.1)**	13.5	[10.7 - 16.8]	22.3	[20.1 - 24.8]	13.8	[11.4 - 16.6]	2.08
**'Legionella drancourtii LLAP12' (GCF_000162755.2)**	**'Legionella cherrii' (GCF_900635815.1)**	14.4	[11.6 - 17.8]	22.3	[20.0 - 24.8]	14.7	[12.2 - 17.5]	0.34
**'Legionella steelei' (GCF_001468005.1)**	**'Legionella quateirensis' (GCF_900452695.1)**	13.8	[11.1 - 17.2]	22.3	[20.0 - 24.8]	14.1	[11.7 - 17.0]	0.29
**'Legionella antarctica' (GCF_011764505.1)**	**'Legionella resiliens' (GCF_021344005.1)**	13.3	[10.6 - 16.7]	22.2	[20.0 - 24.7]	13.7	[11.3 - 16.5]	0.99
**'Legionella pneumophila' (GCF_001941585.1)**	**'Legionella worsleiensis' (GCF_900453045.1)**	14.4	[11.5 - 17.8]	22.2	[19.9 - 24.6]	14.6	[12.2 - 17.5]	2.09
**'Fluoribacter dumoffii NY 23' (GCF_000236165.1)**	**'Legionella antarctica' (GCF_011764505.1)**	13.5	[10.7 - 16.8]	22.2	[19.9 - 24.6]	13.8	[11.4 - 16.6]	0.5
**'Legionella sp. PATHC039' (GCF_026191275.1)**	**'Legionella waltersii' (GCF_900187095.1)**	14.7	[11.8 - 18.1]	22.2	[19.9 - 24.6]	14.9	[12.4 - 17.7]	1.04
**'Legionella quateirensis' (GCF_900452695.1)**	**'Legionella cherrii' (GCF_900635815.1)**	13.6	[10.9 - 17.0]	22.2	[19.9 - 24.7]	14.0	[11.6 - 16.8]	0.16
**'Legionella qingyii' (GCF_003184185.1)**	**'Legionella sp. PATHC039' (GCF_026191275.1)**	13.3	[10.6 - 16.7]	22.2	[20.0 - 24.7]	13.7	[11.3 - 16.5]	0.33
**'Legionella sp. PATHC032' (GCF_026191185.1)**	**'Legionella parisiensis' (GCF_900461585.1)**	13.7	[10.9 - 17.0]	22.2	[19.9 - 24.6]	14.0	[11.6 - 16.8]	0.06
**'Fluoribacter dumoffii NY 23' (GCF_000236165.1)**	**'Legionella waltersii' (GCF_900187095.1)**	13.4	[10.7 - 16.7]	22.1	[19.8 - 24.5]	13.8	[11.4 - 16.5]	0.47
**'Legionella tucsonensis' (GCF_001468035.1)**	**'Legionella antarctica' (GCF_011764505.1)**	13.6	[10.9 - 17.0]	22.1	[19.9 - 24.6]	14.0	[11.6 - 16.8]	1.73
**'Legionella steelei' (GCF_001468005.1)**	**'Legionella wadsworthii' (GCF_900452925.1)**	17.9	[14.9 - 21.5]	22.1	[19.8 - 24.5]	17.7	[15.1 - 20.7]	0.68
**'Legionella fallonii LLAP-10' (GCF_000953135.1)**	**'Legionella wadsworthii' (GCF_900452925.1)**	13.4	[10.7 - 16.7]	22.1	[19.8 - 24.6]	13.8	[11.4 - 16.6]	0.24
**'Legionella steelei' (GCF_001468005.1)**	**'Legionella antarctica' (GCF_011764505.1)**	13.5	[10.7 - 16.8]	22.1	[19.8 - 24.5]	13.8	[11.4 - 16.6]	0.37
**'Legionella steigerwaltii' (GCF_900452835.1)**	**'Legionella worsleiensis' (GCF_900453045.1)**	13.4	[10.7 - 16.8]	22.1	[19.8 - 24.5]	13.8	[11.4 - 16.6]	2.09
**'Legionella longbeachae' (GCF_019930685.1)**	**'Legionella waltersii' (GCF_900187095.1)**	13.2	[10.5 - 16.5]	22.1	[19.9 - 24.6]	13.5	[11.2 - 16.3]	2.1
**'Fluoribacter dumoffii NY 23' (GCF_000236165.1)**	**'Legionella sainthelensi' (GCF_900637685.1)**	15.5	[12.6 - 18.9]	22.1	[19.8 - 24.5]	15.6	[13.1 - 18.5]	2.58
**'Legionella shakespearei DSM 23087' (GCF_000373765.1)**	**'Legionella parisiensis' (GCF_900461585.1)**	13.6	[10.9 - 17.0]	22.1	[19.9 - 24.6]	13.9	[11.5 - 16.7]	3.72
**'Legionella santicrucis' (GCF_001468135.1)**	**'Legionella sp. PATHC032' (GCF_026191185.1)**	13.6	[10.9 - 17.0]	22.1	[19.8 - 24.5]	14.0	[11.6 - 16.8]	1.38
**'Legionella maioricensis' (GCF_023618015.1)**	**'Legionella sainthelensi' (GCF_900637685.1)**	13.6	[10.8 - 16.9]	22.1	[19.8 - 24.6]	13.9	[11.5 - 16.7]	2.23
**'Legionella anisa' (GCA_019930885.1)**	**'Legionella antarctica' (GCF_011764505.1)**	13.5	[10.8 - 16.9]	22.1	[19.8 - 24.5]	13.8	[11.5 - 16.7]	0.86
**'Legionella gratiana' (GCF_900452545.1)**	**'Legionella moravica' (GCF_900452715.1)**	13.8	[11.0 - 17.2]	22.1	[19.9 - 24.6]	14.1	[11.7 - 16.9]	3.15
**'Legionella qingyii' (GCF_003184185.1)**	**'Legionella wadsworthii' (GCF_900452925.1)**	18.3	[15.2 - 21.8]	22.1	[19.9 - 24.6]	18.0	[15.4 - 21.0]	0.28

Query	Subject	d_0	C.I. d_0	d_4	C.I. d_4	d_6	C.I. d_6	Diff. G+C Percent
**'Legionella antarctica' (GCF_011764505.1)**	**'Legionella cherrii' (GCF_900635815.1)**	13.5	[10.8 - 16.9]	22.1	[19.9 - 24.6]	13.9	[11.5 - 16.7]	0.24
**'Legionella pneumophila' (GCF_001941585.1)**	**'Legionella cherrii' (GCF_900635815.1)**	13.4	[10.7 - 16.8]	22.1	[19.8 - 24.5]	13.8	[11.4 - 16.6]	0.57
**'Legionella cincinnatiensis' (GCF_900452415.1)**	**'Legionella moravica' (GCF_900452715.1)**	13.6	[10.8 - 16.9]	22.1	[19.8 - 24.5]	13.9	[11.5 - 16.7]	3.26
**'Legionella antarctica' (GCF_011764505.1)**	**'Legionella gratiana' (GCF_900452545.1)**	13.5	[10.8 - 16.9]	22.1	[19.8 - 24.5]	13.9	[11.5 - 16.7]	2.16
**'Legionella norrlandica' (GCF_000770585.1)**	**'Legionella taurinensis' (GCF_900452865.1)**	12.9	[10.2 - 16.2]	22.1	[19.8 - 24.5]	13.3	[10.9 - 16.0]	10.58
**'Legionella saoudiensis' (GCF_001465875.1)**	**'Legionella longbeachae' (GCF_019930685.1)**	13.7	[10.9 - 17.0]	22.0	[19.7 - 24.5]	14.0	[11.6 - 16.8]	2.36
**'Legionella drancourtii LLAP12' (GCF_000162755.2)**	**'Legionella quateirensis' (GCF_900452695.1)**	13.9	[11.1 - 17.2]	22.0	[19.8 - 24.5]	14.2	[11.7 - 17.0]	0.18
**'Fluoribacter dumoffii NY 23' (GCF_000236165.1)**	**'Legionella fallonii LLAP-10' (GCF_000953135.1)**	13.4	[10.6 - 16.7]	22.0	[19.8 - 24.5]	13.7	[11.3 - 16.5]	1.32
**'Legionella pneumophila' (GCF_001941585.1)**	**'Legionella resiliens' (GCF_021344005.1)**	13.5	[10.8 - 16.8]	22.0	[19.8 - 24.5]	13.8	[11.4 - 16.6]	0.17
**'Legionella norrlandica' (GCF_000770585.1)**	**'Legionella oakridgensis' (GCF_001467925.1)**	13.0	[10.3 - 16.3]	22.0	[19.7 - 24.4]	13.4	[11.0 - 16.2]	3.36
**'Legionella antarctica' (GCF_011764505.1)**	**'Legionella sp. PATHC035' (GCF_026191115.1)**	13.5	[10.7 - 16.8]	22.0	[19.8 - 24.5]	13.8	[11.4 - 16.6]	0.29
**'Legionella septentrionalis' (GCF_003989745.1)**	**'Legionella moravica' (GCF_900452715.1)**	13.1	[10.4 - 16.5]	22.0	[19.7 - 24.4]	13.5	[11.1 - 16.3]	2.03
**'Legionella maioricensis' (GCF_023618015.1)**	**'Legionella cherrii' (GCF_900635815.1)**	13.7	[10.9 - 17.1]	22.0	[19.8 - 24.5]	14.0	[11.6 - 16.8]	0.39
**'Legionella drancourtii LLAP12' (GCF_000162755.2)**	**'Legionella qingyii' (GCF_003184185.1)**	14.3	[11.5 - 17.7]	22.0	[19.7 - 24.4]	14.6	[12.1 - 17.4]	1.44
**'Legionella sp. PATHC032' (GCF_026191185.1)**	**'Fluoribacter gormanii' (GCF_900156395.1)**	13.4	[10.7 - 16.8]	22.0	[19.8 - 24.5]	13.8	[11.4 - 16.6]	0.05
**'Legionella resiliens' (GCF_021344005.1)**	**'Legionella sp. PATHC039' (GCF_026191275.1)**	13.4	[10.7 - 16.7]	22.0	[19.7 - 24.4]	13.7	[11.4 - 16.5]	0.02
**'Legionella norrlandica' (GCF_000770585.1)**	**'Legionella tucsonensis' (GCF_001468035.1)**	13.4	[10.7 - 16.7]	21.9	[19.7 - 24.4]	13.7	[11.4 - 16.5]	0.06
**'Legionella sp. PATHC035' (GCF_026191115.1)**	**'Legionella moravica' (GCF_900452715.1)**	13.5	[10.7 - 16.8]	21.9	[19.7 - 24.4]	13.8	[11.4 - 16.6]	0.69
**'Fluoribacter dumoffii NY 23' (GCF_000236165.1)**	**'Legionella wadsworthii' (GCF_900452925.1)**	19.3	[16.1 - 22.9]	21.9	[19.6 - 24.3]	18.8	[16.1 - 21.8]	1.56
**'Legionella qingyii' (GCF_003184185.1)**	**'Legionella waltersii' (GCF_900187095.1)**	13.2	[10.5 - 16.5]	21.9	[19.7 - 24.4]	13.6	[11.2 - 16.4]	1.37
**'Legionella sp. PATHC039' (GCF_026191275.1)**	**'Legionella worsleiensis' (GCF_900453045.1)**	14.5	[11.7 - 17.9]	21.9	[19.6 - 24.3]	14.7	[12.3 - 17.6]	2.28
**'Legionella saoudiensis' (GCF_001465875.1)**	**'Legionella sp. PATHC032' (GCF_026191185.1)**	13.5	[10.7 - 16.8]	21.9	[19.6 - 24.3]	13.8	[11.4 - 16.6]	1.4
**'Legionella oakridgensis' (GCF_001467925.1)**	**'Legionella pneumophila' (GCF_001941585.1)**	13.2	[10.5 - 16.5]	21.9	[19.6 - 24.3]	13.6	[11.2 - 16.4]	2.51
**'Fluoribacter dumoffii NY 23' (GCF_000236165.1)**	**'Legionella shakespearei DSM 23087' (GCF_000373765.1)**	13.4	[10.6 - 16.7]	21.9	[19.6 - 24.3]	13.7	[11.3 - 16.5]	2.06

Query	Subject	d_0	C.I. d_0	d_4	C.I. d_4	d_6	C.I. d_6	Diff. G+C Percent
**'Legionella drancourtii LLAP12' (GCF_000162755.2)**	**'Legionella cincinnatiensis' (GCF_900452415.1)**	14.1	[11.3 - 17.5]	21.9	[19.7 - 24.4]	14.4	[11.9 - 17.2]	2.38
**'Legionella qingyii' (GCF_003184185.1)**	**'Legionella sp. PATHC032' (GCF_026191185.1)**	13.4	[10.7 - 16.8]	21.9	[19.7 - 24.4]	13.8	[11.4 - 16.6]	0.22
**'Legionella gratiana' (GCF_900452545.1)**	**'Legionella worsleiensis' (GCF_900453045.1)**	13.6	[10.9 - 17.0]	21.9	[19.6 - 24.3]	13.9	[11.5 - 16.7]	3.44
**'Legionella moravica' (GCF_900452715.1)**	**'Legionella taurinensis' (GCF_900452865.1)**	13.0	[10.3 - 16.3]	21.9	[19.6 - 24.3]	13.4	[11.0 - 16.2]	7.93
**'Legionella santicrucis' (GCF_001468135.1)**	**'Legionella waltersii' (GCF_900187095.1)**	13.7	[10.9 - 17.0]	21.9	[19.6 - 24.3]	14.0	[11.6 - 16.8]	2.52
**'Legionella cherrii' (GCF_900635815.1)**	**'Legionella nagasakiensis' (GCF_900639915.1)**	12.8	[10.2 - 16.1]	21.9	[19.6 - 24.3]	13.2	[10.9 - 16.0]	2.2
**'Legionella pneumophila' (GCF_001941585.1)**	**'Legionella wadsworthii' (GCF_900452925.1)**	13.4	[10.6 - 16.7]	21.9	[19.6 - 24.3]	13.7	[11.3 - 16.5]	0.24
**'Legionella drancourtii LLAP12' (GCF_000162755.2)**	**'Legionella longbeachae' (GCF_019930685.1)**	14.0	[11.2 - 17.4]	21.9	[19.7 - 24.4]	14.3	[11.9 - 17.1]	2.17
**'Legionella moravica' (GCF_900452715.1)**	**'Legionella wadsworthii' (GCF_900452925.1)**	13.5	[10.7 - 16.8]	21.8	[19.6 - 24.3]	13.8	[11.4 - 16.6]	2.04
**'Legionella oakridgensis' (GCF_001467925.1)**	**'Legionella cherrii' (GCF_900635815.1)**	12.9	[10.2 - 16.2]	21.8	[19.5 - 24.2]	13.2	[10.9 - 16.0]	1.93
**'Legionella sp. PATHC032' (GCF_026191185.1)**	**'Legionella cherrii' (GCF_900635815.1)**	13.6	[10.8 - 16.9]	21.8	[19.6 - 24.3]	13.9	[11.5 - 16.7]	0.87
**'Legionella oakridgensis' (GCF_001467925.1)**	**'Legionella sainthelensi' (GCF_900637685.1)**	12.9	[10.2 - 16.2]	21.8	[19.5 - 24.2]	13.3	[10.9 - 16.0]	3.78
**'Legionella bononiensis' (GCF_016786415.1)**	**'Legionella taurinensis' (GCF_900452865.1)**	13.2	[10.5 - 16.5]	21.8	[19.5 - 24.2]	13.6	[11.2 - 16.3]	9.01
**'Legionella cincinnatiensis' (GCF_900452415.1)**	**'Legionella taurinensis' (GCF_900452865.1)**	12.9	[10.2 - 16.2]	21.8	[19.6 - 24.3]	13.3	[10.9 - 16.0]	11.19
**'Legionella sp. PATHC035' (GCF_026191115.1)**	**'Legionella worsleiensis' (GCF_900453045.1)**	13.5	[10.8 - 16.9]	21.8	[19.6 - 24.3]	13.9	[11.5 - 16.7]	0.98
**'Legionella saoudiensis' (GCF_001465875.1)**	**'Legionella sainthelensi' (GCF_900637685.1)**	14.0	[11.2 - 17.4]	21.8	[19.6 - 24.3]	14.3	[11.9 - 17.1]	2.37
**'Legionella wadsworthii' (GCF_900452925.1)**	**'Legionella rubrilucens' (GCF_900640015.1)**	12.9	[10.2 - 16.2]	21.8	[19.5 - 24.2]	13.2	[10.9 - 16.0]	9.45
**'Legionella antarctica' (GCF_011764505.1)**	**'Legionella taurinensis' (GCF_900452865.1)**	13.1	[10.3 - 16.4]	21.8	[19.6 - 24.3]	13.4	[11.1 - 16.2]	8.91
**'Legionella shakespearei DSM 23087' (GCF_000373765.1)**	**'Legionella longbeachae' (GCF_019930685.1)**	13.3	[10.5 - 16.6]	21.8	[19.5 - 24.2]	13.6	[11.2 - 16.4]	4.63
**'Legionella norrlandica' (GCF_000770585.1)**	**'Legionella rubrilucens' (GCF_900640015.1)**	13.1	[10.4 - 16.4]	21.8	[19.5 - 24.2]	13.5	[11.1 - 16.3]	10.06
**'Legionella antarctica' (GCF_011764505.1)**	**'Legionella steigerwaltii' (GCF_900452835.1)**	13.5	[10.8 - 16.9]	21.8	[19.5 - 24.2]	13.9	[11.5 - 16.7]	0.82
**'Legionella sp. PATHC039' (GCF_026191275.1)**	**'Legionella quateirensis' (GCF_900452695.1)**	15.2	[12.3 - 18.7]	21.7	[19.5 - 24.2]	15.4	[12.9 - 18.3]	0.92
**'Legionella wadsworthii' (GCF_900452925.1)**	**'Legionella nagasakiensis' (GCF_900639915.1)**	12.9	[10.2 - 16.1]	21.7	[19.5 - 24.2]	13.2	[10.9 - 16.0]	3.01
**'Legionella tucsonensis' (GCF_001468035.1)**	**'Legionella sp. PATHC039' (GCF_026191275.1)**	13.6	[10.9 - 17.0]	21.7	[19.4 - 24.1]	14.0	[11.6 - 16.8]	0.73

Query	Subject	d_0	C.I. d_0	d_4	C.I. d_4	d_6	C.I. d_6	Diff. G+C Percent
**'Legionella pneumophila' (GCF_001941585.1)**	**'Legionella maioricensis' (GCF_023618015.1)**	14.8	[11.9 - 18.2]	21.7	[19.4 - 24.1]	15.0	[12.5 - 17.8]	0.96
**'Legionella drancourtii LLAP12' (GCF_000162755.2)**	**'Legionella resiliens' (GCF_021344005.1)**	14.2	[11.4 - 17.6]	21.7	[19.5 - 24.2]	14.5	[12.0 - 17.3]	1.09
**'Legionella santicrucis' (GCF_001468135.1)**	**'Legionella moravica' (GCF_900452715.1)**	13.5	[10.8 - 16.9]	21.7	[19.4 - 24.1]	13.8	[11.4 - 16.6]	3.47
**'Legionella fallonii LLAP-10' (GCF_000953135.1)**	**'Fluoribacter gormanii' (GCF_900156395.1)**	13.5	[10.7 - 16.8]	21.7	[19.4 - 24.1]	13.8	[11.4 - 16.6]	0.24
**'Legionella drancourtii LLAP12' (GCF_000162755.2)**	**'Legionella pneumophila' (GCF_001941585.1)**	13.3	[10.6 - 16.6]	21.7	[19.5 - 24.2]	13.6	[11.3 - 16.4]	0.92
**'Legionella pneumophila' (GCF_001941585.1)**	**'Legionella bononiensis' (GCF_016786415.1)**	14.9	[12.1 - 18.4]	21.7	[19.4 - 24.1]	15.1	[12.6 - 18.0]	0.72
**'Legionella fallonii LLAP-10' (GCF_000953135.1)**	**'Legionella gratiana' (GCF_900452545.1)**	13.7	[10.9 - 17.0]	21.7	[19.5 - 24.1]	14.0	[11.6 - 16.8]	1.35
**'Legionella qingyii' (GCF_003184185.1)**	**'Legionella moravica' (GCF_900452715.1)**	13.3	[10.6 - 16.7]	21.7	[19.4 - 24.1]	13.7	[11.3 - 16.5]	2.32
**'Legionella drancourtii LLAP12' (GCF_000162755.2)**	**'Legionella tucsonensis' (GCF_001468035.1)**	14.5	[11.7 - 17.9]	21.7	[19.4 - 24.1]	14.7	[12.3 - 17.6]	1.83
**'Legionella fallonii LLAP-10' (GCF_000953135.1)**	**'Legionella tucsonensis' (GCF_001468035.1)**	13.5	[10.8 - 16.9]	21.7	[19.4 - 24.1]	13.9	[11.5 - 16.7]	0.91
**'Legionella maioricensis' (GCF_023618015.1)**	**'Legionella cincinnatiensis' (GCF_900452415.1)**	13.7	[10.9 - 17.0]	21.7	[19.4 - 24.1]	14.0	[11.6 - 16.8]	2.42
**'Legionella tucsonensis' (GCF_001468035.1)**	**'Legionella sp. PATHC032' (GCF_026191185.1)**	13.4	[10.7 - 16.8]	21.6	[19.3 - 24.0]	13.8	[11.4 - 16.6]	0.62
**'Legionella antarctica' (GCF_011764505.1)**	**'Legionella bozemanae' (GCF_900640135.1)**	13.5	[10.7 - 16.8]	21.6	[19.4 - 24.1]	13.8	[11.4 - 16.6]	1.23
**'Legionella pneumophila' (GCF_001941585.1)**	**'Legionella qingyii' (GCF_003184185.1)**	13.4	[10.6 - 16.7]	21.6	[19.4 - 24.1]	13.7	[11.3 - 16.5]	0.52
**'Legionella saoudiensis' (GCF_001465875.1)**	**'Legionella pneumophila' (GCF_001941585.1)**	13.3	[10.5 - 16.6]	21.6	[19.3 - 24.0]	13.6	[11.2 - 16.4]	1.1
**'Legionella taurinensis' (GCF_900452865.1)**	**'Legionella sainthelensi' (GCF_900637685.1)**	12.9	[10.2 - 16.2]	21.6	[19.3 - 24.0]	13.3	[10.9 - 16.1]	11.0
**'Fluoribacter dumoffii NY 23' (GCF_000236165.1)**	**'Legionella worsleiensis' (GCF_900453045.1)**	13.5	[10.8 - 16.9]	21.6	[19.4 - 24.1]	13.8	[11.5 - 16.7]	0.77
**'Legionella moravica' (GCF_900452715.1)**	**'Legionella cherrii' (GCF_900635815.1)**	13.5	[10.7 - 16.8]	21.6	[19.4 - 24.1]	13.8	[11.4 - 16.6]	1.23
**'Legionella fallonii LLAP-10' (GCF_000953135.1)**	**'Legionella resiliens' (GCF_021344005.1)**	13.4	[10.7 - 16.8]	21.6	[19.3 - 24.0]	13.8	[11.4 - 16.6]	0.17
**'Legionella sp. PATHC038' (GCF_026191355.1)**	**'Legionella moravica' (GCF_900452715.1)**	13.5	[10.8 - 16.9]	21.5	[19.3 - 23.9]	13.8	[11.4 - 16.6]	1.3
**'Legionella sp. PATHC032' (GCF_026191185.1)**	**'Legionella worsleiensis' (GCF_900453045.1)**	14.6	[11.8 - 18.0]	21.5	[19.3 - 23.9]	14.8	[12.4 - 17.7]	2.39
**'Legionella santicrucis' (GCF_001468135.1)**	**'Legionella worsleiensis' (GCF_900453045.1)**	13.5	[10.8 - 16.9]	21.5	[19.2 - 23.9]	13.8	[11.5 - 16.6]	3.76
**'Legionella santicrucis' (GCF_001468135.1)**	**'Legionella nagasakiensis' (GCF_900639915.1)**	13.1	[10.4 - 16.4]	21.5	[19.2 - 23.9]	13.5	[11.1 - 16.2]	4.44
**'Legionella qingyii' (GCF_003184185.1)**	**'Legionella worsleiensis' (GCF_900453045.1)**	13.3	[10.6 - 16.6]	21.5	[19.3 - 24.0]	13.6	[11.3 - 16.4]	2.61

Query	Subject	d_0	C.I. d_0	d_4	C.I. d_4	d_6	C.I. d_6	Diff. G+C Percent
**'Legionella sp. PATHC032' (GCF_026191185.1)**	**'Legionella wadsworthii' (GCF_900452925.1)**	13.4	[10.7 - 16.7]	21.5	[19.2 - 23.9]	13.7	[11.3 - 16.5]	0.05
**'Legionella drancourtii LLAP12' (GCF_000162755.2)**	**'Legionella wadsworthii' (GCF_900452925.1)**	14.0	[11.2 - 17.4]	21.5	[19.3 - 24.0]	14.3	[11.8 - 17.1]	1.16
**'Legionella waltersii' (GCF_900187095.1)**	**'Legionella cherrii' (GCF_900635815.1)**	13.3	[10.6 - 16.6]	21.5	[19.2 - 23.9]	13.7	[11.3 - 16.4]	0.27
**'Legionella bononiensis' (GCF_016786415.1)**	**'Legionella longbeachae' (GCF_019930685.1)**	13.3	[10.5 - 16.6]	21.5	[19.3 - 24.0]	13.6	[11.2 - 16.4]	1.97
**'Legionella norrlandica' (GCF_000770585.1)**	**'Fluoribacter gormanii' (GCF_900156395.1)**	13.3	[10.6 - 16.7]	21.5	[19.2 - 23.9]	13.7	[11.3 - 16.5]	0.61
**'Legionella sp. PATHC032' (GCF_026191185.1)**	**'Legionella taurinensis' (GCF_900452865.1)**	13.0	[10.3 - 16.2]	21.5	[19.3 - 24.0]	13.3	[11.0 - 16.1]	10.03
**'Legionella worsleiensis' (GCF_900453045.1)**	**'Legionella bozemanae' (GCF_900640135.1)**	13.6	[10.9 - 17.0]	21.5	[19.3 - 24.0]	13.9	[11.5 - 16.7]	2.5
**'Legionella wadsworthii' (GCF_900452925.1)**	**'Legionella sainthelensi' (GCF_900637685.1)**	15.1	[12.2 - 18.6]	21.5	[19.3 - 23.9]	15.3	[12.8 - 18.1]	1.02
**'Legionella gratiana' (GCF_900452545.1)**	**'Legionella nagasakiensis' (GCF_900639915.1)**	13.2	[10.5 - 16.5]	21.5	[19.3 - 23.9]	13.5	[11.2 - 16.3]	4.12
**'Legionella longbeachae' (GCF_019930685.1)**	**'Legionella wadsworthii' (GCF_900452925.1)**	15.0	[12.1 - 18.4]	21.5	[19.3 - 24.0]	15.1	[12.7 - 18.0]	1.01
**'Legionella moravica' (GCF_900452715.1)**	**'Legionella bozemanae' (GCF_900640135.1)**	13.4	[10.6 - 16.7]	21.5	[19.2 - 23.9]	13.7	[11.3 - 16.5]	2.21
**'Legionella norrlandica' (GCF_000770585.1)**	**'Legionella bozemanae' (GCF_900640135.1)**	13.3	[10.6 - 16.6]	21.5	[19.2 - 23.9]	13.7	[11.3 - 16.5]	0.44
**'Fluoribacter dumoffii NY 23' (GCF_000236165.1)**	**'Legionella longbeachae' (GCF_019930685.1)**	15.6	[12.7 - 19.1]	21.5	[19.3 - 23.9]	15.7	[13.2 - 18.6]	2.57
**'Legionella resiliens' (GCF_021344005.1)**	**'Legionella sp. PATHC032' (GCF_026191185.1)**	13.5	[10.7 - 16.8]	21.5	[19.3 - 24.0]	13.8	[11.4 - 16.6]	0.13
**'Legionella resiliens' (GCF_021344005.1)**	**'Legionella maioricensis' (GCF_023618015.1)**	13.7	[10.9 - 17.0]	21.5	[19.3 - 24.0]	14.0	[11.6 - 16.8]	1.13
**'Legionella oakridgensis' (GCF_001467925.1)**	**'Legionella sp. PATHC035' (GCF_026191115.1)**	12.9	[10.2 - 16.1]	21.4	[19.2 - 23.9]	13.2	[10.9 - 16.0]	1.4
**'Legionella norrlandica' (GCF_000770585.1)**	**'Legionella cincinnatiensis' (GCF_900452415.1)**	13.6	[10.8 - 16.9]	21.4	[19.2 - 23.9]	13.9	[11.5 - 16.7]	0.6
**'Legionella drancourtii LLAP12' (GCF_000162755.2)**	**'Legionella nagasakiensis' (GCF_900639915.1)**	13.1	[10.4 - 16.4]	21.4	[19.2 - 23.9]	13.4	[11.1 - 16.2]	1.85
**'Legionella drancourtii LLAP12' (GCF_000162755.2)**	**'Legionella sp. PATHC032' (GCF_026191185.1)**	13.3	[10.6 - 16.7]	21.4	[19.2 - 23.8]	13.7	[11.3 - 16.5]	1.22
**'Legionella maioricensis' (GCF_023618015.1)**	**'Legionella nagasakiensis' (GCF_900639915.1)**	12.9	[10.2 - 16.2]	21.4	[19.1 - 23.8]	13.3	[10.9 - 16.0]	1.81
**'Legionella maioricensis' (GCF_023618015.1)**	**'Legionella quateirensis' (GCF_900452695.1)**	16.1	[13.1 - 19.6]	21.4	[19.2 - 23.9]	16.1	[13.6 - 19.0]	0.23
**'Legionella steelei' (GCF_001468005.1)**	**'Legionella worsleiensis' (GCF_900453045.1)**	13.4	[10.7 - 16.8]	21.4	[19.1 - 23.8]	13.7	[11.4 - 16.5]	1.65
**'Legionella septentrionalis' (GCF_003989745.1)**	**'Legionella bononiensis' (GCF_016786415.1)**	13.1	[10.4 - 16.4]	21.4	[19.2 - 23.8]	13.5	[11.1 - 16.2]	3.12
**'Legionella oakridgensis' (GCF_001467925.1)**	**'Legionella quateirensis' (GCF_900452695.1)**	12.8	[10.1 - 16.1]	21.4	[19.1 - 23.8]	13.2	[10.9 - 16.0]	1.77

Query	Subject	d_0	C.I. d_0	d_4	C.I. d_4	d_6	C.I. d_6	Diff. G+C Percent
**'Legionella worsleiensis' (GCF_900453045.1)**	**'Legionella sainthelensi' (GCF_900637685.1)**	13.3	[10.6 - 16.6]	21.4	[19.1 - 23.8]	13.7	[11.3 - 16.4]	3.35
**'Legionella longbeachae' (GCF_019930685.1)**	**'Legionella worsleiensis' (GCF_900453045.1)**	13.4	[10.6 - 16.7]	21.4	[19.2 - 23.8]	13.7	[11.3 - 16.5]	3.34
**'Legionella maioricensis' (GCF_023618015.1)**	**'Legionella sp. PATHC032' (GCF_026191185.1)**	14.6	[11.8 - 18.1]	21.4	[19.2 - 23.9]	14.8	[12.4 - 17.7]	1.26
**'Legionella anisa' (GCA_019930885.1)**	**'Legionella taurinensis' (GCF_900452865.1)**	13.2	[10.5 - 16.5]	21.4	[19.2 - 23.9]	13.5	[11.2 - 16.3]	9.77
**'Legionella saoudiensis' (GCF_001465875.1)**	**'Legionella worsleiensis' (GCF_900453045.1)**	13.3	[10.5 - 16.6]	21.3	[19.0 - 23.7]	13.6	[11.2 - 16.4]	0.99
**'Legionella tucsonensis' (GCF_001468035.1)**	**'Legionella pneumophila' (GCF_001941585.1)**	13.6	[10.9 - 17.0]	21.3	[19.1 - 23.8]	13.9	[11.5 - 16.8]	0.92
**'Legionella oakridgensis' (GCF_001467925.1)**	**'Legionella wadsworthii' (GCF_900452925.1)**	12.9	[10.2 - 16.2]	21.3	[19.1 - 23.8]	13.3	[10.9 - 16.1]	2.75
**'Legionella antarctica' (GCF_011764505.1)**	**'Fluoribacter gormanii' (GCF_900156395.1)**	13.6	[10.8 - 16.9]	21.3	[19.0 - 23.7]	13.9	[11.5 - 16.7]	1.06
**'Legionella waltersii' (GCF_900187095.1)**	**'Legionella worsleiensis' (GCF_900453045.1)**	13.6	[10.9 - 17.0]	21.3	[19.1 - 23.7]	13.9	[11.5 - 16.7]	1.24
**'Legionella norrlandica' (GCF_000770585.1)**	**'Legionella resiliens' (GCF_021344005.1)**	13.4	[10.7 - 16.8]	21.3	[19.0 - 23.7]	13.8	[11.4 - 16.6]	0.69
**'Legionella bononiensis' (GCF_016786415.1)**	**'Legionella cherrii' (GCF_900635815.1)**	13.6	[10.9 - 17.0]	21.3	[19.1 - 23.7]	13.9	[11.5 - 16.8]	0.14
**'Legionella moravica' (GCF_900452715.1)**	**'Legionella rubrilucens' (GCF_900640015.1)**	13.2	[10.5 - 16.6]	21.3	[19.1 - 23.8]	13.6	[11.2 - 16.4]	7.41
**'Legionella drancourtii LLAP12' (GCF_000162755.2)**	**'Legionella sainthelensi' (GCF_900637685.1)**	14.3	[11.4 - 17.7]	21.3	[19.1 - 23.8]	14.5	[12.1 - 17.3]	2.19
**'Legionella norrlandica' (GCF_000770585.1)**	**'Legionella sp. PATHC038' (GCF_026191355.1)**	13.4	[10.6 - 16.7]	21.2	[18.9 - 23.6]	13.7	[11.3 - 16.5]	1.35
**'Legionella steelei' (GCF_001468005.1)**	**'Legionella moravica' (GCF_900452715.1)**	13.8	[11.0 - 17.1]	21.2	[19.0 - 23.6]	14.1	[11.7 - 16.9]	1.36
**'Legionella bononiensis' (GCF_016786415.1)**	**'Legionella resiliens' (GCF_021344005.1)**	13.6	[10.8 - 16.9]	21.2	[18.9 - 23.6]	13.9	[11.5 - 16.7]	0.89
**'Legionella norrlandica' (GCF_000770585.1)**	**'Legionella bononiensis' (GCF_016786415.1)**	14.2	[11.4 - 17.6]	21.2	[19.0 - 23.7]	14.4	[12.0 - 17.3]	1.57
**'Legionella fallonii LLAP-10' (GCF_000953135.1)**	**'Legionella bononiensis' (GCF_016786415.1)**	14.6	[11.7 - 18.0]	21.2	[19.0 - 23.7]	14.8	[12.3 - 17.6]	0.72
**'Legionella shakespearei DSM 23087' (GCF_000373765.1)**	**'Legionella santicrucis' (GCF_001468135.1)**	13.3	[10.6 - 16.6]	21.2	[18.9 - 23.6]	13.6	[11.3 - 16.4]	5.04
**'Legionella saoudiensis' (GCF_001465875.1)**	**'Legionella waltersii' (GCF_900187095.1)**	13.4	[10.6 - 16.7]	21.1	[18.8 - 23.5]	13.7	[11.3 - 16.5]	0.25
**'Legionella antarctica' (GCF_011764505.1)**	**'Legionella rubrilucens' (GCF_900640015.1)**	12.8	[10.2 - 16.1]	21.1	[18.9 - 23.5]	13.2	[10.9 - 16.0]	8.39
**'Legionella steelei' (GCF_001468005.1)**	**'Legionella pneumophila' (GCF_001941585.1)**	13.6	[10.8 - 16.9]	21.1	[18.8 - 23.5]	13.9	[11.5 - 16.7]	0.44
**'Legionella norrlandica' (GCF_000770585.1)**	**'Legionella waltersii' (GCF_900187095.1)**	14.1	[11.3 - 17.5]	21.1	[18.9 - 23.5]	14.4	[12.0 - 17.2]	1.7
**'Legionella qingyii' (GCF_003184185.1)**	**'Legionella antarctica' (GCF_011764505.1)**	13.4	[10.6 - 16.7]	21.1	[18.9 - 23.6]	13.7	[11.3 - 16.5]	1.34
**'Legionella antarctica' (GCF_011764505.1)**	**'Legionella quateirensis' (GCF_900452695.1)**	15.1	[12.2 - 18.5]	21.1	[18.8 - 23.5]	15.2	[12.7 - 18.1]	0.08
**'Legionella anisa' (GCA_019930885.1)**	**'Legionella shakespearei DSM 23087' (GCF_000373765.1)**	13.5	[10.8 - 16.9]	21.1	[18.8 - 23.5]	13.8	[11.4 - 16.6]	3.41

Query	Subject	d_0	C.I. d_0	d_4	C.I. d_4	d_6	C.I. d_6	Diff. G+C Percent
**'Legionella drancourtii LLAP12' (GCF_000162755.2)**	**'Legionella rubrilucens' (GCF_900640015.1)**	13.0	[10.3 - 16.3]	21.1	[18.9 - 23.6]	13.4	[11.0 - 16.2]	8.29
**'Legionella shakespearei DSM 23087' (GCF_000373765.1)**	**'Legionella bononiensis' (GCF_016786415.1)**	15.3	[12.4 - 18.7]	21.1	[18.9 - 23.6]	15.4	[12.9 - 18.3]	2.65
**'Legionella norrlandica' (GCF_000770585.1)**	**'Legionella longbeachae' (GCF_019930685.1)**	13.4	[10.7 - 16.8]	21.1	[18.9 - 23.6]	13.8	[11.4 - 16.6]	0.4
**'Legionella qingyii' (GCF_003184185.1)**	**'Legionella quateirensis' (GCF_900452695.1)**	13.6	[10.9 - 17.0]	21.1	[18.9 - 23.5]	13.9	[11.5 - 16.7]	1.26
**'Legionella anisa' (GCA_019930885.1)**	**'Legionella norrlandica' (GCF_000770585.1)**	13.5	[10.7 - 16.8]	21.1	[18.9 - 23.5]	13.8	[11.4 - 16.6]	0.81
**'Legionella santicrucis' (GCF_001468135.1)**	**'Legionella septentrionalis' (GCF_003989745.1)**	13.0	[10.3 - 16.3]	21.1	[18.9 - 23.6]	13.4	[11.0 - 16.1]	5.51
**'Legionella norrlandica' (GCF_000770585.1)**	**'Legionella sp. PATHC035' (GCF_026191115.1)**	13.3	[10.6 - 16.6]	21.0	[18.8 - 23.5]	13.6	[11.3 - 16.4]	1.96
**'Legionella sp. PATHC032' (GCF_026191185.1)**	**'Legionella waltersii' (GCF_900187095.1)**	14.3	[11.5 - 17.7]	21.0	[18.7 - 23.4]	14.5	[12.1 - 17.4]	1.15
**'Legionella fallonii LLAP-10' (GCF_000953135.1)**	**'Legionella moravica' (GCF_900452715.1)**	14.4	[11.6 - 17.8]	21.0	[18.8 - 23.4]	14.6	[12.2 - 17.5]	1.8
**'Legionella shakespearei DSM 23087' (GCF_000373765.1)**	**'Fluoribacter gormanii' (GCF_900156395.1)**	13.6	[10.8 - 17.0]	21.0	[18.7 - 23.4]	13.9	[11.5 - 16.7]	3.61
**'Legionella drancourtii LLAP12' (GCF_000162755.2)**	**'Legionella norrlandica' (GCF_000770585.1)**	13.2	[10.5 - 16.6]	21.0	[18.8 - 23.5]	13.6	[11.2 - 16.4]	1.77
**'Legionella oakridgensis' (GCF_001467925.1)**	**'Legionella sp. PATHC038' (GCF_026191355.1)**	12.8	[10.1 - 16.1]	21.0	[18.8 - 23.4]	13.2	[10.8 - 16.0]	2.01
**'Legionella saoudiensis' (GCF_001465875.1)**	**'Legionella moravica' (GCF_900452715.1)**	13.4	[10.6 - 16.7]	21.0	[18.7 - 23.4]	13.7	[11.3 - 16.5]	0.7
**'Legionella saoudiensis' (GCF_001465875.1)**	**'Legionella resiliens' (GCF_021344005.1)**	13.9	[11.1 - 17.3]	21.0	[18.7 - 23.4]	14.2	[11.8 - 17.0]	1.27
**'Legionella norrlandica' (GCF_000770585.1)**	**'Legionella moravica' (GCF_900452715.1)**	14.1	[11.3 - 17.5]	21.0	[18.7 - 23.4]	14.4	[11.9 - 17.2]	2.65
**'Legionella tucsonensis' (GCF_001468035.1)**	**'Legionella nagasakiensis' (GCF_900639915.1)**	12.9	[10.2 - 16.2]	21.0	[18.8 - 23.5]	13.3	[10.9 - 16.0]	3.68
**'Legionella oakridgensis' (GCF_001467925.1)**	**'Legionella antarctica' (GCF_011764505.1)**	12.8	[10.1 - 16.1]	21.0	[18.8 - 23.4]	13.2	[10.9 - 16.0]	1.69
**'Legionella saoudiensis' (GCF_001465875.1)**	**'Legionella tucsonensis' (GCF_001468035.1)**	14.1	[11.3 - 17.4]	21.0	[18.8 - 23.4]	14.3	[11.9 - 17.2]	2.02
**'Legionella saoudiensis' (GCF_001465875.1)**	**'Legionella taurinensis' (GCF_900452865.1)**	12.8	[10.1 - 16.1]	21.0	[18.8 - 23.5]	13.2	[10.8 - 15.9]	8.63
**'Legionella worsleiensis' (GCF_900453045.1)**	**'Legionella cherrii' (GCF_900635815.1)**	13.5	[10.8 - 16.9]	20.9	[18.7 - 23.4]	13.8	[11.4 - 16.6]	1.51
**'Legionella resiliens' (GCF_021344005.1)**	**'Legionella taurinensis' (GCF_900452865.1)**	12.8	[10.1 - 16.1]	20.9	[18.7 - 23.3]	13.2	[10.9 - 16.0]	9.9
**'Legionella tucsonensis' (GCF_001468035.1)**	**'Legionella moravica' (GCF_900452715.1)**	13.7	[10.9 - 17.0]	20.9	[18.7 - 23.3]	14.0	[11.6 - 16.8]	2.71
**'Legionella taurinensis' (GCF_900452865.1)**	**'Legionella bozemanae' (GCF_900640135.1)**	12.9	[10.2 - 16.2]	20.9	[18.7 - 23.3]	13.3	[10.9 - 16.1]	10.14
**'Legionella fallonii LLAP-10' (GCF_000953135.1)**	**'Legionella nagasakiensis' (GCF_900639915.1)**	12.9	[10.2 - 16.2]	20.9	[18.7 - 23.3]	13.3	[10.9 - 16.0]	2.77

Query	Subject	d_0	C.I. d_0	d_4	C.I. d_4	d_6	C.I. d_6	Diff. G+C Percent
**'Legionella cincinnatiensis' (GCF_900452415.1)**	**'Legionella worsleiensis' (GCF_900453045.1)**	13.5	[10.7 - 16.8]	20.9	[18.7 - 23.3]	13.8	[11.4 - 16.6]	3.55
**'Legionella oakridgensis' (GCF_001467925.1)**	**'Legionella waltersii' (GCF_900187095.1)**	12.8	[10.1 - 16.1]	20.9	[18.7 - 23.3]	13.2	[10.9 - 16.0]	1.66
**'Legionella shakespearei DSM 23087' (GCF_000373765.1)**	**'Legionella cincinnatiensis' (GCF_900452415.1)**	13.3	[10.6 - 16.6]	20.9	[18.6 - 23.3]	13.6	[11.2 - 16.4]	4.83
**'Fluoribacter gormanii' (GCF_900156395.1)**	**'Legionella worsleiensis' (GCF_900453045.1)**	13.4	[10.7 - 16.8]	20.8	[18.6 - 23.2]	13.8	[11.4 - 16.6]	2.33
**'Legionella norrlandica' (GCF_000770585.1)**	**'Legionella qingyii' (GCF_003184185.1)**	13.4	[10.7 - 16.7]	20.8	[18.5 - 23.2]	13.7	[11.3 - 16.5]	0.33
**'Legionella tucsonensis' (GCF_001468035.1)**	**'Legionella quateirensis' (GCF_900452695.1)**	13.7	[10.9 - 17.1]	20.8	[18.6 - 23.3]	14.0	[11.6 - 16.8]	1.65
**'Legionella oakridgensis' (GCF_001467925.1)**	**'Legionella steelei' (GCF_001468005.1)**	12.8	[10.1 - 16.1]	20.8	[18.5 - 23.2]	13.2	[10.8 - 15.9]	2.07
**'Legionella fallonii LLAP-10' (GCF_000953135.1)**	**'Legionella quateirensis' (GCF_900452695.1)**	14.6	[11.7 - 18.0]	20.8	[18.6 - 23.2]	14.8	[12.3 - 17.6]	0.74
**'Legionella waltersii' (GCF_900187095.1)**	**'Legionella quateirensis' (GCF_900452695.1)**	14.1	[11.3 - 17.5]	20.8	[18.5 - 23.2]	14.3	[11.9 - 17.2]	0.11
**'Legionella sp. PATHC039' (GCF_026191275.1)**	**'Legionella moravica' (GCF_900452715.1)**	14.8	[11.9 - 18.2]	20.8	[18.6 - 23.3]	14.9	[12.5 - 17.8]	1.99
**'Fluoribacter gormanii' (GCF_900156395.1)**	**'Legionella waltersii' (GCF_900187095.1)**	13.5	[10.7 - 16.8]	20.8	[18.6 - 23.2]	13.8	[11.4 - 16.6]	1.09
**'Legionella antarctica' (GCF_011764505.1)**	**'Legionella nagasakiensis' (GCF_900639915.1)**	12.9	[10.2 - 16.2]	20.8	[18.5 - 23.2]	13.3	[10.9 - 16.0]	1.95
**'Legionella anisa' (GCA_019930885.1)**	**'Legionella septentrionalis' (GCF_003989745.1)**	13.0	[10.3 - 16.3]	20.8	[18.5 - 23.2]	13.3	[11.0 - 16.1]	3.88
**'Legionella antarctica' (GCF_011764505.1)**	**'Legionella sp. PATHC039' (GCF_026191275.1)**	14.2	[11.3 - 17.5]	20.8	[18.6 - 23.3]	14.4	[12.0 - 17.2]	1.0
**'Legionella oakridgensis' (GCF_001467925.1)**	**'Legionella longbeachae' (GCF_019930685.1)**	13.0	[10.2 - 16.2]	20.7	[18.5 - 23.1]	13.3	[11.0 - 16.1]	3.76
**'Legionella quateirensis' (GCF_900452695.1)**	**'Legionella nagasakiensis' (GCF_900639915.1)**	13.0	[10.3 - 16.3]	20.7	[18.5 - 23.1]	13.4	[11.0 - 16.1]	2.03
**'Legionella septentrionalis' (GCF_003989745.1)**	**'Legionella cincinnatiensis' (GCF_900452415.1)**	12.9	[10.2 - 16.2]	20.7	[18.5 - 23.1]	13.3	[10.9 - 16.1]	5.29
**'Legionella pneumophila' (GCF_001941585.1)**	**'Legionella antarctica' (GCF_011764505.1)**	14.0	[11.2 - 17.4]	20.7	[18.5 - 23.1]	14.3	[11.9 - 17.1]	0.81
**'Legionella oakridgensis' (GCF_001467925.1)**	**'Legionella bozemanae' (GCF_900640135.1)**	12.9	[10.2 - 16.2]	20.7	[18.5 - 23.1]	13.3	[11.0 - 16.1]	2.92
**'Legionella tucsonensis' (GCF_001468035.1)**	**'Legionella maioricensis' (GCF_023618015.1)**	13.9	[11.1 - 17.3]	20.7	[18.5 - 23.1]	14.2	[11.8 - 17.0]	1.88
**'Legionella antarctica' (GCF_011764505.1)**	**'Legionella bononiensis' (GCF_016786415.1)**	15.1	[12.2 - 18.6]	20.7	[18.4 - 23.1]	15.2	[12.7 - 18.1]	0.1
**'Legionella pneumophila' (GCF_001941585.1)**	**'Legionella waltersii' (GCF_900187095.1)**	14.6	[11.8 - 18.0]	20.7	[18.5 - 23.1]	14.8	[12.3 - 17.7]	0.85
**'Legionella septentrionalis' (GCF_003989745.1)**	**'Legionella wadsworthii' (GCF_900452925.1)**	12.9	[10.2 - 16.1]	20.7	[18.4 - 23.1]	13.2	[10.9 - 16.0]	4.07
**'Fluoribacter gormanii' (GCF_900156395.1)**	**'Legionella nagasakiensis' (GCF_900639915.1)**	13.0	[10.3 - 16.3]	20.7	[18.4 - 23.1]	13.3	[11.0 - 16.1]	3.01

Query	Subject	d_0	C.I. d_0	d_4	C.I. d_4	d_6	C.I. d_6	Diff. G+C Percent
**'Legionella oakridgensis' (GCF_001467925.1)**	**'Legionella tucsonensis' (GCF_001468035.1)**	12.9	[10.2 - 16.1]	20.7	[18.5 - 23.1]	13.2	[10.9 - 16.0]	3.42
**'Legionella norrlandica' (GCF_000770585.1)**	**'Legionella fallonii LLAP-10' (GCF_000953135.1)**	13.5	[10.8 - 16.9]	20.7	[18.5 - 23.1]	13.8	[11.4 - 16.6]	0.85
**'Legionella oakridgensis' (GCF_001467925.1)**	**'Fluoribacter gormanii' (GCF_900156395.1)**	12.8	[10.1 - 16.1]	20.7	[18.5 - 23.1]	13.2	[10.8 - 15.9]	2.75
**'Legionella shakespearei DSM 23087' (GCF_000373765.1)**	**'Legionella steigerwaltii' (GCF_900452835.1)**	13.3	[10.6 - 16.7]	20.7	[18.5 - 23.1]	13.7	[11.3 - 16.4]	3.38
**'Legionella pneumophila' (GCF_001941585.1)**	**'Legionella moravica' (GCF_900452715.1)**	14.8	[11.9 - 18.2]	20.7	[18.4 - 23.1]	15.0	[12.5 - 17.8]	1.8
**'Legionella septentrionalis' (GCF_003989745.1)**	**'Legionella antarctica' (GCF_011764505.1)**	12.8	[10.1 - 16.1]	20.7	[18.5 - 23.1]	13.2	[10.9 - 16.0]	3.02
**'Legionella fallonii LLAP-10' (GCF_000953135.1)**	**'Legionella oakridgensis' (GCF_001467925.1)**	12.9	[10.2 - 16.2]	20.7	[18.5 - 23.1]	13.3	[10.9 - 16.0]	2.51
**'Legionella steelei' (GCF_001468005.1)**	**'Legionella taurinensis' (GCF_900452865.1)**	12.9	[10.2 - 16.2]	20.6	[18.4 - 23.0]	13.2	[10.9 - 16.0]	9.29
**'Legionella longbeachae' (GCF_019930685.1)**	**'Legionella rubrilucens' (GCF_900640015.1)**	12.9	[10.2 - 16.2]	20.6	[18.3 - 23.0]	13.3	[11.0 - 16.1]	10.47
**'Legionella norrlandica' (GCF_000770585.1)**	**'Legionella cherrii' (GCF_900635815.1)**	13.5	[10.7 - 16.8]	20.6	[18.4 - 23.0]	13.8	[11.4 - 16.6]	1.43
**'Legionella shakespearei DSM 23087' (GCF_000373765.1)**	**'Legionella quateirensis' (GCF_900452695.1)**	15.4	[12.5 - 18.8]	20.6	[18.4 - 23.1]	15.4	[12.9 - 18.3]	2.64
**'Legionella antarctica' (GCF_011764505.1)**	**'Legionella sp. PATHC032' (GCF_026191185.1)**	14.0	[11.2 - 17.4]	20.6	[18.4 - 23.0]	14.3	[11.8 - 17.1]	1.11
**'Legionella shakespearei DSM 23087' (GCF_000373765.1)**	**'Legionella gratiana' (GCF_900452545.1)**	13.6	[10.8 - 16.9]	20.6	[18.4 - 23.0]	13.9	[11.5 - 16.7]	4.72
**'Legionella tucsonensis' (GCF_001468035.1)**	**'Legionella waltersii' (GCF_900187095.1)**	13.4	[10.6 - 16.7]	20.6	[18.4 - 23.0]	13.7	[11.3 - 16.5]	1.76
**'Legionella tucsonensis' (GCF_001468035.1)**	**'Legionella bononiensis' (GCF_016786415.1)**	13.6	[10.8 - 16.9]	20.6	[18.3 - 23.0]	13.9	[11.5 - 16.7]	1.63
**'Legionella norrlandica' (GCF_000770585.1)**	**'Legionella antarctica' (GCF_011764505.1)**	13.8	[11.1 - 17.2]	20.6	[18.4 - 23.0]	14.1	[11.7 - 16.9]	1.67
**'Legionella norrlandica' (GCF_000770585.1)**	**'Legionella wadsworthii' (GCF_900452925.1)**	13.3	[10.5 - 16.6]	20.6	[18.3 - 23.0]	13.6	[11.2 - 16.4]	0.61
**'Legionella sp. PATHC032' (GCF_026191185.1)**	**'Legionella quateirensis' (GCF_900452695.1)**	14.9	[12.1 - 18.4]	20.6	[18.4 - 23.0]	15.1	[12.6 - 18.0]	1.03
**'Legionella taurinensis' (GCF_900452865.1)**	**'Legionella worsleiensis' (GCF_900453045.1)**	13.1	[10.3 - 16.4]	20.6	[18.4 - 23.1]	13.4	[11.0 - 16.2]	7.64
**'Legionella tucsonensis' (GCF_001468035.1)**	**'Legionella worsleiensis' (GCF_900453045.1)**	13.6	[10.8 - 16.9]	20.5	[18.2 - 22.9]	13.9	[11.5 - 16.7]	3.0
**'Legionella quateirensis' (GCF_900452695.1)**	**'Legionella rubrilucens' (GCF_900640015.1)**	13.0	[10.3 - 16.3]	20.5	[18.2 - 22.9]	13.4	[11.0 - 16.1]	8.47
**'Legionella oakridgensis' (GCF_001467925.1)**	**'Legionella sp. PATHC039' (GCF_026191275.1)**	13.1	[10.4 - 16.4]	20.5	[18.2 - 22.9]	13.5	[11.1 - 16.2]	2.7
**'Legionella waltersii' (GCF_900187095.1)**	**'Legionella bozemanae' (GCF_900640135.1)**	13.4	[10.6 - 16.7]	20.5	[18.2 - 22.9]	13.7	[11.3 - 16.5]	1.26
**'Legionella shakespearei DSM 23087' (GCF_000373765.1)**	**'Legionella qingyii' (GCF_003184185.1)**	13.5	[10.7 - 16.8]	20.5	[18.3 - 22.9]	13.8	[11.4 - 16.6]	3.89
**'Legionella oakridgensis' (GCF_001467925.1)**	**'Legionella maioricensis' (GCF_023618015.1)**	12.9	[10.2 - 16.2]	20.5	[18.3 - 22.9]	13.3	[10.9 - 16.0]	1.55

Query	Subject	d_0	C.I. d_0	d_4	C.I. d_4	d_6	C.I. d_6	Diff. G+C Percent
**'Legionella maioricensis' (GCF_023618015.1)**	**'Legionella moravica' (GCF_900452715.1)**	15.8	[12.8 - 19.2]	20.5	[18.3 - 22.9]	15.8	[13.3 - 18.7]	0.84
**'Legionella saoudiensis' (GCF_001465875.1)**	**'Legionella nagasakiensis' (GCF_900639915.1)**	13.0	[10.3 - 16.3]	20.5	[18.3 - 22.9]	13.3	[11.0 - 16.1]	1.67
**'Legionella shakespearei DSM 23087' (GCF_000373765.1)**	**'Legionella saoudiensis' (GCF_001465875.1)**	13.6	[10.9 - 17.0]	20.4	[18.2 - 22.9]	13.9	[11.5 - 16.7]	2.27
**'Legionella septentrionalis' (GCF_003989745.1)**	**'Legionella sp. PATHC035' (GCF_026191115.1)**	12.9	[10.2 - 16.2]	20.4	[18.2 - 22.8]	13.3	[10.9 - 16.0]	2.72
**'Legionella norrlandica' (GCF_000770585.1)**	**'Legionella saoudiensis' (GCF_001465875.1)**	13.2	[10.5 - 16.5]	20.4	[18.2 - 22.9]	13.5	[11.2 - 16.3]	1.96
**'Legionella shakespearei DSM 23087' (GCF_000373765.1)**	**'Legionella resiliens' (GCF_021344005.1)**	13.4	[10.6 - 16.7]	20.4	[18.2 - 22.8]	13.7	[11.3 - 16.5]	3.54
**'Fluoribacter gormanii' (GCF_900156395.1)**	**'Legionella moravica' (GCF_900452715.1)**	13.6	[10.8 - 16.9]	20.4	[18.2 - 22.8]	13.9	[11.5 - 16.7]	2.04
**'Legionella sainthelensi' (GCF_900637685.1)**	**'Legionella nagasakiensis' (GCF_900639915.1)**	13.2	[10.4 - 16.5]	20.4	[18.2 - 22.8]	13.5	[11.1 - 16.3]	4.04
**'Legionella saoudiensis' (GCF_001465875.1)**	**'Legionella antarctica' (GCF_011764505.1)**	13.5	[10.7 - 16.8]	20.4	[18.2 - 22.8]	13.8	[11.4 - 16.6]	0.29
**'Legionella oakridgensis' (GCF_001467925.1)**	**'Legionella qingyii' (GCF_003184185.1)**	12.8	[10.1 - 16.1]	20.4	[18.1 - 22.8]	13.2	[10.8 - 15.9]	3.03
**'Legionella drancourtii LLAP12' (GCF_000162755.2)**	**'Legionella oakridgensis' (GCF_001467925.1)**	12.9	[10.2 - 16.2]	20.4	[18.2 - 22.8]	13.2	[10.9 - 16.0]	1.59
**'Legionella oakridgensis' (GCF_001467925.1)**	**'Legionella santicrucis' (GCF_001468135.1)**	12.9	[10.2 - 16.2]	20.4	[18.2 - 22.8]	13.3	[11.0 - 16.1]	4.18
**'Legionella cherrii' (GCF_900635815.1)**	**'Legionella rubrilucens' (GCF_900640015.1)**	12.8	[10.1 - 16.1]	20.3	[18.1 - 22.7]	13.2	[10.9 - 16.0]	8.64
**'Fluoribacter dumoffii NY 23' (GCF_000236165.1)**	**'Legionella moravica' (GCF_900452715.1)**	13.8	[11.0 - 17.2]	20.3	[18.1 - 22.7]	14.1	[11.7 - 16.9]	0.49
**'Legionella quateirensis' (GCF_900452695.1)**	**'Legionella taurinensis' (GCF_900452865.1)**	13.1	[10.3 - 16.4]	20.3	[18.1 - 22.7]	13.4	[11.1 - 16.2]	8.99
**'Legionella moravica' (GCF_900452715.1)**	**'Legionella nagasakiensis' (GCF_900639915.1)**	13.1	[10.4 - 16.4]	20.3	[18.1 - 22.7]	13.4	[11.1 - 16.2]	0.97
**'Legionella fallonii LLAP-10' (GCF_000953135.1)**	**'Legionella rubrilucens' (GCF_900640015.1)**	12.9	[10.2 - 16.2]	20.3	[18.1 - 22.7]	13.3	[10.9 - 16.0]	9.21
**'Legionella sp. PATHC039' (GCF_026191275.1)**	**'Legionella nagasakiensis' (GCF_900639915.1)**	13.1	[10.4 - 16.4]	20.3	[18.1 - 22.7]	13.5	[11.1 - 16.2]	2.96
**'Legionella shakespearei DSM 23087' (GCF_000373765.1)**	**'Legionella bozemanae' (GCF_900640135.1)**	13.5	[10.7 - 16.8]	20.3	[18.1 - 22.7]	13.8	[11.4 - 16.6]	3.78
**'Legionella maioricensis' (GCF_023618015.1)**	**'Legionella waltersii' (GCF_900187095.1)**	13.9	[11.1 - 17.3]	20.3	[18.1 - 22.7]	14.2	[11.8 - 17.0]	0.11
**'Legionella antarctica' (GCF_011764505.1)**	**'Legionella moravica' (GCF_900452715.1)**	15.1	[12.2 - 18.5]	20.3	[18.1 - 22.7]	15.2	[12.7 - 18.1]	0.98
**'Legionella norrlandica' (GCF_000770585.1)**	**'Legionella steelei' (GCF_001468005.1)**	13.5	[10.8 - 16.9]	20.2	[18.0 - 22.6]	13.8	[11.4 - 16.6]	1.3
**'Legionella qingyii' (GCF_003184185.1)**	**'Legionella nagasakiensis' (GCF_900639915.1)**	12.9	[10.2 - 16.2]	20.2	[18.0 - 22.6]	13.2	[10.9 - 16.0]	3.29
**'Legionella septentrionalis' (GCF_003989745.1)**	**'Legionella resiliens' (GCF_021344005.1)**	12.9	[10.2 - 16.2]	20.2	[18.0 - 22.6]	13.3	[10.9 - 16.0]	4.0

Query	Subject	d_0	C.I. d_0	d_4	C.I. d_4	d_6	C.I. d_6	Diff. G+C Percent
**'Legionella resiliens' (GCF_021344005.1)**	**'Legionella worsleiensis' (GCF_900453045.1)**	13.4	[10.7 - 16.8]	20.2	[18.0 - 22.7]	13.7	[11.4 - 16.5]	2.26
**'Legionella bononiensis' (GCF_016786415.1)**	**'Legionella sp. PATHC032' (GCF_026191185.1)**	15.0	[12.1 - 18.4]	20.2	[18.0 - 22.6]	15.1	[12.6 - 18.0]	1.01
**'Legionella septentrionalis' (GCF_003989745.1)**	**'Legionella longbeachae' (GCF_019930685.1)**	12.8	[10.1 - 16.1]	20.2	[18.0 - 22.6]	13.2	[10.8 - 15.9]	5.09
**'Legionella tucsonensis' (GCF_001468035.1)**	**'Legionella septentrionalis' (GCF_003989745.1)**	12.8	[10.1 - 16.1]	20.2	[18.0 - 22.6]	13.2	[10.8 - 16.0]	4.75
**'Legionella waltersii' (GCF_900187095.1)**	**'Legionella nagasakiensis' (GCF_900639915.1)**	13.2	[10.5 - 16.6]	20.2	[18.0 - 22.6]	13.6	[11.2 - 16.4]	1.92
**'Legionella shakespearei DSM 23087' (GCF_000373765.1)**	**'Legionella cherrii' (GCF_900635815.1)**	13.5	[10.7 - 16.8]	20.1	[17.9 - 22.5]	13.8	[11.4 - 16.6]	2.8
**'Legionella drancourtii LLAP12' (GCF_000162755.2)**	**'Legionella shakespearei DSM 23087' (GCF_000373765.1)**	13.8	[11.0 - 17.2]	20.1	[17.9 - 22.5]	14.1	[11.7 - 16.9]	2.45
**'Legionella cincinnatiensis' (GCF_900452415.1)**	**'Legionella rubrilucens' (GCF_900640015.1)**	13.0	[10.3 - 16.3]	20.1	[17.9 - 22.5]	13.4	[11.0 - 16.1]	10.67
**'Legionella antarctica' (GCF_011764505.1)**	**'Legionella waltersii' (GCF_900187095.1)**	13.7	[10.9 - 17.1]	20.1	[17.8 - 22.5]	14.0	[11.6 - 16.8]	0.03
**'Legionella septentrionalis' (GCF_003989745.1)**	**'Legionella steigerwaltii' (GCF_900452835.1)**	12.8	[10.2 - 16.1]	20.1	[17.9 - 22.5]	13.2	[10.9 - 16.0]	3.84
**'Legionella oakridgensis' (GCF_001467925.1)**	**'Legionella worsleiensis' (GCF_900453045.1)**	12.8	[10.1 - 16.1]	20.1	[17.9 - 22.5]	13.2	[10.8 - 15.9]	0.42
**'Legionella oakridgensis' (GCF_001467925.1)**	**'Legionella cincinnatiensis' (GCF_900452415.1)**	13.0	[10.3 - 16.3]	20.1	[17.9 - 22.5]	13.3	[11.0 - 16.1]	3.97
**'Legionella norrlandica' (GCF_000770585.1)**	**'Legionella maioricensis' (GCF_023618015.1)**	13.9	[11.1 - 17.2]	20.1	[17.9 - 22.5]	14.1	[11.7 - 16.9]	1.82
**'Legionella shakespearei DSM 23087' (GCF_000373765.1)**	**'Legionella sp. PATHC035' (GCF_026191115.1)**	13.3	[10.6 - 16.7]	20.1	[17.9 - 22.6]	13.7	[11.3 - 16.5]	2.26
**'Legionella sp. PATHC038' (GCF_026191355.1)**	**'Legionella rubrilucens' (GCF_900640015.1)**	12.9	[10.2 - 16.2]	20.0	[17.8 - 22.4]	13.2	[10.9 - 16.0]	8.71
**'Legionella sp. PATHC032' (GCF_026191185.1)**	**'Legionella moravica' (GCF_900452715.1)**	14.4	[11.6 - 17.8]	20.0	[17.7 - 22.4]	14.6	[12.1 - 17.4]	2.1
**'Legionella sp. PATHC035' (GCF_026191115.1)**	**'Legionella rubrilucens' (GCF_900640015.1)**	12.8	[10.2 - 16.1]	20.0	[17.8 - 22.5]	13.2	[10.9 - 16.0]	8.1
**'Legionella resiliens' (GCF_021344005.1)**	**'Legionella nagasakiensis' (GCF_900639915.1)**	12.8	[10.1 - 16.1]	20.0	[17.8 - 22.4]	13.2	[10.9 - 16.0]	2.94
**'Legionella pneumophila' (GCF_001941585.1)**	**'Legionella quateirensis' (GCF_900452695.1)**	14.7	[11.9 - 18.1]	20.0	[17.8 - 22.4]	14.9	[12.4 - 17.7]	0.73
**'Legionella shakespearei DSM 23087' (GCF_000373765.1)**	**'Legionella tucsonensis' (GCF_001468035.1)**	13.6	[10.8 - 16.9]	20.0	[17.8 - 22.4]	13.9	[11.5 - 16.7]	4.29
**'Legionella saoudiensis' (GCF_001465875.1)**	**'Legionella oakridgensis' (GCF_001467925.1)**	12.9	[10.2 - 16.2]	20.0	[17.8 - 22.4]	13.3	[10.9 - 16.1]	1.41
**'Legionella septentrionalis' (GCF_003989745.1)**	**'Legionella quateirensis' (GCF_900452695.1)**	13.0	[10.3 - 16.3]	19.9	[17.7 - 22.3]	13.3	[11.0 - 16.1]	3.1

Query	Subject	d_0	C.I. d_0	d_4	C.I. d_4	d_6	C.I. d_6	Diff. G+C Percent
**'Legionella shakespearei DSM 23087' (GCF_000373765.1)**	**'Legionella moravica' (GCF_900452715.1)**	15.3	[12.4 - 18.7]	19.9	[17.7 - 22.3]	15.3	[12.8 - 18.2]	1.57
**'Legionella shakespearei DSM 23087' (GCF_000373765.1)**	**'Legionella wadsworthii' (GCF_900452925.1)**	13.4	[10.6 - 16.7]	19.9	[17.7 - 22.3]	13.7	[11.3 - 16.5]	3.61
**'Legionella shakespearei DSM 23087' (GCF_000373765.1)**	**'Legionella sp. PATHC038' (GCF_026191355.1)**	13.4	[10.6 - 16.7]	19.9	[17.7 - 22.3]	13.7	[11.3 - 16.5]	2.87
**'Legionella sp. PATHC032' (GCF_026191185.1)**	**'Legionella nagasakiensis' (GCF_900639915.1)**	13.2	[10.4 - 16.5]	19.9	[17.7 - 22.3]	13.5	[11.1 - 16.3]	3.07
**'Legionella norrlandica' (GCF_000770585.1)**	**'Legionella quateirensis' (GCF_900452695.1)**	14.1	[11.3 - 17.5]	19.9	[17.7 - 22.4]	14.3	[11.9 - 17.2]	1.59
**'Legionella shakespearei DSM 23087' (GCF_000373765.1)**	**'Legionella sp. PATHC032' (GCF_026191185.1)**	14.4	[11.5 - 17.8]	19.9	[17.7 - 22.3]	14.6	[12.1 - 17.4]	3.67
**'Legionella maioricensis' (GCF_023618015.1)**	**'Legionella worsleiensis' (GCF_900453045.1)**	15.6	[12.6 - 19.0]	19.9	[17.7 - 22.3]	15.6	[13.1 - 18.5]	1.13
**'Legionella shakespearei DSM 23087' (GCF_000373765.1)**	**'Legionella maioricensis' (GCF_023618015.1)**	15.1	[12.2 - 18.6]	19.9	[17.7 - 22.4]	15.2	[12.7 - 18.1]	2.41
**'Legionella worsleiensis' (GCF_900453045.1)**	**'Legionella rubrilucens' (GCF_900640015.1)**	12.9	[10.2 - 16.2]	19.9	[17.7 - 22.3]	13.3	[10.9 - 16.0]	7.12
**'Legionella antarctica' (GCF_011764505.1)**	**'Legionella worsleiensis' (GCF_900453045.1)**	14.7	[11.9 - 18.1]	19.8	[17.6 - 22.2]	14.9	[12.4 - 17.7]	1.27
**'Legionella worsleiensis' (GCF_900453045.1)**	**'Legionella nagasakiensis' (GCF_900639915.1)**	12.8	[10.1 - 16.1]	19.8	[17.6 - 22.2]	13.2	[10.8 - 15.9]	0.68
**'Legionella maioricensis' (GCF_023618015.1)**	**'Legionella taurinensis' (GCF_900452865.1)**	12.8	[10.1 - 16.1]	19.8	[17.6 - 22.2]	13.2	[10.9 - 16.0]	8.77
**'Legionella shakespearei DSM 23087' (GCF_000373765.1)**	**'Legionella antarctica' (GCF_011764505.1)**	14.7	[11.9 - 18.1]	19.7	[17.5 - 22.1]	14.8	[12.4 - 17.7]	2.56
**'Legionella qingyii' (GCF_003184185.1)**	**'Legionella rubrilucens' (GCF_900640015.1)**	12.7	[10.0 - 16.0]	19.7	[17.5 - 22.1]	13.1	[10.8 - 15.9]	9.73
**'Legionella shakespearei DSM 23087' (GCF_000373765.1)**	**'Legionella fallonii LLAP-10' (GCF_000953135.1)**	14.1	[11.3 - 17.5]	19.7	[17.5 - 22.1]	14.3	[11.9 - 17.1]	3.37
**'Legionella qingyii' (GCF_003184185.1)**	**'Legionella taurinensis' (GCF_900452865.1)**	12.8	[10.1 - 16.1]	19.6	[17.4 - 22.0]	13.2	[10.8 - 15.9]	10.25
**'Legionella septentrionalis' (GCF_003989745.1)**	**'Legionella sp. PATHC038' (GCF_026191355.1)**	12.9	[10.2 - 16.2]	19.6	[17.4 - 22.0]	13.3	[10.9 - 16.1]	3.33
**'Legionella oakridgensis' (GCF_001467925.1)**	**'Legionella bononiensis' (GCF_016786415.1)**	12.9	[10.2 - 16.1]	19.6	[17.4 - 22.0]	13.2	[10.9 - 16.0]	1.79
**'Legionella saoudiensis' (GCF_001465875.1)**	**'Legionella rubrilucens' (GCF_900640015.1)**	12.8	[10.1 - 16.0]	19.5	[17.3 - 21.9]	13.1	[10.8 - 15.9]	8.11
**'Legionella septentrionalis' (GCF_003989745.1)**	**'Legionella rubrilucens' (GCF_900640015.1)**	13.2	[10.5 - 16.5]	19.5	[17.3 - 21.9]	13.5	[11.2 - 16.3]	5.38
**'Legionella shakespearei DSM 23087' (GCF_000373765.1)**	**'Legionella waltersii' (GCF_900187095.1)**	13.9	[11.1 - 17.3]	19.5	[17.3 - 21.9]	14.2	[11.7 - 17.0]	2.52
**'Legionella tucsonensis' (GCF_001468035.1)**	**'Legionella rubrilucens' (GCF_900640015.1)**	13.0	[10.3 - 16.3]	19.5	[17.3 - 21.9]	13.4	[11.0 - 16.2]	10.12
**'Legionella shakespearei DSM 23087' (GCF_000373765.1)**	**'Legionella worsleiensis' (GCF_900453045.1)**	14.9	[12.1 - 18.4]	19.5	[17.3 - 21.9]	15.0	[12.6 - 17.9]	1.28

Query	Subject	d_0	C.I. d_0	d_4	C.I. d_4	d_6	C.I. d_6	Diff. G+C Percent
**'Legionella oakridgensis' (GCF_001467925.1)**	**'Legionella moravica' (GCF_900452715.1)**	12.9	[10.2 - 16.2]	19.5	[17.3 - 21.9]	13.3	[10.9 - 16.0]	0.71
**'Legionella shakespearei DSM 23087' (GCF_000373765.1)**	**'Legionella sp. PATHC039' (GCF_026191275.1)**	14.2	[11.3 - 17.5]	19.4	[17.3 - 21.8]	14.4	[11.9 - 17.2]	3.56
**'Legionella septentrionalis' (GCF_003989745.1)**	**'Legionella worsleiensis' (GCF_900453045.1)**	13.1	[10.4 - 16.4]	19.4	[17.2 - 21.8]	13.4	[11.1 - 16.2]	1.74
**'Legionella oakridgensis' (GCF_001467925.1)**	**'Legionella sp. PATHC032' (GCF_026191185.1)**	13.2	[10.4 - 16.5]	19.4	[17.3 - 21.8]	13.5	[11.1 - 16.3]	2.81
**'Legionella shakespearei DSM 23087' (GCF_000373765.1)**	**'Legionella norrlandica' (GCF_000770585.1)**	13.9	[11.1 - 17.2]	19.4	[17.2 - 21.8]	14.1	[11.7 - 16.9]	4.23
**'Fluoribacter gormanii' (GCF_900156395.1)**	**'Legionella taurinensis' (GCF_900452865.1)**	12.8	[10.1 - 16.1]	19.3	[17.1 - 21.7]	13.2	[10.8 - 15.9]	9.97
**'Legionella norrlandica' (GCF_000770585.1)**	**'Legionella worsleiensis' (GCF_900453045.1)**	13.9	[11.1 - 17.2]	19.2	[17.0 - 21.6]	14.1	[11.7 - 16.9]	2.94
**'Legionella sp. PATHC032' (GCF_026191185.1)**	**'Legionella rubrilucens' (GCF_900640015.1)**	13.0	[10.3 - 16.3]	19.2	[17.0 - 21.6]	13.3	[11.0 - 16.1]	9.51
**'Legionella shakespearei DSM 23087' (GCF_000373765.1)**	**'Legionella pneumophila' (GCF_001941585.1)**	14.1	[11.3 - 17.5]	19.2	[17.0 - 21.6]	14.3	[11.9 - 17.1]	3.37
**'Legionella oakridgensis' (GCF_001467925.1)**	**'Legionella taurinensis' (GCF_900452865.1)**	13.1	[10.4 - 16.4]	19.2	[17.0 - 21.6]	13.4	[11.1 - 16.2]	7.22
**'Legionella septentrionalis' (GCF_003989745.1)**	**'Legionella cherrii' (GCF_900635815.1)**	12.9	[10.2 - 16.2]	19.2	[17.0 - 21.6]	13.3	[10.9 - 16.0]	3.26
**'Legionella septentrionalis' (GCF_003989745.1)**	**'Legionella bozemanae' (GCF_900640135.1)**	12.9	[10.2 - 16.2]	19.2	[17.0 - 21.6]	13.3	[10.9 - 16.0]	4.24
**'Legionella maioricensis' (GCF_023618015.1)**	**'Legionella rubrilucens' (GCF_900640015.1)**	12.8	[10.1 - 16.1]	19.2	[17.0 - 21.6]	13.1	[10.8 - 15.9]	8.25
**'Fluoribacter dumoffii NY 23' (GCF_000236165.1)**	**'Legionella septentrionalis' (GCF_003989745.1)**	13.0	[10.3 - 16.3]	19.1	[16.9 - 21.5]	13.4	[11.0 - 16.1]	2.52
**'Legionella drancourtii LLAP12' (GCF_000162755.2)**	**'Legionella septentrionalis' (GCF_003989745.1)**	12.9	[10.2 - 16.2]	18.9	[16.7 - 21.2]	13.3	[10.9 - 16.0]	2.91
**'Fluoribacter gormanii' (GCF_900156395.1)**	**'Legionella rubrilucens' (GCF_900640015.1)**	12.9	[10.2 - 16.2]	18.9	[16.8 - 21.3]	13.2	[10.9 - 16.0]	9.45
**'Legionella oakridgensis' (GCF_001467925.1)**	**'Legionella septentrionalis' (GCF_003989745.1)**	13.4	[10.7 - 16.8]	18.9	[16.7 - 21.3]	13.7	[11.3 - 16.5]	1.32
**'Legionella septentrionalis' (GCF_003989745.1)**	**'Legionella taurinensis' (GCF_900452865.1)**	13.1	[10.4 - 16.5]	18.8	[16.6 - 21.2]	13.5	[11.1 - 16.3]	5.9
**'Legionella septentrionalis' (GCF_003989745.1)**	**'Legionella maioricensis' (GCF_023618015.1)**	12.8	[10.1 - 16.0]	18.8	[16.6 - 21.2]	13.1	[10.8 - 15.9]	2.87
**'Legionella bononiensis' (GCF_016786415.1)**	**'Legionella nagasakiensis' (GCF_900639915.1)**	13.0	[10.3 - 16.3]	18.8	[16.6 - 21.1]	13.4	[11.0 - 16.1]	2.05
**'Legionella septentrionalis' (GCF_003989745.1)**	**'Legionella sp. PATHC032' (GCF_026191185.1)**	13.1	[10.3 - 16.4]	18.8	[16.7 - 21.2]	13.4	[11.0 - 16.2]	4.13
**'Legionella rubrilucens' (GCF_900640015.1)**	**'Legionella bozemanae' (GCF_900640135.1)**	13.0	[10.3 - 16.3]	18.8	[16.6 - 21.1]	13.3	[11.0 - 16.1]	9.62

Query	Subject	d_0	C.I. d_0	d_4	C.I. d_4	d_6	C.I. d_6	Diff. G+C Percent
**'Legionella shakespearei DSM 23087' (GCF_000373765.1)**	**'Legionella septentrionalis' (GCF_003989745.1)**	12.9	[10.2 - 16.2]	18.7	[16.5 - 21.1]	13.3	[10.9 - 16.1]	0.46
**'Legionella saoudiensis' (GCF_001465875.1)**	**'Legionella septentrionalis' (GCF_003989745.1)**	13.1	[10.3 - 16.4]	18.5	[16.3 - 20.9]	13.4	[11.0 - 16.2]	2.73
**'Legionella oakridgensis' (GCF_001467925.1)**	**'Legionella rubrilucens' (GCF_900640015.1)**	12.9	[10.2 - 16.2]	18.5	[16.4 - 20.9]	13.3	[10.9 - 16.0]	6.7
**'Legionella steelei' (GCF_001468005.1)**	**'Legionella septentrionalis' (GCF_003989745.1)**	12.9	[10.2 - 16.2]	18.5	[16.4 - 20.9]	13.3	[10.9 - 16.1]	3.39
**'Legionella shakespearei DSM 23087' (GCF_000373765.1)**	**'Legionella nagasakiensis' (GCF_900639915.1)**	12.9	[10.2 - 16.2]	18.4	[16.2 - 20.8]	13.2	[10.9 - 16.0]	0.6
**'Legionella shakespearei DSM 23087' (GCF_000373765.1)**	**'Legionella oakridgensis' (GCF_001467925.1)**	12.8	[10.1 - 16.0]	18.2	[16.1 - 20.6]	13.1	[10.8 - 15.9]	0.86
**'Legionella shakespearei DSM 23087' (GCF_000373765.1)**	**'Legionella taurinensis' (GCF_900452865.1)**	12.9	[10.2 - 16.2]	18.0	[15.9 - 20.4]	13.2	[10.9 - 16.0]	6.36
**'Legionella taurinensis' (GCF_900452865.1)**	**'Legionella nagasakiensis' (GCF_900639915.1)**	13.2	[10.5 - 16.5]	18.0	[15.9 - 20.4]	13.5	[11.1 - 16.3]	6.96
**'Legionella septentrionalis' (GCF_003989745.1)**	**'Fluoribacter gormanii' (GCF_900156395.1)**	12.8	[10.2 - 16.1]	17.9	[15.8 - 20.3]	13.2	[10.9 - 16.0]	4.08
**'Legionella qingyii' (GCF_003184185.1)**	**'Legionella septentrionalis' (GCF_003989745.1)**	12.9	[10.2 - 16.2]	17.8	[15.6 - 20.1]	13.2	[10.9 - 16.0]	4.35
**'Legionella shakespearei DSM 23087' (GCF_000373765.1)**	**'Legionella rubrilucens' (GCF_900640015.1)**	12.9	[10.2 - 16.2]	17.5	[15.4 - 19.9]	13.2	[10.9 - 16.0]	5.84

Table 4: Strains in your dataset

Joint dataset of automatically determined closest type strains (if this mode was chosen), manually selected type strains (if selected accordingly) and the provided user strains, if provided (marked in yellow).

Strain	Authority	Other deposits	Synonyms	Base pairs	Percent G+C	No. proteins	Goldstamp	Bioproject accession	Biosample accession	Assembly accession	IMG OID
'Legionella anisa' (GCA_019930885.1)				4448073	38.3	3986					
'Legionella drancourtii LLAP12' (GCF_000162755.2)				4070090	39.2	3785					
'Fluoribacter dumoffii NY 23' (GCF_000236165.1)				3894641	39.6	3468					
'Legionella shakespearei DSM 23087' (GCF_000373765.1)				3507313	41.7	2987					
'Legionella norrlandica' (GCF_000770585.1)				3070855	37.5	3026					
'Legionella fallonii LLAP-10' (GCF_000953135.1)				4434890	38.3	3740					
'Legionella saoudiensis' (GCF_001465875.1)				3846014	39.4	3346					
'Legionella oakridgensis' (GCF_001467925.1)				2668211	40.8	2507					
'Legionella steelei' (GCF_001468005.1)				4227172	38.8	3729					

Strain	Authority	Other deposits	Synonyms	Base pairs	Percent G+C	No. proteins	Goldstamp	Bioproject accession	Biosample accession	Assembly accession	IMG OID
'Legionella tucsonensis' (GCF_001468035.1)				3356485	37.4	2973					
'Legionella santicrucis' (GCF_001468135.1)				4820916	36.7	4294					
'Legionella pneumophila' (GCF_001941585.1)				3409143	38.3	3024					
'Legionella qingyii' (GCF_003184185.1)				5241559	37.8	4197					
'Legionella septentrionalis' (GCF_003989745.1)				2660547	42.2	2491					
'Legionella antarctica' (GCF_011764505.1)				3927137	39.1	3801					
'Legionella bononiensis' (GCF_016786415.1)				4136543	39.0	3554					
'Legionella longbeachae' (GCF_019930685.1)				4165456	37.1	3587					
'Legionella resiliens' (GCF_021344005.1)				3906083	38.2	3408					
'Legionella maioricensis' (GCF_023618015.1)				3967144	39.3	3491					
'Legionella sp. PATHC035' (GCF_026191115.1)				3895788	39.4	3367					

Strain	Authority	Other deposits	Synonyms	Base pairs	Percent G+C	No. proteins	Goldstamp	Bioproject accession	Biosample accession	Assembly accession	IMG OID
'Legionella sp. PATHC032' (GCF_026191185.1)				3448333	38.0	3048					
'Legionella sp. PATHC039' (GCF_026191275.1)				3517029	38.1	3178					
'Legionella sp. PATHC038' (GCF_026191355.1)				4275963	38.8	3894					
'Fluoribacter gormanii' (GCF_900156395.1)				3813119	38.1	3347					
'Legionella waltersii' (GCF_900187095.1)				3735697	39.2	3461					
'Legionella cincinnatiensis' (GCF_900452415.1)				4098995	36.9	3497					
'Legionella gratiana' (GCF_900452545.1)				3976384	37.0	3461					
'Legionella quateirensis' (GCF_900452695.1)				4281523	39.1	3612					
'Legionella moravica' (GCF_900452715.1)				3825910	40.1	3215					
'Legionella steigerwaltii' (GCF_900452835.1)				3998094	38.3	3513					
'Legionella taurinensis' (GCF_900452865.1)				3151601	48.1	2893					

Strain	Authority	Other deposits	Synonyms	Base pairs	Percent G+C	No. proteins	Goldstamp	Bioproject accession	Biosample accession	Assembly accession	IMG OID
'Legionella wadsworthii' (GCF_900452925.1)				3602617	38.1	3187					
'Legionella worsleiensis' (GCF_900453045.1)				3161811	40.4	2705					
'Legionella parisiensis' (GCF_900461585.1)				4202140	38.0	3699					
'Legionella cherrii' (GCF_900635815.1)				3729541	38.9	3281					
'Legionella sainthelensi' (GCF_900637685.1)				4160933	37.1	3748					
'Legionella nagasakiensis' (GCF_900639915.1)				2693561	41.1	2592					
'Legionella rubrilucens' (GCF_900640015.1)				3186910	47.5	2936					
'Legionella bozemanae' (GCF_900640135.1)				4130121	37.9	3710					

Methods, Results and References

The genome sequence data were uploaded to the Type (Strain) Genome Server (TYGS), a free bioinformatics platform available under https://tygs.dsmz.de, for a whole genome-based taxonomic analysis [1]. The analysis also made use of recently introduced methodological updates and features [2,3]. Information on nomenclature, synonymy and associated taxonomic literature was provided by TYGS's sister database, the List of Prokaryotic names with Standing in Nomenclature (LPSN, available at https://lpsn.dsmz.de) [2,3]. The results were provided by the TYGS on 2026-05-02. The TYGS analysis was subdivided into the following steps:

Determination of closely related type strains

Determination of closest type strain genomes was done in two complementary ways: First, all user genomes were compared against all type strain genomes available in the TYGS database via the MASH algorithm, a fast approximation of intergenomic relatedness [4], and, the ten type strains with the smallest MASH distances chosen per user genome. Second, an additional set of ten closely related type strains was determined via the 16S rDNA gene sequences. These were extracted from the user genomes using RNAmmer [5] and each sequence was subsequently BLASTed [6] against the 16S rDNA gene sequence of each of the currently 24384 type strains available in the TYGS database. This was used as a proxy to find the best 50 matching type strains (according to the bitscore) for each user genome and to subsequently calculate precise distances using the Genome BLAST Distance Phylogeny approach (GBDP) under the algorithm 'coverage' and distance formula d_5 [7]. These distances were finally used to determine the 10 closest type strain genomes for each of the user genomes.

Pairwise comparison of genome sequences

For the phylogenomic inference, all pairwise comparisons among the set of genomes were conducted using GBDP and accurate intergenomic distances inferred under the algorithm 'trimming' and distance formula d_5 [7]. 100 distance replicates were calculated each. Digital DDH values and confidence intervals were calculated using the recommended settings of the GGDC 4.0 [2,7].

Phylogenetic inference

The resulting intergenomic distances were used to infer a balanced minimum evolution tree with branch support via FASTME 2.1.6.1 including SPR postprocessing [8]. Branch support was inferred from 100 pseudo-bootstrap replicates each. The trees were rooted at the midpoint [9] and visualized with PhyD3 [10].

Type-based species and subspecies clustering

The type-based species clustering using a 70% dDDH radius around each of the 0 type strains was done as previously described [1]. The resulting groups are shown in Table 1 and 4. Subspecies clustering was done using a 79% dDDH threshold as previously introduced [11].

Results

Type-based species and subspecies clustering

The resulting species and subspecies clusters are listed in Table 4, whereas the taxonomic identification of the query strains is found in Table 1. Briefly, the clustering yielded species clusters and the provided query strains were assigned to of these. Moreover, user strains were located in of subspecies clusters.

Figure caption SSU tree

Figure 1. Tree inferred with FastME 2.1.6.1 [8] from GBDP distances calculated from 16S rDNA gene sequences. The branch lengths are scaled in terms of GBDP distance formula d_5. The numbers above branches are GBDP pseudo-bootstrap support values > 60 % from 100 replications, with an average branch support of 91.9 %. The tree was rooted at the midpoint [9].

Figure caption genome tree

Figure 2. Tree inferred with FastME 2.1.6.1 [8] from GBDP distances calculated from genome sequences. The branch lengths are scaled in terms of GBDP distance formula d_5. The numbers above branches are GBDP pseudo-bootstrap support values > 60 % from 100 replications, with an average branch support of 57.1 %. The tree was rooted at the midpoint [9].

References

[1] Meier-Kolthoff JP, Göker M. TYGS is an automated high-throughput platform for state-of-the-art genome-based taxonomy. Nat. Commun. 2019;10: 2182. DOI: 10.1038/s41467-019-10210-3

[2] Meier-Kolthoff JP, Sardà Carbasse J, Peinado-Olarte RL, Göker M. TYGS and LPSN: a database tandem for fast and reliable genome-based classification and nomenclature of prokaryotes. Nucleic Acid Res. 2022;50: D801–D807. DOI: 10.1093/nar/gkab902

[3] Freese HM, Meier-Kolthoff JP, Sardà Carbasse J, Afolayan AO, Göker M. TYGS and LPSN in 2025: a Global Core Biodata Resource for genome-based classification and nomenclature of prokaryotes within DSMZ Digital Diversity. Nucleic Acid Res. 2025, gkaf1110. DOI: 10.1093/nar/gkaf1110

[4] Ondov BD, Treangen TJ, Melsted P, et al. Mash: Fast genome and metagenome distance estimation using MinHash. Genome Biol 2016;17: 1–14. DOI: 10.1186/s13059-016-0997-x

[5] Lagesen K, Hallin P. RNAmmer: consistent and rapid annotation of ribosomal RNA genes. Nucleic Acids Res. Oxford Univ Press; 2007;35: 3100–3108. DOI: 10.1093/nar/gkm160

[6] Camacho C, Coulouris G, Avagyan V, Ma N, Papadopoulos J, Bealer K, et al. BLAST+: architecture and applications. BMC Bioinformatics. 2009;10: 421. DOI: 10.1186/1471-2105-10-421

[7] Meier-Kolthoff JP, Auch AF, Klenk H-P, Göker M. Genome sequence-based species delimitation with confidence intervals and improved distance functions. BMC Bioinformatics. 2013;14: 60. DOI: 10.1186/1471-2105-14-60

[8] Lefort V, Desper R, Gascuel O. FastME 2.0: A comprehensive, accurate, and fast distance-based phylogeny inference program. Mol Biol Evol. 2015;32: 2798–2800. DOI: 10.1093/molbev/msv150

[9] Farris JS. Estimating phylogenetic trees from distance matrices. Am Nat. 1972;106: 645–667.

[10] Kreft L, Botzki A, Coppens F, Vandepoele K, Van Bel M. PhyD3: A phylogenetic tree viewer with extended phyloXML support for functional genomics data visualization. Bioinformatics. 2017;33: 2946–2947. DOI: 10.1093/bioinformatics/btx324

[11] Meier-Kolthoff JP, Hahnke RL, Petersen J, Scheuner C, Michael V, Fiebig A, et al. Complete genome sequence of DSM 30083^T, the type strain (U5/41^T) of *Escherichia coli*, and a proposal for delineating subspecies in microbial taxonomy. Stand Genomic Sci. 2014;9: 2. DOI: 10.1186/1944-3277-9-2
