# Supplementary material for: A Modern Framework for Identifying Novel Environmental Legionella Species
Source: Life (Basel). 2026 Jul 17;16(7):1187. doi: 10.3390/life16071187 (PMC13412451; doi:10.3390/life16071187)
Supplement: Supplementary file 1 [file life-16-01187-s001.zip › Table S2.pdf]

Table S2. General characteristics of the genome assemblies of the isolates. Assemblies were obtained from NCBI database.

Chararacteristic	PATHC032	PATHC039	PATHC035	PATHC038^T (*L. sheltonii*)
**Genome size (Mb)**	3.4	3.5	3.9	4.3
**No. of contigs**	2	3	2	63
**GC content (mol%)**	38.0	38.0	39.5	39.0
**Contig N50 (kb)**	3400	3300	3700	235.7
**Genome coverage (×)**	88.6157	170.729	117.918	343.964
**Completeness (%)**	99.73	99.59	99.76	99.76
**Contamination (%)**	0.18	0.46	1.55	1.38
**Number of coding sequences**	2996	3109	3331	3757
**NCBI RefSeq assembly**	GCF_026191185.1	GCF_026191275.1	GCF_026191115.1	GCF_026191355.1
